# Hemochromatosis neural archetype reveals iron disruption in motor circuits

**DOI:** 10.1126/sciadv.adp4431

**Published:** 2024-11-22

**Authors:** Robert Loughnan, Jonathan Ahern, Mary Boyle, Terry L. Jernigan, Donald J. Hagler, John R. Iversen, Oleksandr Frei, Diana M. Smith, Ole Andreassen, Noah Zaitlen, Leo Sugrue, Wesley K. Thompson, Anders Dale, Andrew J. Schork, Chun Chieh Fan

**Affiliations:** ^1^ Department of Cognitive Science, University of California, San Diego, 9500 Gilman Drive, La Jolla, CA 92093, USA.; ^2^ Center for Human Development, University of California, San Diego, 9500 Gilman Drive, La Jolla, CA 92161, USA.; ^3^ Center for Multimodal Imaging and Genetics, University of California, San Diego School of Medicine, 9500 Gilman Drive, La Jolla, CA 92037, USA.; ^4^ Center for Population Neuroscience and Genetics, Laureate Institute for Brain Research, Tulsa, OK 74103, USA.; ^5^ Department of Psychiatry, University of California, San Diego School of Medicine, 9500 Gilman Drive, La Jolla, CA 92037, USA.; ^6^ Department of Radiology, University of California, San Diego School of Medicine, 9500 Gilman Drive, La Jolla, CA 92037, USA.; ^7^ Swartz Center for Computational Neuroscience, University of California, San Diego, 9500 Gilman Drive, La Jolla, CA 92161, USA.; ^8^ NORMENT Centre, Division of Mental Health and Addiction, Oslo University Hospital and Institute of Clinical Medicine, University of Oslo, Oslo, Norway.; ^9^ Medical Scientist Training Program, University of California, San Diego School of Medicine, 9500 Gilman Drive, La Jolla, CA 92093, USA.; ^10^ Department of Neurology, University of California Los Angeles, Los Angeles, CA 90095, USA.; ^11^ Department of Radiology and Biomedical Imaging and Department of Psychiatry, University of California, San Francisco, 505 Parnassus Avenue, San Francisco, CA 94143, USA.; ^12^ Department of Neuroscience, University of California, San Diego School of Medicine, 9500 Gilman Drive, La Jolla, CA 92037, USA.; ^13^ Institute of Biological Psychiatry, Mental Health Center–Sct Hans, Copenhagen University Hospital, Copenhagen, Denmark.; ^14^ Section for Geogenetics, GLOBE Institute, Faculty of Health and Medical Sciences, Copenhagen University, Copenhagen, Denmark.

## Abstract

Our understanding of brain iron regulation and its disruption in disease is limited. Excess iron affects motor circuitry, contributing to Parkinson’s disease (PD) risk. The molecular mechanisms regulating central iron levels, beyond a few well-known genes controlling peripheral iron, remain unclear. We generated scores based on the archetypal brain iron accumulation observed in magnetic resonance imaging scans of individuals with excessive dietary iron absorption and hemochromatosis risk. Genome-wide analysis revealed that this score is highly heritable, identifying loci associated with iron homeostasis, and driven by peripheral iron levels. Our score predicted gait abnormalities and showed a U-shaped relationship with PD risk, identifying individuals with threefold increased risk. These results establish a hormetic relationship between brain iron and PD risk, where central iron levels are strongly determined by genetics via peripheral iron. This framework combining forward and reverse genetics is a powerful study design to understand genomic drivers underlying high dimensional phenotypes.

## Teaser

Genetic determinants of central iron dysregulation and related Parkinson’s disease risk are uncovered.

## INTRODUCTION

Iron is an essential element required for the body to function properly. The role of iron in numerous biochemical reactions stems from its dual function as an electron mediator, serving as both a donor and acceptor, making it indispensable for life. In the brain, iron is particularly important for neuronal development, myelination, and synthesis of neurotransmitters such as dopamine (*1*). For these reasons, during early life, there is a substantial demand for iron, and not meeting this requirement can lead to iron deficiency anemia (*2*). More broadly, iron deficiency in early life can have profound and long-term effects on motor skills, cognitive function, and socio-emotional behavior, underscoring the critical role of iron in healthy development. Conversely, too much iron can be toxic and dangerous also (*3*). Through the generation of reactive oxygen species and iron-dependent cell death, known as ferroptosis, iron overload can cause widespread damage to healthy tissue throughout the body (*4*–*6*). As iron depletion and overload can both cause substantial damage, the body must orchestrate a careful balancing act to regulate iron levels. Although we have a good understanding of how this is achieved systemically through the interaction of hepcidin and ferroportin, our understanding of mechanisms controlling brain iron remains incomplete (*7*–*9*). Elucidating these processes is of importance to gain insight into metabolic homeostasis in the brain.

Magnetic resonance imaging (MRI) provides a noninvasive method to accurately estimate iron concentrations in the brain (*10*). This has enabled researchers to study trajectories of central iron across the life span uncovering a pattern of iron accumulation throughout regions of the basal ganglia in later life (*11*)—possibly contributing to senescence. MRI and postmortem studies have also implicated iron dysregulation in numerous neurodegenerative diseases, most notably in movement disorders such as Parkinson’s disease (PD) and neurodegeneration with brain iron accumulation (NBIA) (*1*, *12*, *13*). These findings have led researchers to attempt to use iron as a therapeutic target for PD and related disorders through the use of iron chelators which bind to and remove excess iron in the blood (*14*). Clinical trials of iron chelators have shown promise for some patients with movement disorders while exacerbating symptoms in others (*15*–*17*). Our inability to explain this heterogeneity in treatment outcomes points to a poor understanding of iron dyshomeostasis occurring in movement disorders. Recent work has aimed to understand the genetic determinants of regional iron accumulation in the brain (*18*); however, this has led to little further insight into abhorrent regulatory mechanisms in disease. There is therefore a critical need to define biologically grounded phenotypes when studying brain imaging signals (*19*).

Here, we adopt a pioneering approach to study brain iron, its genetic determinants, and neurodegenerative manifestations. In this approach, we leverage the regional brain iron accumulation associated with the most prevalent genetic risk factor for excessive iron absorption in the gut, known as *C282Y* homozygosity. This genotype results in misfolding of the HFE (human homeostatic iron regulator) protein (*20*, *21*), which is critical for signaling of hepcidin—the master regulator of iron balance. With this signaling, impaired *C282Y* homozygotes have inappropriately low hepcidin levels resulting in excessive iron absorption and increased risk of developing hereditary hemochromatosis (*21*). This genotype was selected in the current work due to (i) to its high allelic frequency in the studied population (*22*) (homozygosity rate: ~1/200), (ii) large genetic effect (*23*, *24*), and (iii) recent findings from our group showcasing this genotype’s role in motor circuit disruption. In this recent work, we demonstrated that it exhibits an MRI pattern showcasing substantial localized iron accumulation in the brain’s motor circuits, specifically the basal ganglia and cerebellum (*25*). Notably, this genotype also corresponds to a twofold increase in male-specific risk of developing movement disorders. Here, we generate a novel brain endophenotype, based on *C282Y*, which captures a continuum of brain iron dysregulation and which we show to be predictive of risk for movement disorders. To operationalize our recent MRI findings of *C282Y* into an endophenotype, which we refer to as the “Hemochromatosis Brain,” we trained a classifier to predict *C282Y* homozygosity status from T2-weighted brain MRI scans, a modality sensitive to iron accumulation (*10*). We performed this training in a subsample of 960 individuals taken from UK Biobank (UKB). We then deploy this classifier on 35,283 independent MRI images, also from UKB, none of whom are *C282Y* homozygotes, to generate a PolyVoxel Score (PVS) (*26*) for each individual capturing the degree to which they resemble the archetypal Hemochromatosis Brain. The resulting PVS enables us to answer two questions: First, does a neural archetype learned from an extreme case of iron dysregulation inform us about the genetic drivers of iron homeostasis of the human brain more generally? Second, does the degree of brain iron buildup, as encapsulated by the Hemochromatosis Brain, predict risk for movement disorders? This work is an important step in understanding the genetic determinants of brain iron dysregulation and in identifying putative iron-related subgroups of movement disorders.

## RESULTS

### Learning from the Hemochromatosis Brain

**Fig. 1.**
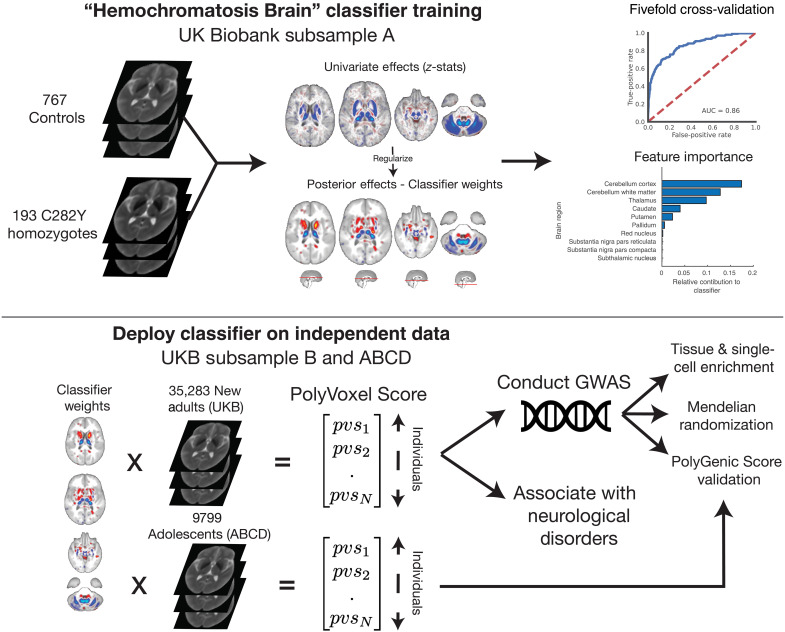
Overview of study design. **Top**: Hemochromatosis Brain classifier training in subsample A of UKB to differentiate controls from C282Y homozygotes from T2-weighted MRI scans, with univariate and regularized classifier weights, classifier performance (receiver operator characteristic), and feature importance by brain region. **Bottom**: Deploy classifier in subsample B (UKB) and ABCD Study to generate PVS capturing Hemochromatosis Brain liability. In subsample B, we then conduct a GWAS to find variants associated with this PVS liability scale. Using these GWAS results, we perform tissue and single-cell enrichment analysis, Mendelian randomization (MR), and PolyGenic Score (PGS) validation against the PVS generated from the ABCD Study. Last, we test this PVS against neurological disorders within UKB. AUC, area under the curve.

Figure 1 presents a graphical workflow of the analysis performed in this paper, with table S1 giving a demographic breakdown of each subsample. Similarly, for the Adolescent Brain Cognitive Development (ABCD) sample used in subsequent analysis table S2 presents a demographic breakdown. Performing fivefold cross-validation in subsample A of UKB, consisting of 193 *C282Y* homozygotes and 767 covariate-matched controls, established that the Hemochromatosis Brain classifier can predict *C282Y* homozygosity status with high accuracy from T2-weighted scans (attaining an area under the curve = 0.86). Figure S1 shows results of hyperparameter tuning. Figure 1 displays classifier weights and feature importance of the classifier by brain region (see also fig. S2). As expected, univariate statistics show strong resemblance to previous work describing a *C282Y* homozygote effect on a T2-weighted signal (*25*). Feature importance of posterior effects indicates that the cerebellum, thalamus, putamen, and caudate have the largest contributions to the PVS (regions of interest defined in table S3). Figure S2C displays the Pearson correlation of the PVS with mean R2* intensity values (indicative of iron concentration), broken down by region of interest, to provide a complementary understanding of feature importance. We see that R2* values for regions, such as the substantia nigra, show significant correlations with the PVS, indicating that iron accumulation variability in these regions is still being captured, despite small contribution from weights seen in fig. S2B.

### Evaluating the validity of the Hemochromatosis Brain with genetic analyses

**Fig. 2.**
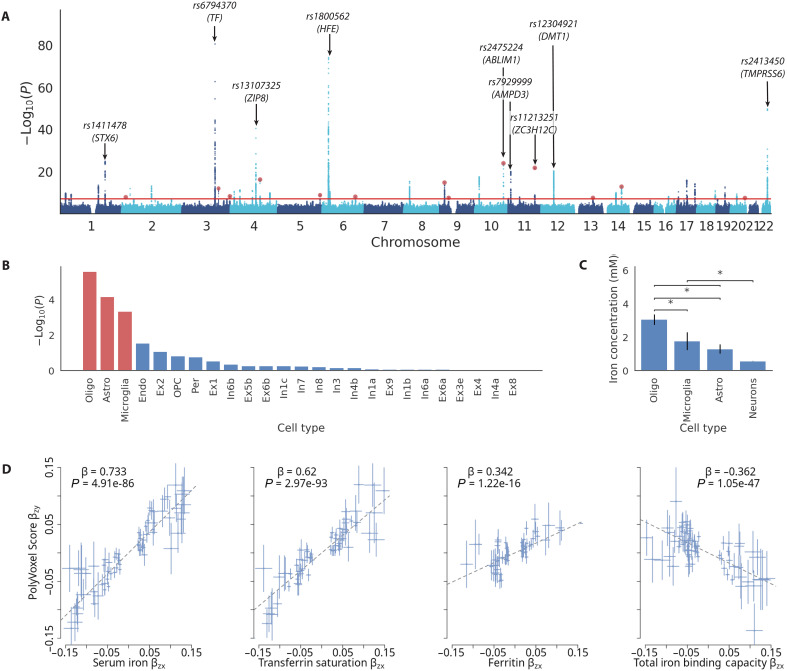
Results of GWAS on PVS of Hemochromatosis Brain in subsample B. (**A**) Manhattan plot GWAS result with peaks annotated—a total of 42 loci are discovered (see supplementary data tables for full list). Red dots indicate 13 novel loci. (**B**) Brain cell type enrichment of GWAS signal using FUMA, and red bars indicate FDR significantly enriched cell types. (**C**) Iron concentration (measured by using x-ray spectrometry) of cell types in rat brain with permissions from Reinert *et al.* (*38*). *P < 0.001. (**D**) MR results, using GSMR, to quantify strength of causal relationship between peripheral blood iron markers and brain iron accumulation as measured by Hemochromatosis Brain PVS. Each plot shows results of conditioning on peripheral blood iron markers as exposure and PVS as outcome, and reverse direction GSMR results are shown in fig. S28. See table S5 for full MR results. Error bars in (C) and (D) represent SEs in estimates.

We then sought to evaluate the validity of our scoring system by conducting a series of genetic analyses based on the Hemochromatosis Brain PVS in an independent set of individuals from UKB. First, we performed a genome-wide association study (GWAS) in subsample B with the PVS as the phenotype of interest. For discovery, we restricted this analysis to a single homogeneous ancestry group and used the remaining individuals for validation. This resulted in a discovery sample size of 30,709 European ancestry individuals (EUR) and 4608 non-European ancestry individuals for replication. This analysis revealed 42 genome-wide significant loci (13 of these being novel for any reported trait) associated with the Hemochromatosis Brain phenotype and high heritability: $h_{\text{ldsc}}^{2}=0.381$ (SE = 0.033)—see Fig. 2A and figs. S3 to S25. We found that this heritability was substantially higher than the heritability estimated for peripheral blood iron markers ($h_{\text{ldsc}}^{2}$ between 0.029 and 0.056)—see extended data tables. Among the top hits, we found many that are known regulators of iron homeostasis: For example, *rs6794370* (*TF*; *P* = 2.43 × 10^−81^) transferrin is the principal glycoprotein responsible for mediating transport of iron through blood plasma; *rs1800562* (*HFE*; *P* = 6.93 × 10^−75^) is the same *C282Y* mutation on which the classifier was trained (here rediscovered with the heterozygotes); *rs2413450* (*TMPRSS6*, *P* = 2.52 × 10^−50^), *TMPRSS6* is part of production signaling pathway of hepcidin, the key hormonal regulator of iron absorption in humans; *rs13107325* (*P* = 3.66 × 10^−41^) and *rs12304921* (*P* = 5.05 × 10^−21^) are linked, respectively, to metal transporters *ZIP8* and *DMT1*, the latter being a gene highly expressed in the gut and previously linked with microcytic anemia with iron overload (*27*, *28*).

Among the 13 novel loci we discovered was *rs73210039* (4.47 × 10^−9^) proximal to *TFRC*, transferrin receptor 1, a gene known to play a critical role in cellular iron uptake with a variable expression pattern across the brain (*29*). This locus was not implicated in recent GWAS of peripheral iron markers (*30*); however, a nearby marker, *rs3817672* (also proximal to *TFRC*), was found to be associated with serum transferrin and total iron binding capacity. This indicates that these polymorphisms appear to have differential effects on central versus systemic iron through *TFRC*. Another novel locus, *rs62560298* (*P* = 1.64 × 10^−15^), proximal to *DENND4C*, is a member of the retromer complex family that deals with recycling of metals in the body, which has been hypothesized to be implicated in neurodegeneration such as PD (*31*). A further novel locus was detected at *rs112593750* (*P* = 9.82 × 10^−13^) intronic to *CPHL1P*, a pseudogene whose functioning homolog is hephaestin—a gene necessary for effective iron transport in the intestines and which has been found to be mutated in sex-linked anemia (*32*).

We found that our discovered single-nucleotide polymorphisms (SNPs) showed greatest overlap with previously studied traits relating to iron/red blood cells, brain/cognition, and non-iron blood markers (fig. S26 and table S4), with only one SNP (*rs13107325*) previously linked with PD (*33*). Analysis of rare genetic variants using exome burden testing revealed two genes associated with the PVS: *TF* (*P* = 5.21 × 10^−8^) and *OR1J4* (*P* = 1.35 × 10^−6^). See extended data tables for full summary of GWAS results.

We used Linkage Disequilibrium Score Regression (LDSC) (*34*) on common variant GWAS results to test whether the genetic architecture overlapped with any related phenotypes—see Materials and Methods. For this analysis, we found that the Hemochromatosis Brain was negatively genetically correlated only with intracranial volume (*r*_g_ = −0.21, *z* = −5.19, and *P* = 1.7 × 10^−6^), indicating a shared genetic architecture between these two traits. Unexpectedly, we found that there were no significant genetic correlations between the Hemochromatosis Brain and blood iron–related traits (fig. S27), indicating that the genetic architecture of brain iron accumulation, captured by the PVS, is distinct from peripheral iron. We additionally performed S-LDSC (stratified-LDSC) across 489 tissue-specific chromatin-based annotations across the body from peaks for six epigenetic marks (*35*). Results revealed that the hippocampus (*H3K27ac*, *P*_FDR_ = 0.014 and *H3K4me1*, *P*_FDR_ = 0.025) and substantia nigra (*H3k27ac*, *P*_FDR_ = 0.036) displayed a significantly enriched signal. Performing cell type enrichment using Functional Mapping and Annotation (FUMA) (*36*) of Hemochromatosis Brain GWAS using cell types defined from PsychENCODE (*37*) displayed an enrichment for glial cells: oligodendrocytes (*P*_FDR_ = 6.62 × 10^−5^), astrocytes (*P*_FDR_ = 1.7 × 10^−3^), and microglia (*P*_FDR_ = 1.8 × 10^−2^) and somewhat unexpectedly no enrichment for neuronal cells—see Fig. 2B. This pattern of enrichment exhibits a similar pattern followed by iron concentrations calculated using x-ray spectroscopy (*38*)—displayed in Fig. 2C for comparison.

Next, we apply Mendelian Randomization (MR) (*39*) to our GWAS results. Through the use of genetic variants as instrumental variables, this method aims to infer causality between an exposure and an outcome in so doing mitigating confounding and reverse causation. MR results between peripheral blood iron markers (*30*) and the PVS revealed evidence for a strong causal relationship leading from serum iron (β_std_ = 0.73, *P* = 4.91 × 10^−86^) and transferrin saturation (β_std_ = 0.62, *P* = 2.97 × 10^−93^) to brain iron accumulation (Fig. 2D) with no evidence for the reverse relationship (fig. S28). We additionally found significant, although weaker, MR associations of total iron binding capacity (β_std_ = −0.36, *P* = 1.05 × 10^−47^) and ferritin (β_std_ = 0.342, *P* = 1.22 × 10^−16^) to brain iron accumulation as captured by the PVS, with again no evidence for the reverse direction (fig. S28). Together, these indicate evidence for a causal link of peripheral serum iron and transferrin saturation leading to variability in brain iron accumulation measured by the PVS. Despite significant phenotypic associations between PVS and PD (see the next section), we did not find any significant MR associations between PVS and PD or peripheral blood iron markers and PD (fig. S28). Table S5 presents a complete list of results from MR analysis.

To test replication of our GWAS discoveries of brain iron differences, we performed (i) replication analysis in a non-overlapping set of adults from UKB (subsample B) and (ii) generalization by generating a PVS in adolescents taken from the ABCD Study. Within our GWAS discovery, we found 281 independent significant SNPs (*r*_LD_ < 0.6). We found strong evidence of replication of these SNPs within our validation cohort of 4608 non-European ancestry individuals from subsample B (table S1) with high correlation of β estimates between these two cohorts (*r* = 0.90, *P* < 1.28 × 10^−103^)—see fig. S29. Sign concordance between discovery and replication cohorts was high for independent significant SNPs (94.6%, *P* = 1.0 × 10^−59^) and for 13 novel loci (92.3%, *P* = 0.003). To test the generalization of our GWAS signal, we evaluated the performance of a PolyGenic Score (PGS) to predict a PVS generated in 9799 individuals from the ABCD Study (aged 8 to 14 years old, i.e., more than 50 years younger than our training sample). Evaluating performance in separate ancestry strata, we found that the PGS significantly predicted the PVS [EUR: *r*^2^ = 0.018, *z* = 12.67, *P* = 8.01 × 10^−37^; African (AFR): *r*^2^ = 0.0086, *z* = 2.73, *P* = 6.33 × 10^−3^, admixed (MIX): *r*^2^ = 0.0069, *z* = 5.47, *P* = 4.41 × 10^−8^]. To further understand PVS changes across the life span, within ABCD (full sample) and UKB (subsample B), we visualize the effect of age and sex—shown in fig. S30. Despite the much smaller age range of ABCD versus UKB, we find a notably larger age effect in ABCD (*z* = 53.2, *r*^2^ = 0.16, *P* < 10^−100^) than in UKB (*z* = −4.00, *r*^2^ = 4.46 × 10^−4^, *P* = 7.29 × 10^−5^) with females appearing to be phase advanced in the adolescent age range (*z* = 27.0, *r*^2^ = 0.048, *P* < 10^−100^). In sum, these results demonstrate that the genetics underlying the Hemochromatosis Brain PVS are detectable both in early adolescence and in aging individuals and that there are notable age-related changes in early adolescence.

### Iron homeostasis in human brain and neurological disorders

**Fig. 3.**
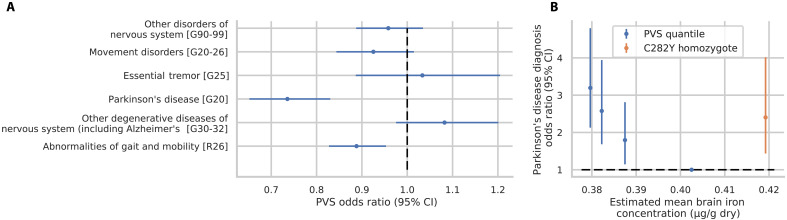
Logistic regression of PVS against neurological disorder diagnoses. Inverse probability weights (IPW) for each disorder can be found in table S6. (**A**) PVS OR predicting each neurological disorder. (**B**) PVS quantile weighted regression to predict PD in subsample C, each point represents a categorical factor indicating one of four PVS quantiles (blue) or C282Y homozygosity (orange). IPW of 3.89 was used for PD cases in imaging sample (blue)—see Materials and Methods. Regression (*y* axis) was performed using PVS from T2-weighted, and *x* axis is an estimate of mean brain iron concentration using T2* imaging for each group. Error bars for both plots indicate 95% confidence intervals (CI). The Pearson correlation of PVS with estimated mean brain iron concentration was 0.41.

After establishing the genetic determinants of the Hemochromatosis Brain, we next examined whether this measure was associated with any neurological disorders including movement disorders and abnormalities of gait. We found that the PVS in subsample B (containing no *C282Y* homozygotes) significantly predicted reduced risk for PD [odds ratio (OR) = 0.74, *Z* = −4.98, *P* = 6.42 × 10^−7^] and abnormalities of gait and mobility (OR = 0.89, *Z* = −3.28, *P* = 0.001)—see Fig. 3A and table S6. The associations were specific to movement and gait disorders, as we did not find evidence of a PVS association with diagnoses of any other neurological disorders tested. To gain greater insight into the PVS and PD risk association, we performed quantile weighted regression in subsample C using (i) each PVS quantile and (ii) *C282Y* homozygotes to predict PD status—see Materials and Methods. In addition, we estimated mean brain iron concentration for each PVS quartile and *C282Y* homozygotes using T2^*^ imaging. This enabled us to plot each group (PVS quartile or *C282Y* homozygote) according to their (i) estimated mean brain iron concentration and (ii) PD risk—with reference to the fourth PVS quartile. This analysis revealed (Fig. 3B and table S7) higher risk at the two ends of the estimated brain iron concentration spectrum: with the first PVS quantile (OR = 3.19, *Z* = 5.61, *P* = 2.06 × 10^−8^) and *C282Y* homozygotes (OR = 2.40, *Z* = 3.34, *P* = 8.41 × 10^−4^). This result underscores the critical role of iron homeostasis in maintaining the function of motor circuits. Note for Fig. 3B, although there appears to be a U-shaped relationship, a far greater proportion of individuals are present in the iron deplete range than in the iron overload range; each blue point represents a quantile (~25% of the cohort), while the orange point represents ~0.6% of the cohort. No such U-shaped relationship was observed for abnormalities of gait and mobility risk (fig. S31).

## DISCUSSION

Here, we have presented a continuum of iron homeostasis localized to motor circuits of the brain which is predictive of risk for movement disorders. We have shown that this dysregulation spectrum is strongly associated with genetic variation ($h_{\text{ldsc}}^{2}=0.381$), discovering both novel variants and variants proximal to known key regulators of iron homeostasis. Furthermore, the signal was enriched in gene expression patterns of glial cells—a cell group known to have highest concentrations of iron in the brain (*38*). In addition, MR analysis showed that iron markers in peripheral blood have a strong influence on brain iron accumulation differences. Yet, the PD risk profile exhibited a U shape, indicating that iron homeostasis is more relevant than absolute iron concentration. These results outline the genetic drivers, mediated through glial cells, influencing iron regulation in the brain and the putative consequences of disrupting this system. Together, these results indicate subgroups of PD that relate to the hormetic nature of iron in the brain, in which both depleted and excess levels can impart toxic effects, likely via different mechanisms (*4*, *40*, *41*).

The results from this analysis validate an established relationship between iron and movement disorders while providing a potential novel biomarker for PD risk. Previous postmortem and in vivo imaging studies have found higher iron deposition in substantia nigra, putamen, and red nucleus (*42*); these regions have small nonzero weights contributing to the Hemochromatosis Brain (fig. S2B). Nonetheless, R2* intensities in these regions are significantly correlated with the PVS (fig. S2C)—indicating that iron-related signal in these regions is being captured by the Hemochromatosis Brain through contributions from correlated regions. Nevertheless, it is worth noting that because of the small size of the substantia nigra and the large voxel size used in this study, coverage and partial volume effects may have limited the representation of this critical region. Of note, there is a paucity of in vivo imaging results of PD results reporting on the cerebellum (*42*)—a region which displays the largest contribution to the PVS and which has been shown to have nonsignificant lower iron levels in postmortem PD brain samples (*43*, *44*). We believe that, in addition to including the understudied region of the cerebellum, our PVS approach captures a central iron dysregulation spectrum which describes a misdistribution of iron that has proven difficult to detect using traditional approaches. In addition, deficits in iron have also been associated with movement disorders, with anemic patients and male blood donors being more likely to suffer from PD (*45*, *46*). Furthermore, a class of rare genetic mutations results in a disease called NBIA presents with a stereotyped pattern of iron accumulation in the basal ganglia similar to the univariate pattern observed in the Hemochromatosis Brain. The clinical presentation of this disorder is of dystonia and other Parkinsonism symptoms. Moreover, in the context of the MPTP model, which is the most extensively researched animal model of PD, it seems that ferroptosis, a form of cell death that relies on iron, has emerged as a key mechanism responsible for causing the motor symptoms associated with movement disorders (*4*). The work presented in the manuscript once again underscores the link between iron dysregulation in the brain and the risk of developing a movement disorder.

In addition, we find that, to a lesser degree, the PVS predicts risk for abnormalities of gait and mobility. This diagnosis represents a heterogeneous set of disorders. In contrast to International Classification of Diseases (ICD10): G20 for PD, ICD10: G26 represents symptom-based code and does not directly indicate a specific neurological or MRI finding. Given this heterogeneity, it is perhaps unexpected to find any coherent predictive signal from MRI. On the other hand, the PVS has large contributions from the basal ganglia and cerebellum, regions known to be heavily involved in gait (*47*, *48*). Furthermore, prior work has found differences in these regions using diffusion MRI (*49*) and midbrain volumetric differences (*50*) in patients with high-level gait disorder. It may prove useful to understand the degree to which our results and those from these previous studies, using other imaging modalities, are capturing overlapping underlying biological processes.

The polygenic signal we uncovered for the Hemochromatosis Brain revealed many known master regulators of iron homeostasis. In addition, we uncovered 13 novel loci (not implicated in any previous GWAS), some of which were linked to genes involved in metal recycling/transport and one locus proximal to *DENND4C*—a gene hypothesized to be implicated in neurodegeneration and PD (*31*). A question arises that if the Hemochromatosis Brain is so strongly related to iron regulation, why were we able to uncover 13 loci that were not previously reported in well-powered GWAS of peripheral iron markers (*30*)? First, these 13 loci may be specific to central iron regulation and the emergent pattern of iron accumulation observed regionally in the Hemochromatosis Brain. We identified two variants (*rs73210039* and *rs3817672*)—representing distinct loci—which appeared to show differential effects on systemic versus central iron levels. These variants were both proximal to *TFR1/TFRC*, a gene critical to cellular iron uptake. Further, LDSC analysis revealed a nonsignificant genetic correlation between the hemochromatosis brain and peripheral iron makers supporting the case for distinct genetic architectures of central and peripheral iron regulation. Second, the Hemochromatosis Brain may represent a more stable, long-term, and more heritable measure of iron in the body than what is detected through the blood. We observe a higher heritability for the Hemochromatosis Brain than peripheral blood markers, and measures of peripheral blood markers appear to be unstable over repeated measures (*51*). We hope that future work can further help disentangle the genetic contribution of central versus peripheral iron regulation.

Although epidemiological, imaging and postmortem studies suggest a link between PD and iron, and GWAS of the disorder has not uncovered a major contribution from iron (*52*). In our study, we only find one independent significant SNP (*rs13107325*, *P* = 3.66 × 10^−41^) overlapping with variants previously associated with PD. We also find that the Hemochromatosis Brain does not display any genetic correlation with the largest previous GWAS of PD (fig. S27). Our interpretation of this result is that iron dysregulation may represent one potential contributor to PD risk, and without this subtype being identified in current PD GWAS, these unmodeled heterogeneous genetic effects lead to null findings for iron-related variants. This idea of genetic heterogeneity is thought to explain the modest GWAS results of major depression (*53*), and we aim to investigate this possibility in future work. Much of the current PD results highlight lysosomal dysfunction as a principal mechanism underlying disease pathopysiology (*54*). The contribution of iron dysregulation to PD risk may still converge on lysosomal dysfunction. For example, iron depletion or overload leading to oxidative stress and cell death resulting ultimately in disease presentation; this process could be exacerbated in patients with preexisting lysosomal dysfunction. Beyond the genetic contribution to brain iron accumulation showcased in the current work, recent results following on from this analysis demonstrate the influence of dietary preferences jointly on central iron accumulation and PD risk (*55*), highlighting the importance of understanding environmental factors in PD etiology.

Iron chelators (drugs that facilitate the removal of iron from the body) have recently emerged as a potential therapeutic target for movement disorders with mixed outcomes. In the treatment of NBIA, iron chelation with deferiprone has shown promise in improving symptom progression (*15*, *17*). Conversely, a phase 2 randomized control trial of deferiprone in 372 patients with PD was stopped early due to worsening symptom progression from treatment (*16*). These results may be consistent with our findings if NBIA and PD represent two ends of the iron dysregulation spectrum. With this view, the more rare cases of NBIA result from iron overload thereby benefiting from iron chelation. This is in contrast to PD in the general population which may lie in the more iron-deplete range thereby being exacerbated by chelation therapy. In this interpretation, NBIA would align with the risk profile of *C282Y* homozygotes and patients with PD with the lower PVS quantiles in Fig. 3B. In support of this view, we see that the bulk of the UKB imaging sample lies in the iron-deplete range of PD risk with each of the four blue points of Fig. 3B representing ~25% of the sample and *C282Y* homozygotes representing 0.06%. This indicates that future iron-based interventions may benefit from MRI-based patient stratification, such as through the use of the Hemochromatosis Brain PVS, enabling targeted treatment.

Single-cell enrichment analysis implicated glial, and not neuronal, cells as the principal cell class associated with our Hemochromatosis Brain GWAS results. This is consistent with findings that glial cells, particularly oligodendrocytes, show larger concentrations of iron than neuronal cell types (*38*, *56*) and is also consistent with the role of glial cells’ in maintaining brain homeostasis (*57*). In net, it has been estimated that 80% of iron stores in the human brain are within glial cells (*58*). Although our results indicate that glial cells appear to mediate central disruption of iron homeostasis, the consequence of these disruptions may be borne by other cell types. Recent work has established that microglia are highly responsive to iron perturbations and are drivers of neuronal iron-dependent death (*59*). Furthermore, dopamine neurons, with a strong dependency on iron for dopamine synthesis (*40*) and mitochondrial function (*60*), may represent a cell class particularly sensitive to this disruption. In support of this view, studies show that dopaminergic neurons are the cell class to show largest iron concentration differences between healthy and PD brains (*58*, *61*). Further, despite glial cells being widespread across the brain, our analysis of tissue enrichment across the human body found our GWAS signal to be localized only to the substantia nigra and hippocampus. The former being rich in dopaminergic neurons and the primary site of PD pathology (*62*), and the latter known to show accelerated atrophy in PD (*63*). Together, cell- and tissue-specific enrichment analysis indicates that iron disruption of glial cells within the substantia nigra and hippocampus leads to variability in iron dysregulation as captured by the PVS. These results further support the view that the Hemochromatosis Brain represents an iron-specific endophenotype of relevance to PD.

Analysis revealed that a genetic signature of the Hemochromatosis Brain appears to be generalizable across a 50-year age span from adolescence (ABCD) to late adulthood (UKB). Within ABCD, we also observed a larger change in PVS scores as a function of age than for UKB (fig. S30)—this recapitulates previous findings of brain iron accumulation exhibiting an early exponential pattern with the largest increases observed in the first two decades of life (*11*). As others have suggested (*64*), this may be indicative of the development of the dopaminergic system, which relies heavily on iron. In addition, along this developmental trajectory, we find evidence that females appear to phase advanced when compared to males—a pattern which is observed for other markers during adolescence (*65*, *66*). It is possible that early-life differences in regional brain iron which we detect predispose individuals to different neurological risk profiles later in life.

An important limitation of the current study is its observational nature. Although T2-weighted MRI signal is known to be related to iron concentrations (*67*), and the GWAS signal we detect appears to implicate iron-related genes with encouragingly strong MR results, we cannot conclude that iron specifically is imparting a causal impact on disease risk for PD. It is possible that the iron-related signal we are picking up may be serving as a marker for another biological process that is responsible for generating the differential risk for PD. In addition, MRI is inherently unable to distinguish between bivalent and trivalent iron, which is of biological importance for a complete understanding of iron homeostasis. Further, the T2-weighted signal specifically is not solely reduced by higher iron concentrations; processes of edema and gliosis also affect T2-weighted intensity (*68*). Moreover, manganese similarly shortens the T2-weighted signal and is known to accumulate in the basal ganglia as a result of liver cirrhosis which is common with peripheral iron overload (*69*). It is possible that some of the effects captured by the PVS are related to these non-iron processes. Although R2* is linearly related to iron concentrations (*10*), we observe modest correlations of mean R2* intensities with the PVS (fig. S2C). It must be noted that we used R2* estimating equations that have been calibrated on biopsied liver tissue (*70*), where iron concentrations are substantially higher than in the brain. In addition, R2* MRI sequences of the liver typically collect a minimum of eight echoes (*70*) versus the two echoes collected as part of the brain R2* sequence in UKB. These issues likely affect the accuracy of iron estimation, via R2*, in the current study. Nevertheless, the convergent evidence of lead GWAS hits in iron regulators and strong MR results implicating peripheral iron levels as casual drivers make us confident that the PVS is, at least to a large degree, capturing a continuum of central iron dysregulation. Last, because of correlations between brain regions and the regularization process during training of our PVS, some brain regions flip signs (compare univariate statistics and posterior weights in Fig. 1 and fig. S2). The PVS approach’s strength and weakness lies in its ability to condense a complex signal into one instrumental variable, indicative of a continuum of brain iron dysregulation. Although, in general, higher PVS values indicate more iron accumulation in net, this does not ensure that iron levels in each individual brain region align with this direction. This work highlights that central iron dysregulation may not align with simplifying assumptions such as global iron overload or deletion; instead, dysregulation may indicate a misdistribution in which individuals exhibit greater and lower iron levels in different brain regions simultaneously. We hope that our novel approach can be coupled with traditional approaches to further our understanding of central iron dysregulation and its relationship with disease.

Here, we have showcased a continuum of neural iron dysregulation related to PD risk and which underscores the hormetic nature of this vital element. We have demonstrated that this continuum is strongly influenced by both genetic drivers and peripheral markers of iron. We hope that this dysregulation spectrum may be useful in patient stratification and in understanding heterogeneity in treatment outcomes. To this end, we provide weights and code to easily calculate PVS for suitable MRI scans within new datasets—see Materials and Methods. Our novel approach could be applied more broadly to other archetype learning scenarios, providing a framework for greater biological interpretation of multivariate measures.

## MATERIALS AND METHODS

### UKB sample

Genotypes, MRI scans, and demographic and clinical data were obtained from the UKB under accession number 27412, excluding 206 participants who withdrew their consent. All participants provided electronic signed informed consent, and the study was approved by the UKB Ethics and Governance Council. The recruitment period for participants was from 2006 to 2010, and participants had to be 40 to 69 years old during this period to be included in the sample. For the current study, we analyzed data from a total sample of 38,937 individuals (20,435 females) with a mean age of 64.3 years (SD 7.6 years) for analysis. This sample was made up of individuals who had qualified imaging (36,243 individuals) and/or who were *C282Y* homozygotes (2888 individuals). We split the sample of individuals with qualified imaging into two non-overlapping groups: subsample A for *C282Y* homozygote classifier training and subsample B for deploying this classifier, conducting GWAS on Hemochromatosis Brain and Disease Enrichment. A final group, subsample C, was used for weighted quantile regression (see below) which was subsample B with the addition of all 2888 *C282Y* homozygotes from the whole UKB sample. Table S1 summarizes demographics of these three subsamples and a description of which analyses they were used for. Genotype and health record data were collected from January 2006 to May 2021, and neuroimaging data were collected from April 2014 to May 2020. Data analysis was conducted from January 2022 to October 2022. This study follows the Strengthening the Reporting of Observational Studies in Epidemiology reporting guideline for cross-sectional studies (*71*).

### UKB image acquisition

In the current study, we used three imaging modalities: (a) T1-weighted (T1w) scans for image registration, (b) T2-weighted were collected as the *b* = 0 s/mm^2^ reference scan for diffusion weighted scans, and (c) T2* scans as part of the susceptibility weighting sequence. Voxel-wise estimates from (b) were used as the primary imaging modality for analysis in the paper (*C282Y* classifier training and for generating PVS; see below) as previous work demonstrated this modality (T2 weighted) had larger effects for *C282Y* homozygosity than T2* (*25*). Voxel-wise estimates from (c) were only used to estimate mean iron concentration estimates, from R2* = 1/T2*, in Fig. 3B and fig. S2C. The motivation for using R2* for iron concentration estimation was the following: Although both R2 (1/T2) and R2* have been shown to be strongly correlated with iron concentrations, R2* specifically exhibits a linear relationship with iron concentration (*10*) making estimation more tractable. MRI scans were collected from three identically configured Siemens Skyra 3T scanners, with 32-channel receiver head coils, at three scanning sites throughout the United Kingdom. For diffusion scans, multiple scans with no diffusion gradient were collected (*b* = 0 s/mm^2^) to fit diffusion models. The average of these *b* = 0 s/mm^2^ scans was used as voxel-wise measures of T2-weighted intensities. Diffusion-weighted scans were collected using a SE-EPI (Spin Echo–Echo Planar Imaging) sequence at 2-mm isotropic resolution. T1w scans were collected using a three-dimensional (3D) MPRAGE (Magnetization Prepared Rapid Gradient Echo) sequence at 1-mm isotropic resolution. Voxel-wise T2* values were estimated as part of the susceptibility-weighted imaging protocol at a voxel resolution of 0.8 mm × 0.8 mm × 3 mm and with two echoes (echo times, 9.42 and 20 ms). To reduce noise, T2* images were spatially filtered (3 × 3 × 1 median filtering followed by limited dilation to fill missing data holes). If a person had multiple (longitudinal) scans, then we used the first scan. Further details of image acquisition can be found in (*72*).

### ABCD sample

The ABCD study is a longitudinal study across 21 data acquisition sites following 11,878 children starting at 8 and 10 years old. This paper analyzed the baseline and 2-year follow-up sample from data release 4.0 [National Institute of Mental Health Data Archive (NDA) DOI: 10.15154/1523041]. The ABCD study used school-based recruitment strategies to create a population-based, demographically diverse sample with heterogeneous ancestry. Genotype data were imputed using the TOPMED (Trans-Omic for Precision Medicine) imputation server (*73*–*75*), and genetic principal components were estimated using PC-AiR (Principal Components Analysis in Related Samples) (*76*) as described elsewhere (*77*). We selected individuals who had passed neuroimaging and genetic quality control checks. We calculated participants’ continental genetic ancestry as calculated using SNPweights (*78*) and aligning with 1K Genomes Project (*79*) and indigenous reference panels (*80*). Each individual was assigned an ancestry proportion to four continental groups: European (EUR), AFR, Native American, and South Asian or East Asian. From this, participants were categorized into one of the three largest ancestry groups of European (EUR), AFR, and MIX. Individuals were categorized as EUR or AFR if they exceeded 80% ancestry within one of these continental ancestry groups and MIX if they were less than 80% ancestry across all groups. This resulted in 5977 EUR, 687 AFR, and 3135 MIX individuals for performing ancestry-stratified analysis—see table S2 for demographic details.

### ABCD image acquisition

T1w and diffusion-weighted MRI (dMRI) scans were collected using Siemens Prisma and Prisma Fit, GE Discovery 750, Phillips Achieva, and Ingenia 3T scanners. Scanning protocols were harmonized across 21 acquisition sites. Full details of harmonization routines have been described elsewhere (*81*, *82*). T1w images were acquired using a 3D MPRAGE scan at 1-mm isotropic resolution. dMRI scans were acquired in the axial plane at 1.7-mm isotropic resolution, with seven *b* = 0 s/mm^2^ frames. The average of *b* = 0 s/mm^2^ scans was taken as voxel-wise measures of T2-weighted intensities.

### Image preprocessing

Scans were corrected for nonlinear transformations provided by MRI scanner manufacturers (*83*, *84*). T2-weighted and T2* scans (for UKB) were registered to T1w images using mutual information (*85*). Intensity inhomogeneity correction was performed by applying smoothly varying, estimated B1-bias fields (*82*). Images were rigidly registered and resampled into alignment with a preexisting, in-house, averaged reference brain with 1.0-mm isotropic resolution (*82*). Atlases used to define regions of interest for classifier feature importance analysis are shown in table S3.

### C282Y homozygote classifier training and PVS generation

We aimed to train a classifier to predict *C282Y* homozygosity status from T2-weighted intensities. For training, we generated a sample derived from the 193 *C282Y* homozygotes with qualified imaging and found covariate matched controls at a ratio of 4:1 (cases to controls). Controls were matched for sex, age, scanner, and top 10 principal components of genetic ancestry. This was performed as described elsewhere with the exception that controls were selected as those with no *C282Y* mutations (i.e., *C282Y* heterozygotes were excluded from being cases or controls). This resulted in a subsample of 193 *C282Y* “cases” (112 female) and 767 “controls” (463 female), i.e., no *C282Y* mutations; for training the classifier. We refer to this sample as subsample A —see table S1 for description. For fitting, hyperparameter tuning, and evaluating the classifier, we used a fivefold cross-validation scheme in subsample A.

The *C282Y* homozygote classifier was fitted using a PVS framework (*26*) as follows. Let **X**^train^ represent the preresidualized and masked imaging matrix with *N* rows of individuals and *M* columns of voxels in the training sample. Each element of this matrix, $x_{i,j}^{\text{train}}$ represents the voxel intensity for the ith individual at the jth voxel for a T2-weighted scan. Here, each column in **X**^train^ was quantile transformed to a normal distribution and preresidualized for covariates of age, sex, MRI scanner, and top 10 principal components of genetics. Let **y**^train^ represent the 1D vector such that

$$y^{\mathrm{train}}=\left\{\begin{array}{c} 1,\;\text{if}\;\text{C282Y}\;\text{homozygote}\\ 0,\;\text{otherwise} \end{array}\right.$$

We then regress each column of **X**^train^ with **y**^train^ to generate a vector of univariate *z*-statistics, ***z***, of dimension *M*—where each element describes the univariate association of that voxel with C282Y homozygosity status. We then threshold this ***z*** to restrict to nominally significant voxels in which *P* < 0.01. Next, we could use the vector ***z*** to generate out of sample PVS’s from a test sample’s imaging data, to estimate liability of individuals along the Hemochromatosis Brain liability scale as follows: ${\hat{y}}^{\text{test}}={\mathrm{zX}}^{\text{test}}$ (these univariate statistics are displayed in Fig. 1). However, because of the correlation structure across voxels, this gives suboptimal out of sample prediction. As such, we use the correlation matrix of **X**^train^, **R**, to reweight ***z*** to obtain posterior effect sizes. **R** can be decomposed using singular value decomposition **R** *=* **USU**^*T*^(**U**, unitary matrix; **S**, diagonal matrix with singular values on its diagonal). We consider the regularized form of **R** as: **R***_r_* *=* **US***_r_***U**^*T*^, where **S***_r_* is obtained from **S** by keeping *r* largest singular values and replacing the remaining with *r*th largest. We then decorrelate association statistics to generate PVS classifier weights (posterior effects in Fig. 1) as $w_{r}=R_{r}^{-1}z$ which were in turn used to generate PVS scores for each individual as: ${\hat{y}}_{r}^{\text{test}}=w_{r}X^{\text{test}}$. During cross-validation in subsample A, nine ${\hat{y}}_{r}^{\text{test}}$ were generated with different values of [1, 5, 10, 20, 50, 100, 200, 500, 1000, and *M* (i.e., no regularization)]. The *r* value that maximized the association between **y**^test^ and ${\hat{y}}_{r}^{\text{test}}$ (*r* = 13) was selected for displaying the receiver operator characteristic curve in Fig. 1. For downstream analysis in subsample B, we used the mean optimal value, *r* = 13, to fit posterior weights across the whole of subsample A. ${\hat{y}}_{r}^{\text{test}}$generated in subsample B is what we refer to as the Hemochromatosis Brain PVS.

To capture feature importance of the classifier and univariate weights for different brain regions (Fig. 1 and fig. S2), we normalized ***z*** and **w***_r_* to be of unit length as $\tilde{z}$ and ${\tilde{w}}_{r}$. We then calculated the sum of squares of $\tilde{z}$ and ${\tilde{w}}_{r}$ for each voxel that fell within a given brain region (see table S3 for atlases used to define brain regions). For plotting results of weighted quantile regression (Fig. 3B), we estimated that mean iron concentration was calculated for each individual by taking the mean across *P* < 0.01 voxels from T2* imaging (*70*). We then used previously published estimates linking R2* (1/T2*) values to iron concentration as [μg/g dry] = *C* × R2 × [Hz]/3.2, where *C* = 2000/36.

### GWAS of Hemochromatosis Brain

To understand the genetic determinants of the iron accumulation captured by the hemochromatosis brain, we performed a GWAS of the PVS generated in subsample B (non-overlapping with subsample A used for classifier training)—see table S1. While performing this GWAS, we covaried for age, sex, scanner, and top 10 components of genetic ancestry. We restricted this analysis to individuals declared as self-identified “white British” and similar genetic ancestry (using Data Field: 22006), and this resulted in 30,709 individuals. Four thousand six hundred eight remaining individuals from subsample B were used for replication of GWAS discoveries. In addition, to enable the calculation of genetic correlations of related traits in UKB, we performed GWAS for intracranial volume, as a measure of brain size, and four red blood cell traits: mean corpuscular volume, mean corpuscular hemoglobin, mean sphere cell volume, and hemoglobin concentration. If there were multiple instances of a variable, we took their mean value for each subject. For these measures, as well as the PVS, we quantile transformed them to a normal distribution to enforce normality and reduce leverage of outliers. We used sample and variant quality controls of --geno 0.05, --hwe 1e-12, --maf 0.005, and --mind 0.1 using PLINK (v2.00a3.6LM) (*86*). This resulted in 7,131,446 remaining variants, and no individuals were removed. PLINK was used for performing GWAS across these six traits (using –glm). For discovered variants, we performed validation in 4608 individuals who self-identified in any other category as white British by performing the same process for GWAS discovery. Using independent significant SNPs (see the locus definition below) from the discovery set, we evaluated replication in two ways: (i) compared/correlated coefficients and (ii) evaluated sign concordance of coefficients between discovery and replication cohorts. For (ii), we used a binomial test to assess the significance of in proportion of sign concordant SNPs.

### Gene burden analysis using whole-exome data

To test whether rare genetic variants were associated with variability in brain iron levels captured by the Hemochromatosis Brain PVS, we performed gene burden analysis using Reginie (v3.1.1) (*87*). For this analysis, we had 19,498 individuals from subsample B with available genetic data. Using annotations from SnpEff (*88*) (v5.0) of each observed mutation, we applied a mask of mutations labeled as high or moderate consequence. Using parameters of --aaf-bins 0.1,0.05,0.01 and --bsize 200, and the same covariates described above, we associated each binary gene burden score with variability in PVS. Across 18,860 genes tested, we defined discoveries as those whose *P* value exceeded Bonferroni significance of 0.05/18,860.

### GWAS locus definition and discovery overlap GWAS catalog

Summary statistics were uploaded to FUMA (FUMA: v1.4.1, MAMGA v1.08) (*36*) for locus definition and gene mapping. “Independent significant SNPs” represent genome-wide significant SNPs that are in moderate to low linkage disequilibrium (LD) with one another (*r*_LD_ < 0.6). “Genome-wide loci” represent regions in which independent significant SNPs are merged if they are (i) in moderate LD with one another (*r*_LD_ > 0.1) or (ii) are physically close to one another (<250 kb). FUMA provides an output of independent significant SNPs and reports which of these overlap with previous studies reported in GWAS catalog (e104_r2021-09-15). We defined novel loci as those which did not contain any independent significant SNPs overlapping with associations in GWAS catalog. For SNPs that did overlap with previous GWAS catalog studies, we grouped these previously reported traits into broad categories defined in table S4. We allowed each significant SNP to be counted for multiple categories if it appeared in different studies across categories. However, we ensured that it was not counted more than once within a specific category, even if it appeared in multiple studies within that category. The results of this analysis are shown in fig. S26 and extended data tables.

### Mendelian randomization

MR analysis has become a powerful tool over the past two decades for determining causality from observational GWAS results (*89*). While randomized control trials are considered the gold standard for establishing causality by randomly assigning individuals to different exposure groups, MR uses the natural random assignment of genetic variants that occurs during meiosis. This method allows researchers to infer a causal relationship between an exposure and an outcome based on genetic data. To assess the causal strength of association between peripheral blood markers, PD and brain iron dysregulation as measured by the Hemochromatosis Brain was conducted MR using GSMR (Generalized Summary-data-based Mendelian Randomization) (*39*). To conduct these analyses, we used previous GWAS summary statistics of serum iron, transferrin saturation, total iron binding capacity, ferritin (*30*), and PD (*52*). LD was estimated from the 30,709 UKB individuals used for the PVS GWAS; this LD was then used to find independent significant SNPs to use as instruments for GSMR. The following parameters were used: gwas_thresh = 5 × 10^−8^, single_snp_hedi_thresh = 0.01, multi_snps_hedi_thresh = 0.01, heidi_outlier_flag = T, ld_r2_thresh = 0.05, ld_fdr_thresh = 0.05. Bivariate GSMR was run (in both directions) to confirm the directionality of causation, using the following exposures and outcomes:

1) Exposures: serum iron, transferrin saturation, total iron binding capacity, and ferritin. Outcome: PVS.

2) Exposures: serum iron, transferrin saturation, total iron binding capacity, ferritin, and PVS. Outcome: PD.

### Genetic correlation

LDSC (*34*) was used to estimate from summary statistics using the 1K genomes European ancestry reference panel. LDSC was also used to calculate genetic correlations with GWAS of intracranial volume and four red blood cell traits (mean corpuscular hemoglobin, mean corpuscular volume, mean sphered cell volume, and hemoglobin concentration) that we conducted in subsample B, as well as publicly available summary statistics of four blood iron traits (*30*), PD (*52*), and educational attainment (*90*).

### Single-cell enrichment

We performed cell type enrichment using the FUMA platform (*36*). Association statistics at the gene level were calculated using MAGMA (v1.08) (*91*). This gene-based distribution was then associated with expression patterns of 25 cell types from PsychENCODE single-cell RNA sequencing data collected from the adult brain (*37*) by performing a gene-property analysis (*92*).

### Tissue-specific enrichment

We used S-LDSC (*93*) to examine tissue type–specific enrichment of the Hemochromatosis Brain GWAS results. We analyzed annotations derived from 489 tissue-specific chromatin assays of bulk tissue from peaks for six epigenetic marks. These data were collected as part of the Roadmap Epigenomics and ENCODE projects (*94*, *95*), and annotations were downloaded from previous analysis (see URLs) (*35*). We controlled for baseline annotations as recommended by S-LDSC (*93*). We reported the signed enrichment *Z* statistics, as well as the corresponding false discovery rate (FDR)–corrected multiple comparisons adjusted *P* values.

### Polygenic/PVS validation

We tested the generalization of both our genetic and neuroimaging findings of the Hemochromatosis Brain to individuals from the ABCD cohort: a cohort, on average, more than 50 years younger than the UKB cohort from which these findings were discovered. Within the ABCD sample, we generate a PVS score for each individual at each available time point. This included 9799 individuals at baseline (age, 8.9 to 11.1 years) and 4615 at 2-year follow-up (age, 10.58 to 13.67 years). To generate PGSs, we calculated posterior effect sizes using PRScs (Polygenic Risk Score Continuous Shrinkage) (*96*) using the LD estimated from the 1K Genome European ancestry reference panel (*N* = 503). PGSs were then calculated by applying these posterior effect sizes using “plink –score” (v2.00) to ABCD genetic data. Performing ancestry-stratified analysis (within the groups described above), we fitted linear mixed effect models to predict the PVS of each individual with fixed effects of sex, age, top 10 principal components of genetic ancestry, and a categorical variable indicating the MRI software and serial number. We included random intercepts for subject ID (to account for longitudinal measures) nested with family ID membership. Variance explained was computed as $r^{2}=\frac{t^{2}}{t^{2}+\text{df}}$, where df is the degrees of freedom.

### Disease enrichment of Hemochromatosis Brain

We wanted to test the utility and specificity of the Hemochromatosis Brain PVS in distinguishing cases and controls of PD and other disorders of the brain. We performed this analysis in subsample B—see table S1. This resulted in 35,283 individuals (18,278 females, 0 *C282Y* homozygotes). For this sample, we generated a PVS for each individual from their imaging data using the *C282Y* homozygote classifier, as described above, trained in the full subsample A (regularization parameter set to *r* = 13 as the mean optimal value across cross-validation). This PVS is a single score per individual that, in essence, quantifies the degree of iron dysregulation in motor circuits observed in *C282Y* homozygotes. We then associated this Hemochromatosis Brain PVS with six disorders/classes of disorders of the brain using weighted regression logistic models with covariates of age, sex, scanning site, and top 10 principal components of genetic ancestry. These six different disorders/categories were as follows: other disorders of the nervous system (ICD10: G90-99), movement disorders (ICD10: G20-26), PD (ICD10: G20), essential tremor (ICD10: G25), other degenerative diseases of the nervous system (including Alzheimer’s) (ICD10: G30-32), and abnormalities of gait and mobility (ICD10: R26). These diagnoses were extracted from UKB field 41270. We performed weighted regression using inverse probability weighting (IPW) (*97*) to account for the depleted number of cases of each of these six disorders in the imaging subsample versus the whole UKB sample. That is, if the neuroimaging sample had threefold lower prevalence for a disorder when compared with the whole sample, we weighted cases with an IPW of 3 and controls with a weight of 1. This enabled regression across individuals in the imaging and non-imaging samples (see the section below) and estimates closer to population prevalence for these disorders. IPW values for each disorder are shown in table S6.

### Quantile weighted regression

Individuals were assigned to one of four quantiles (1 to 4) according to their PVS with first being the lowest 25% of PVS, second being scores between 25th and 50th percentiles, and so on. We aimed to quantify the PD risk of each of these quantiles with reference to *C282Y* homozygotes taken from the whole UKB sample, including those that did not have neuroimaging (2888 homozygotes taken from 488,288 individuals). To accomplish this, we again performed weighted regression using an IPW of 3.89 for PD cases in the imaging sample and 1 for all other observations. A model was then fit, within sample C of table S1 to predict PD from these quantiles and *C282Y* homozygote status as a categorical variable covarying for the same covariates described above. We then used estimated mean iron concentrations for PVS brain regions, as described above, to display the mean of this value for each group as shown on the *x* axis of Fig. 3B.

## Supplementary Materials

### The PDF file includes:

Supplementary Note Figs. S1 to S31 Tables S1 to S7 Legend for data S1 References
