# Supplementary material for: Hemochromatosis neural archetype reveals iron disruption in motor circuits
Source: Sci Adv. 2024 Nov 22;10(47):eadp4431. doi: 10.1126/sciadv.adp4431 (PMC11584016; doi:10.1126/sciadv.adp4431)
Supplement: Supplementary file 1 — Supplementary Note Figs. S1 to S31 Tables S1 to S7 Legend for data S1 References [file sciadv.adp4431_sm.pdf]

Science Advances
AAAS

Supplementary Materials for

Hemochromatosis neural archetype reveals iron disruption in motor circuits

Robert Loughnan *et al.*

Corresponding author: Robert Loughnan, rloughna@ucsd.edu

Sci. Adv. **10**, eadp4431 (2024)
DOI: 10.1126/sciadv.adp4431

The PDF file includes:

Supplementary Note
Figs. S1 to S31
Tables S1 to S7
Legend for data S1
References

Other Supplementary Material for this manuscript includes the following:

Data S1

Supplementary Note:

Potential Spurious Locus

We identified in total 43 genomic loci associated with the Hemochromatosis Brain PVS. However, one of these loci displayed a suspicious association pattern with nearby SNPs in LD - see Supplementary Figure 15 B - and such was removed from the final loci count.

Supplementary Figures

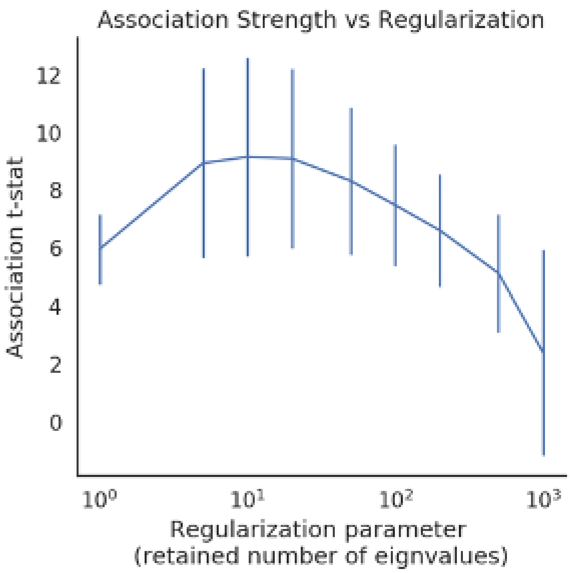

Supplementary Figure 1 ***Association strength (t-stat) between “Hemochromatosis Brain” Classifier and C282Y homozygote status (y-axis) and regularization parameter (x-axis)*** *– number of retained eigenvalues for SVD truncation (see methods). Points indicate association strength in test folds of 5-fold cross validation in Subsample B. r=13 was used for PVS generation in subsample B. Error bars indicate standard deviation in t-statistic across 5 folds.*

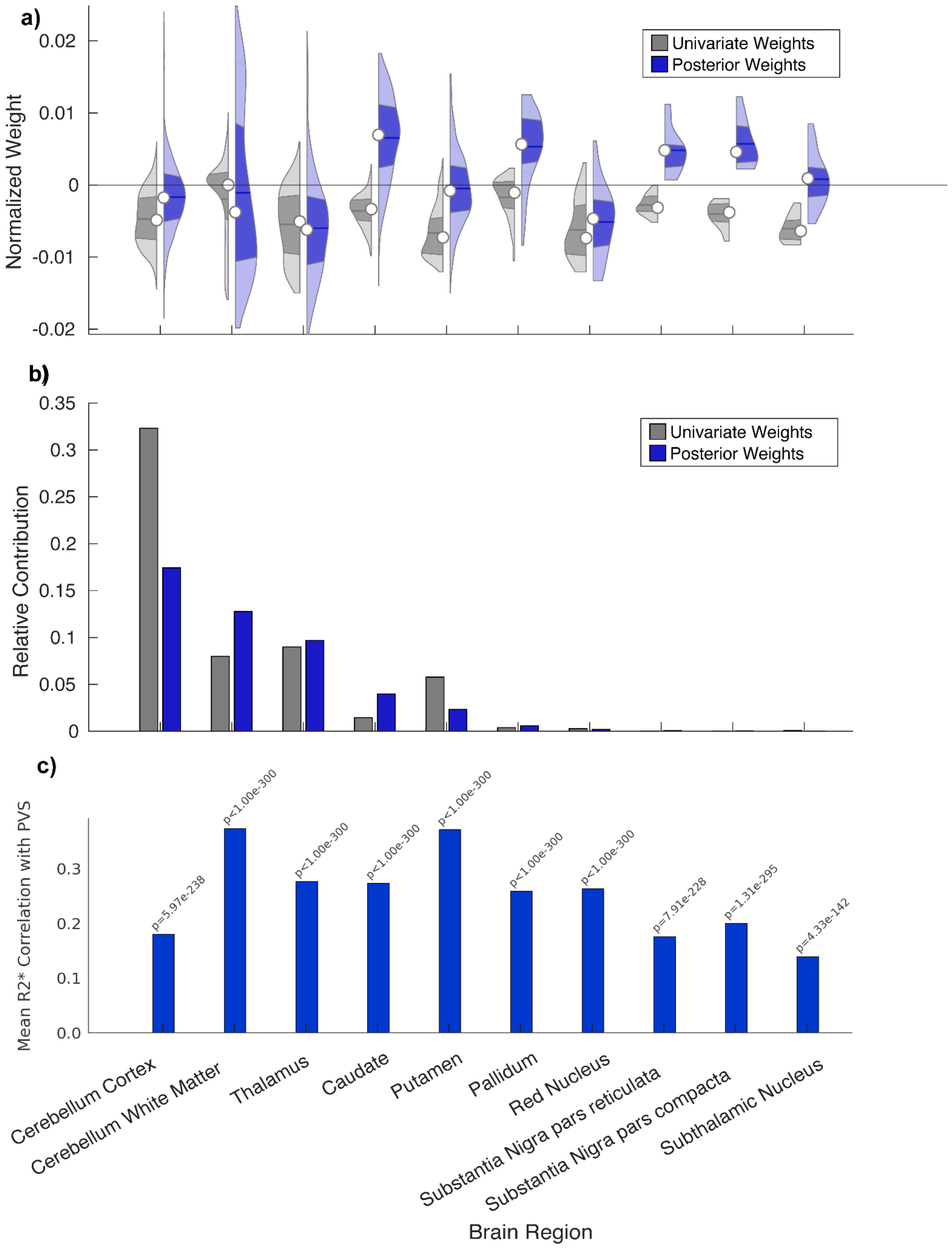

Supplementary Figure 2: ***PolyVoxel Score (PVS) weight breakdown by each region of interest (ROI)****. Panel a) indicates distribution non zero PVS weights for each region, where univariate statistics and posterior weight*

vectors are normalized to be of unit length. Panel b) indicates the relative contribution to each region as the sum of squares within each brain region for univariate statistics and posterior weights. Note: univariate weights are shown for reference however posterior weights were used for PVS generation. Panel c) indicates pearson correlation and p values (annotated) of Mean R2 Intensity values with PVS (derived from T2-weighted imaging) for each region. Correlations were calculated in Subsample B. Mean R2* values were calculated for voxels which had non-zero weights in Hemochromatosis Brain classifier.*

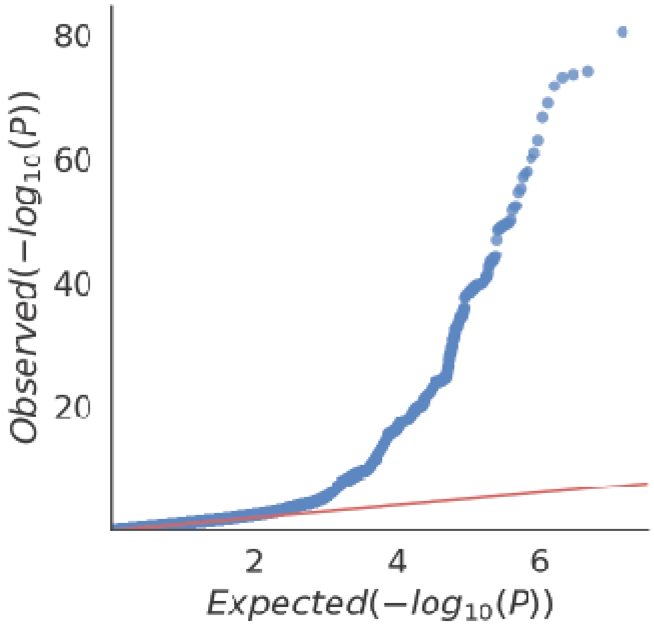

Supplementary Figure 3: ***QQ plot of GWAS of PVS****. Analysis conducted in European ancestry individuals within UKB subsample B (30,709 individuals).*

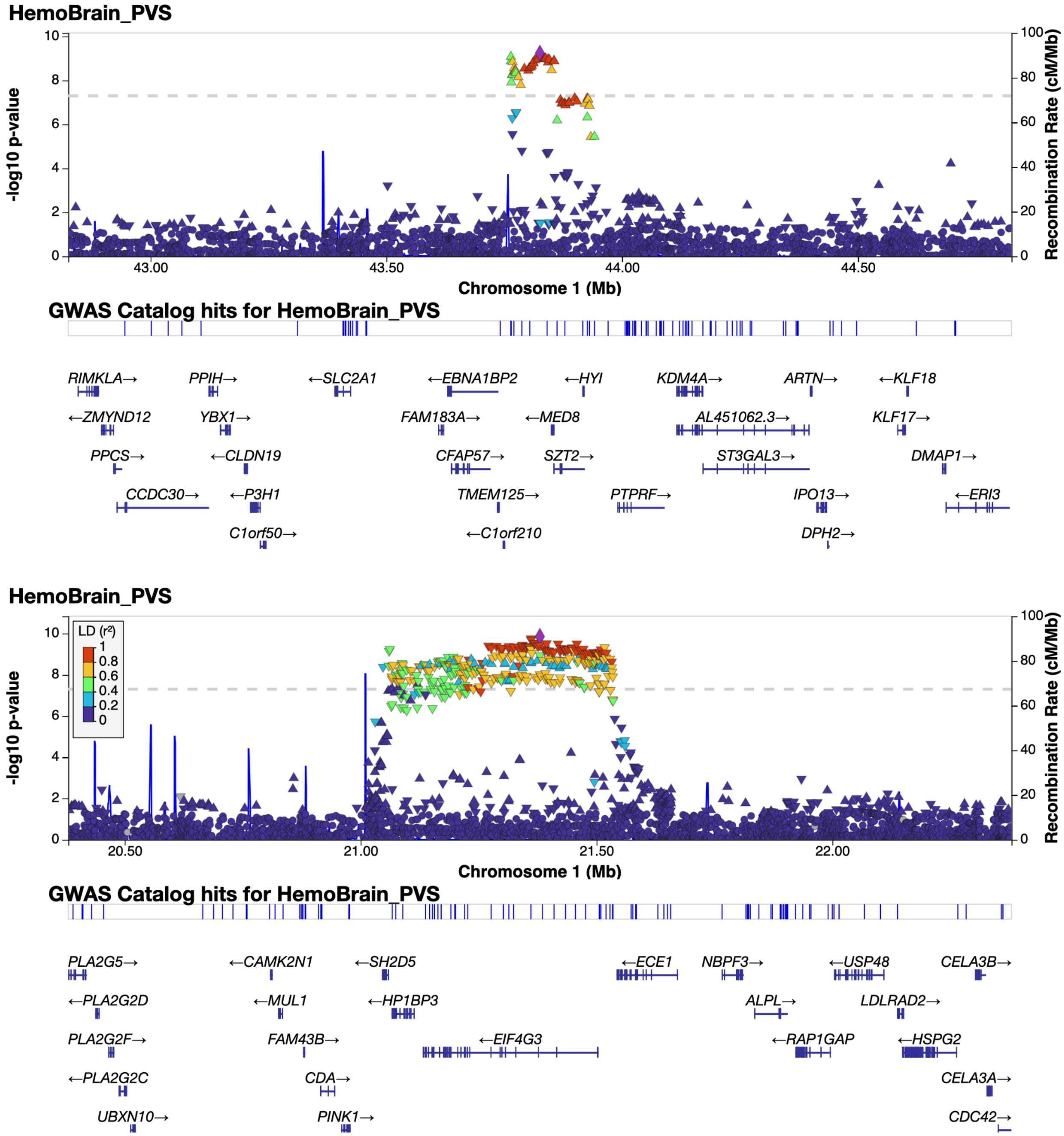

Supplementary Figure 4 ***LocusZoom(98) plot*** *A) for genomic locus 1. B) for genomic locus 2. See extended data tables for genomic locus numbering.*

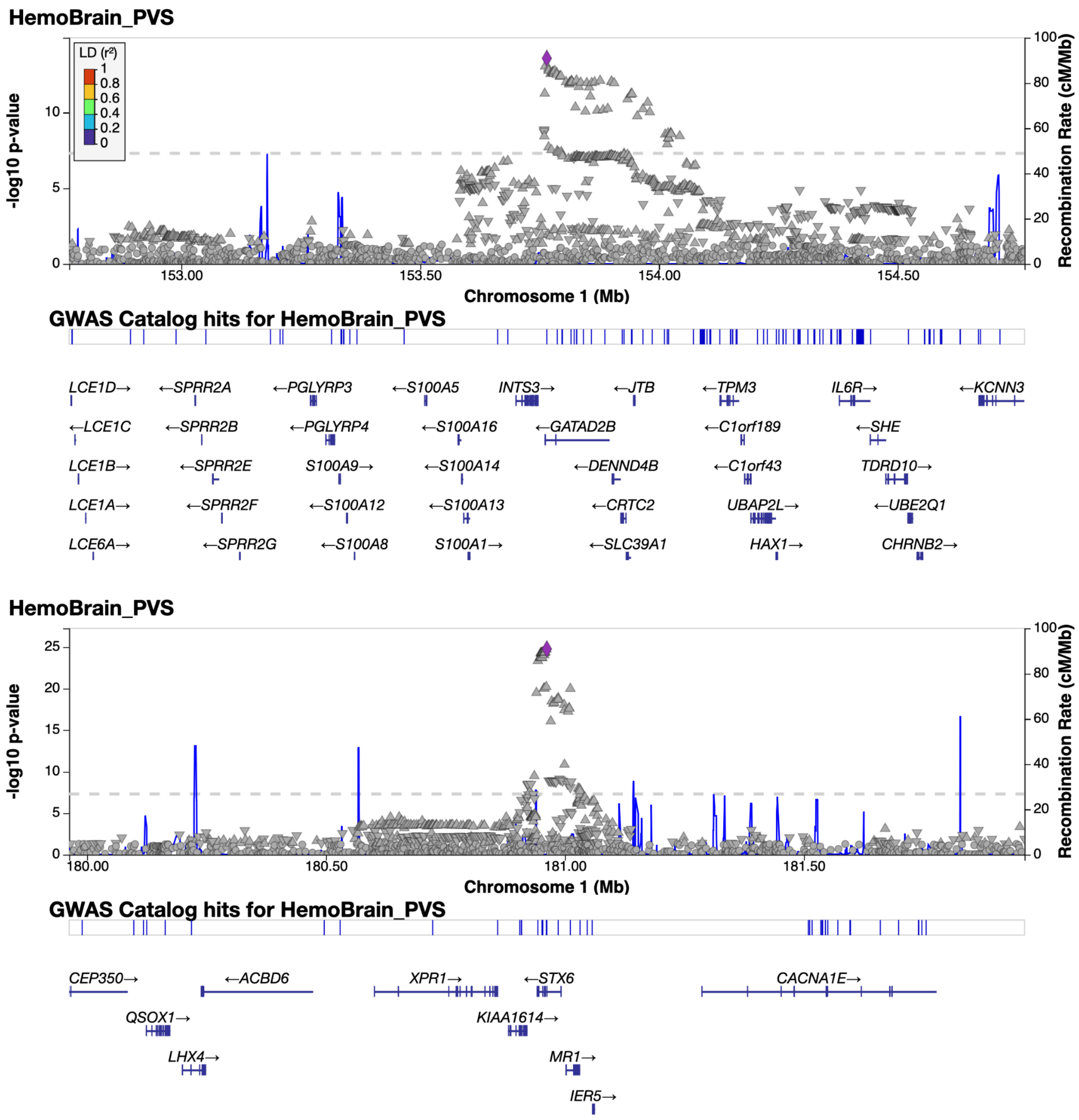

Supplementary Figure 5 ***LocusZoom(98) plot*** *A) for genomic locus 3. B) for genomic locus 4. See extended data tables for genomic locus numbering. Non-lead SNP points are in gray as lead SNP is not in the LD reference panel (1k genomes).*

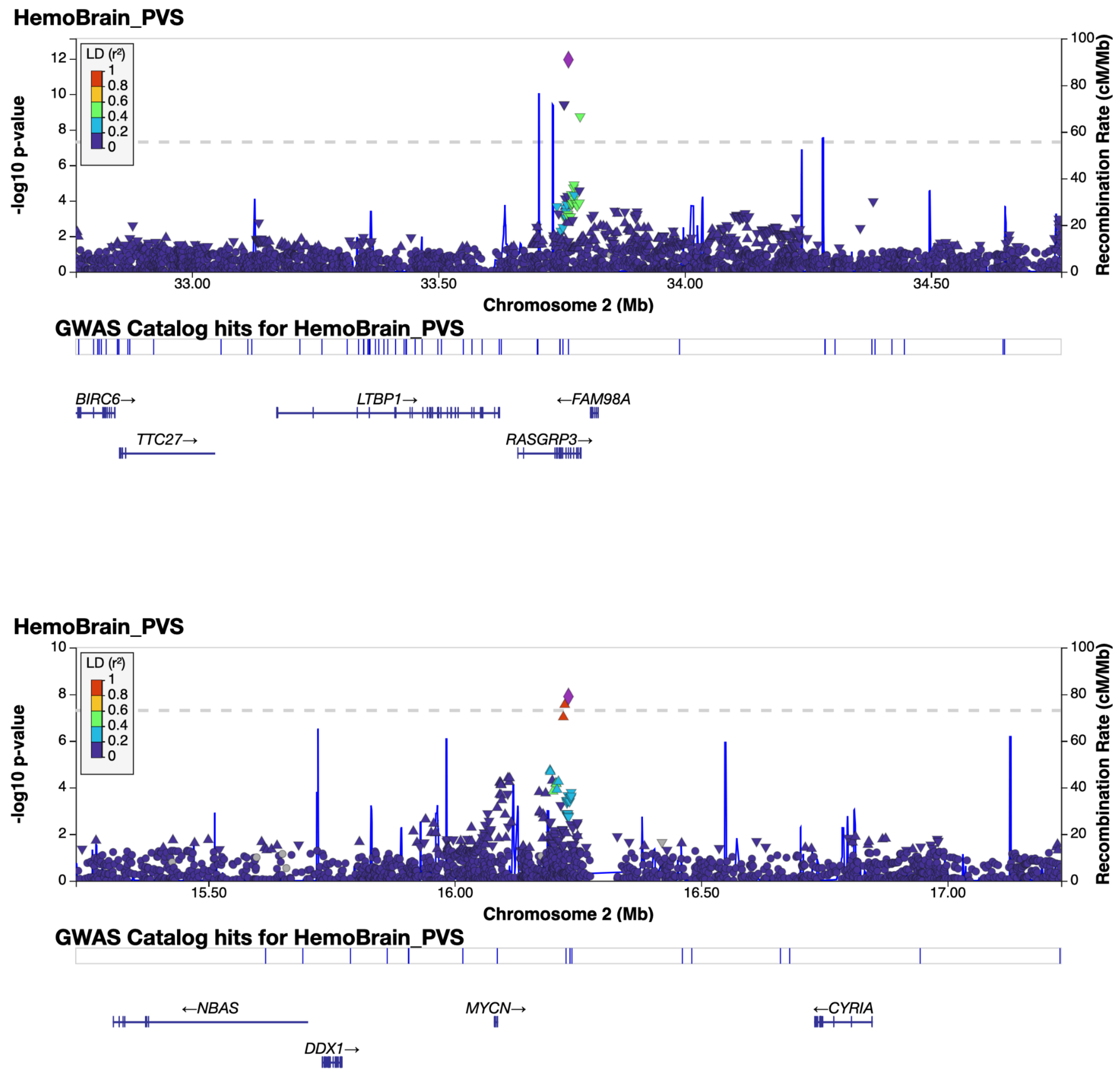

Supplementary Figure 6 ***LocusZoom(98) plot*** *A) for genomic locus 5. B) for genomic locus 6. See extended data tables for genomic locus numbering.*

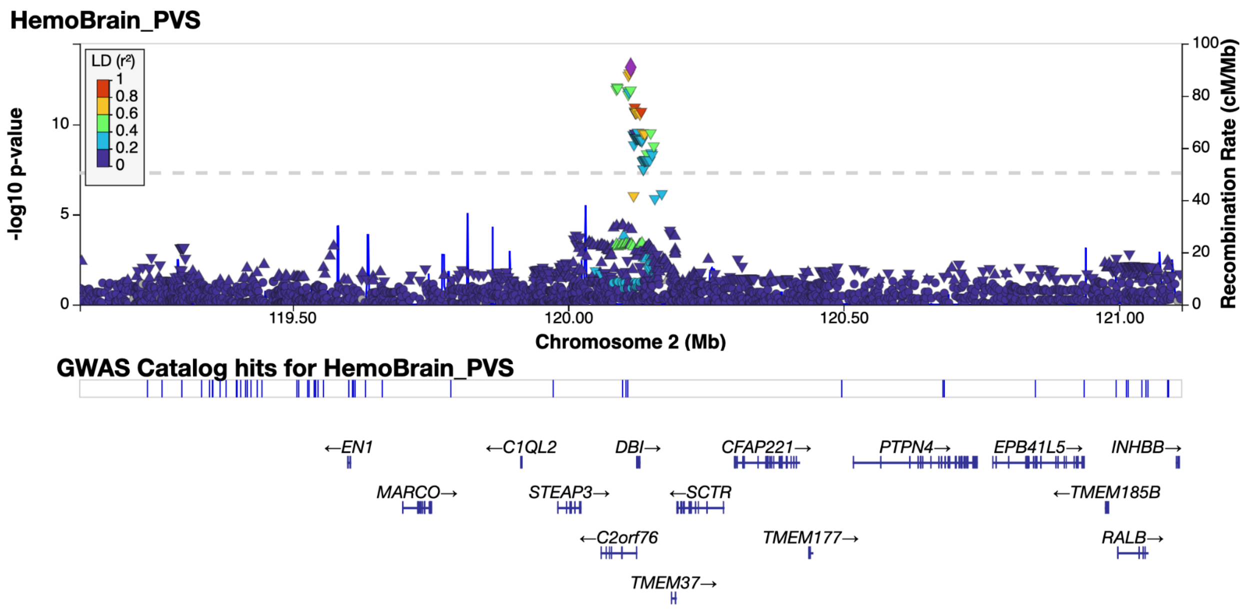

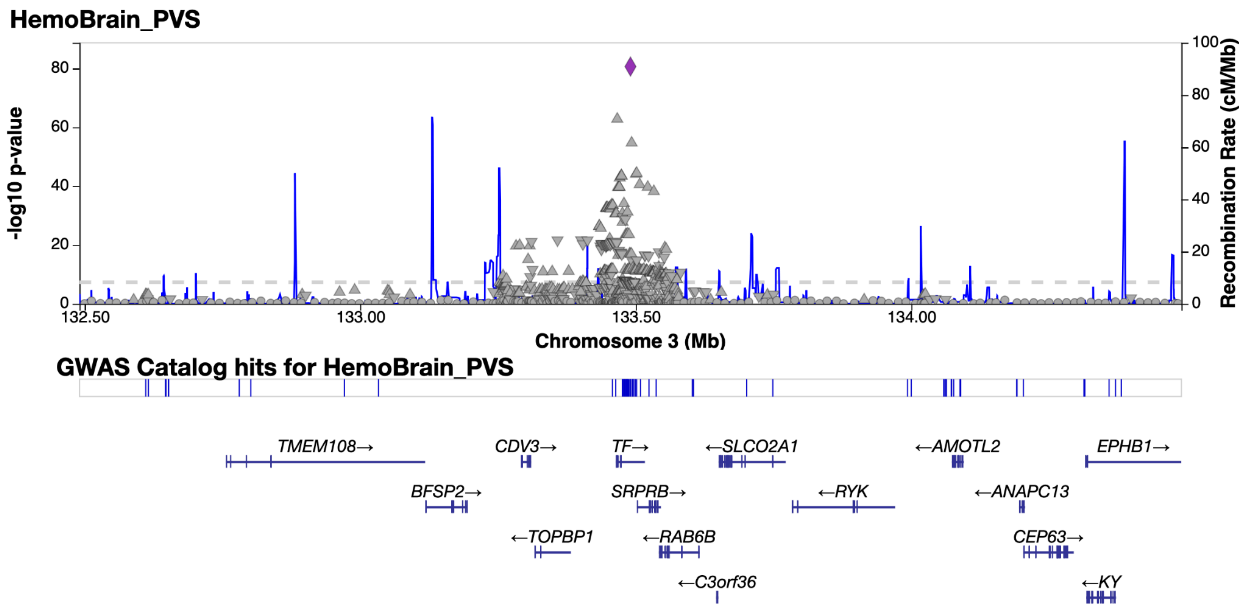

Supplementary Figure 7 ***LocusZoom(98) plot*** *A) for genomic locus 7. B) for genomic locus 8. See extended data tables for genomic locus numbering. Non-lead SNP points are in gray as lead SNP is not in LD reference panel (1k genomes).*

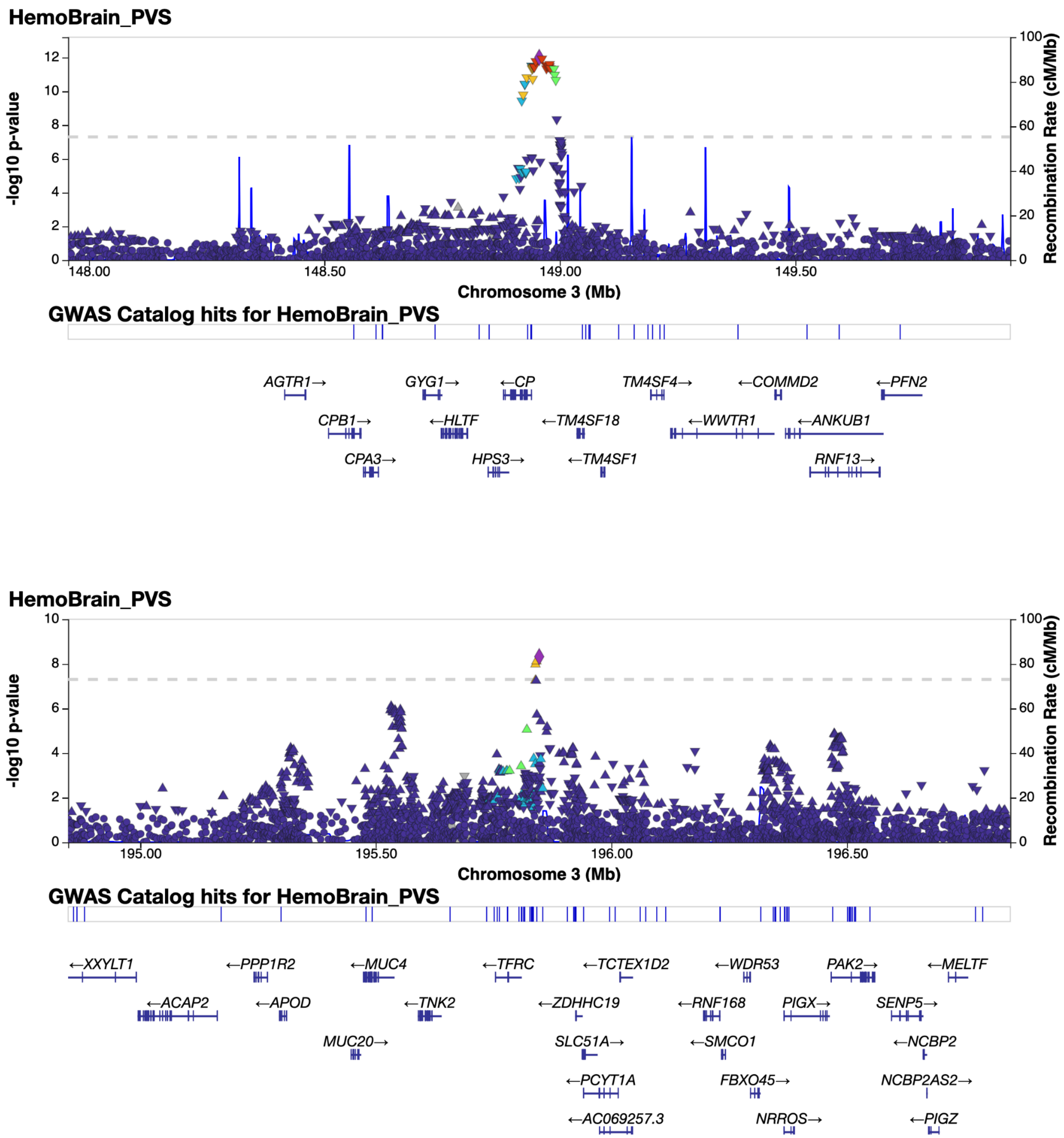

Supplementary Figure 8 ***LocusZoom(98) plot*** *A) for genomic locus 9. B) for genomic locus 10. See extended data tables for genomic locus numbering.*

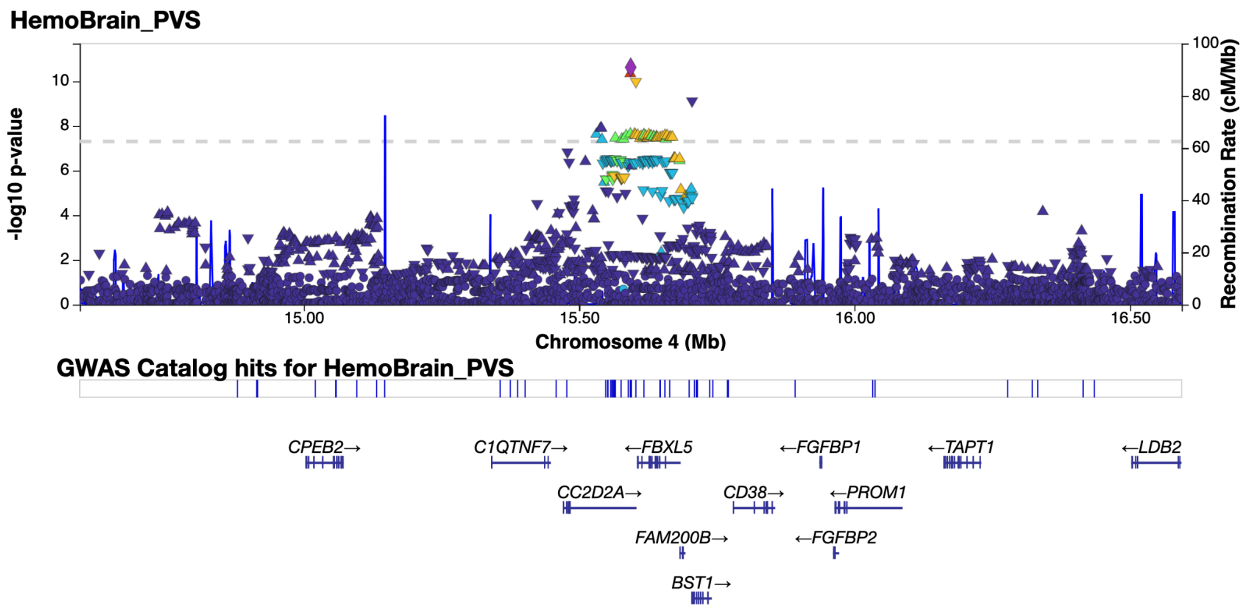

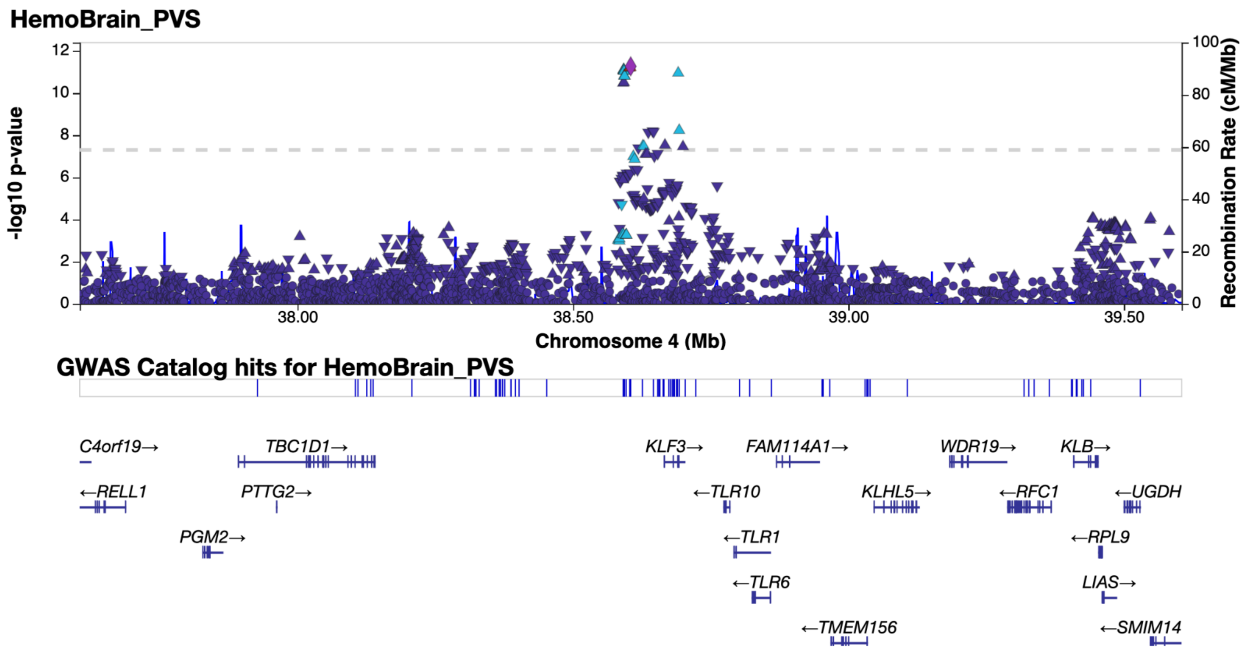

Supplementary Figure 9 ***LocusZoom(98) plot*** *A) for genomic locus 11. B) for genomic locus 12. See extended data tables for genomic locus numbering.*

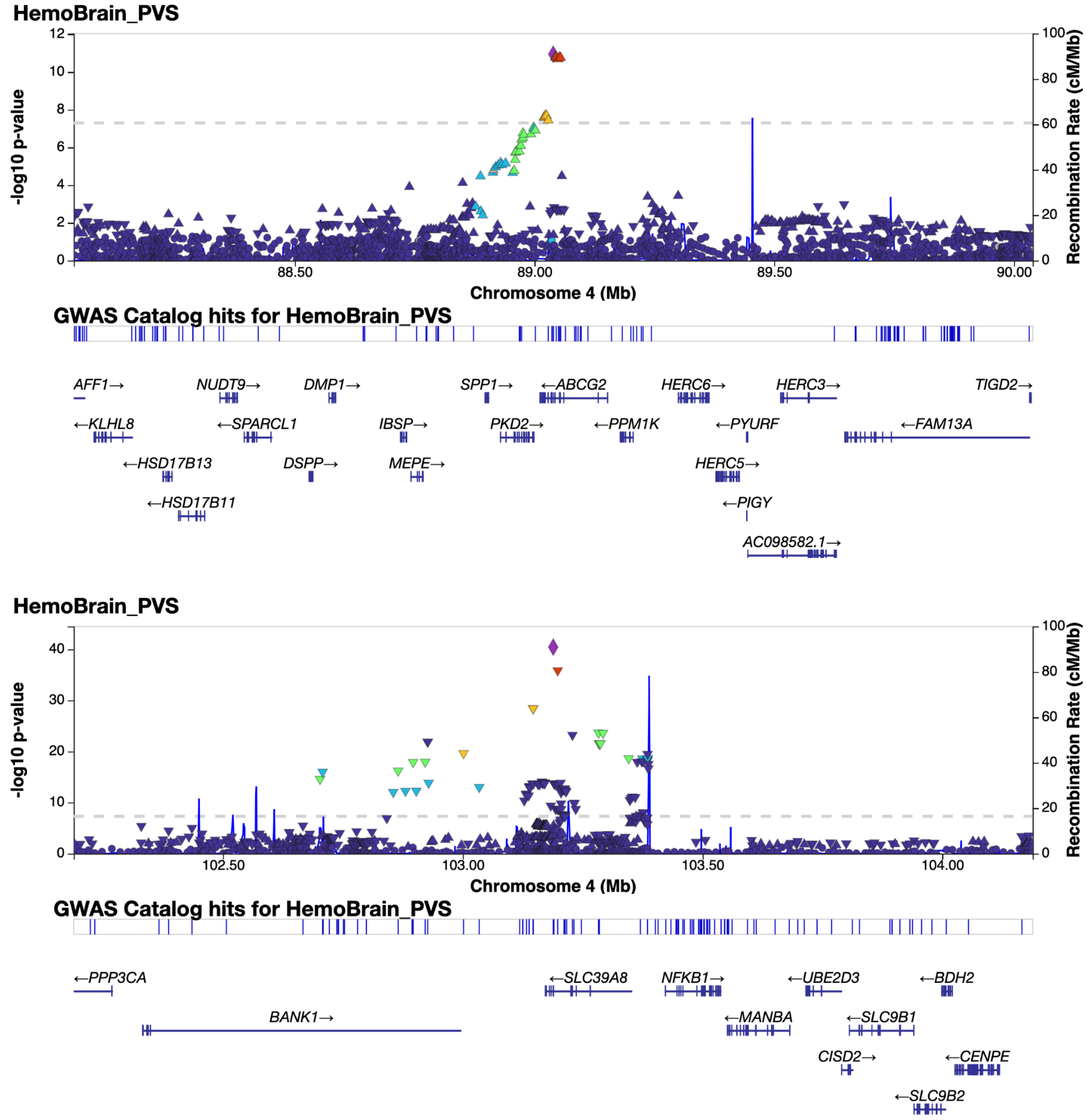

Supplementary Figure 10 ***LocusZoom(98) plot*** *A) for genomic locus 13. B) for genomic locus 14. See extended data tables for genomic locus numbering.*

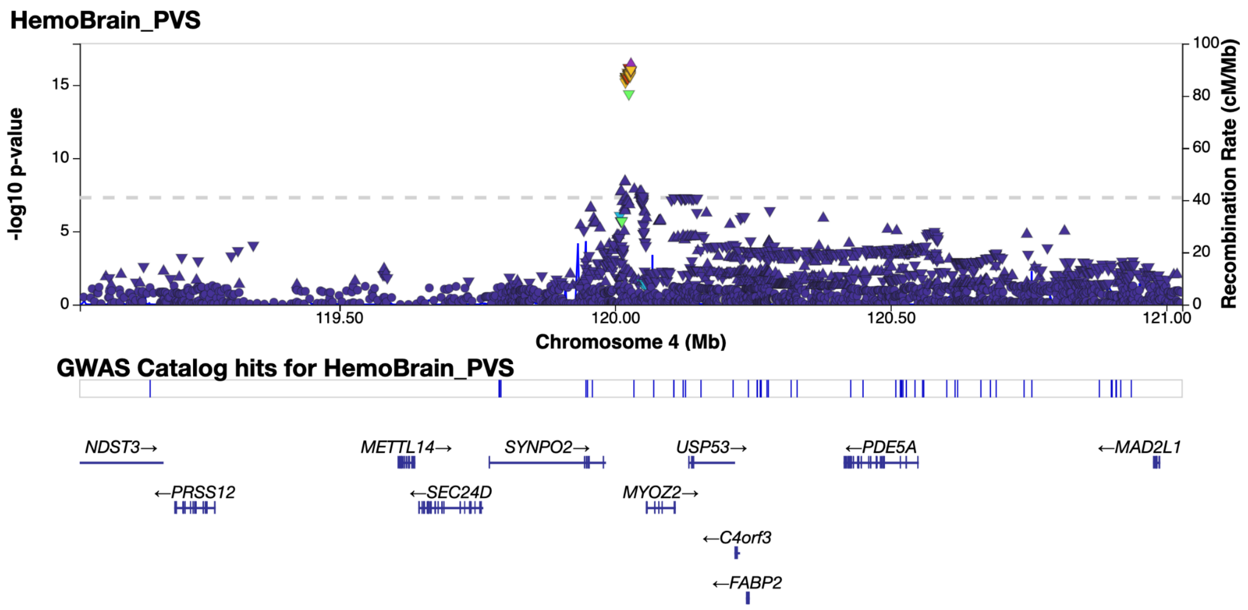

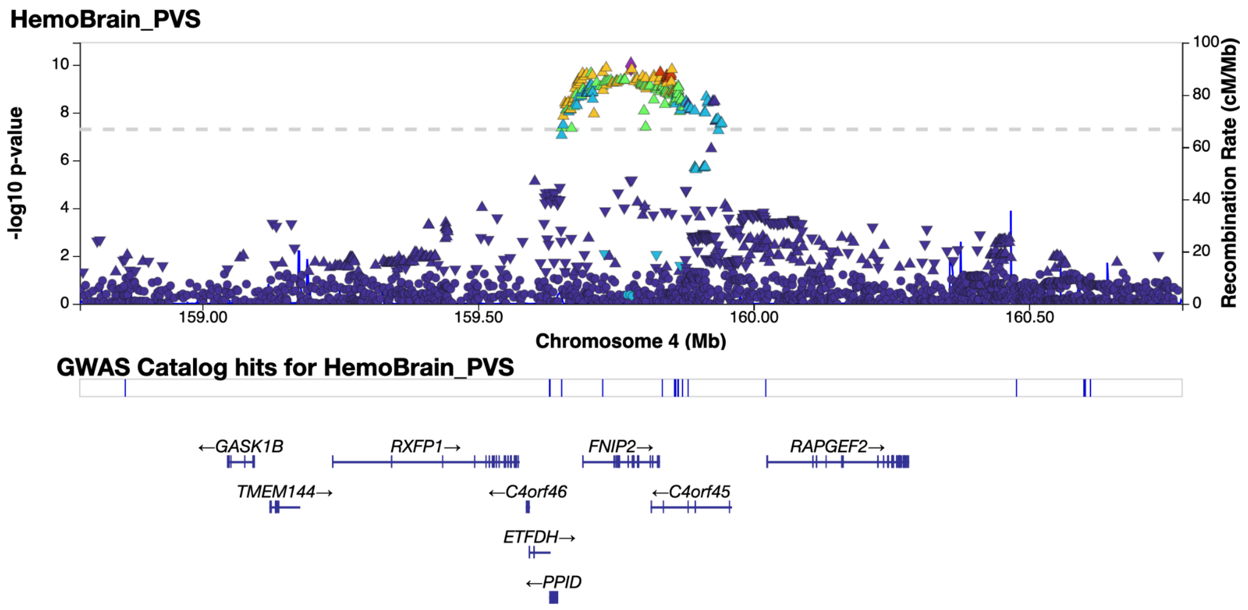

Supplementary Figure 11 ***LocusZoom(98) plot*** *A) for genomic locus 15. B) for genomic locus 16. See extended data tables for genomic locus numbering.*

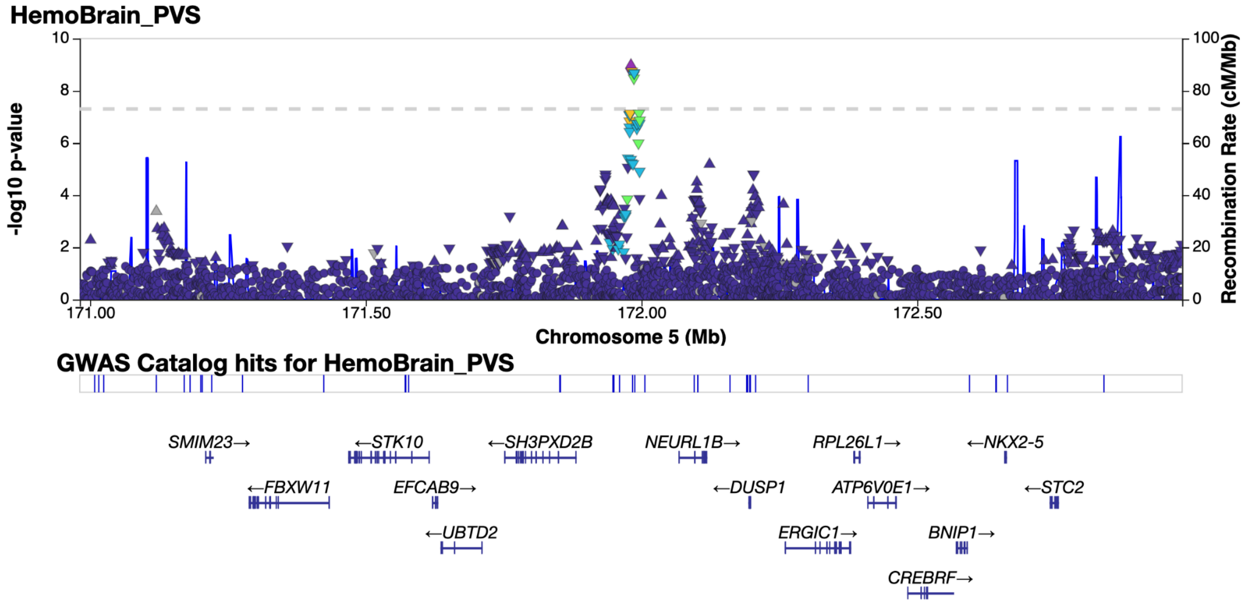

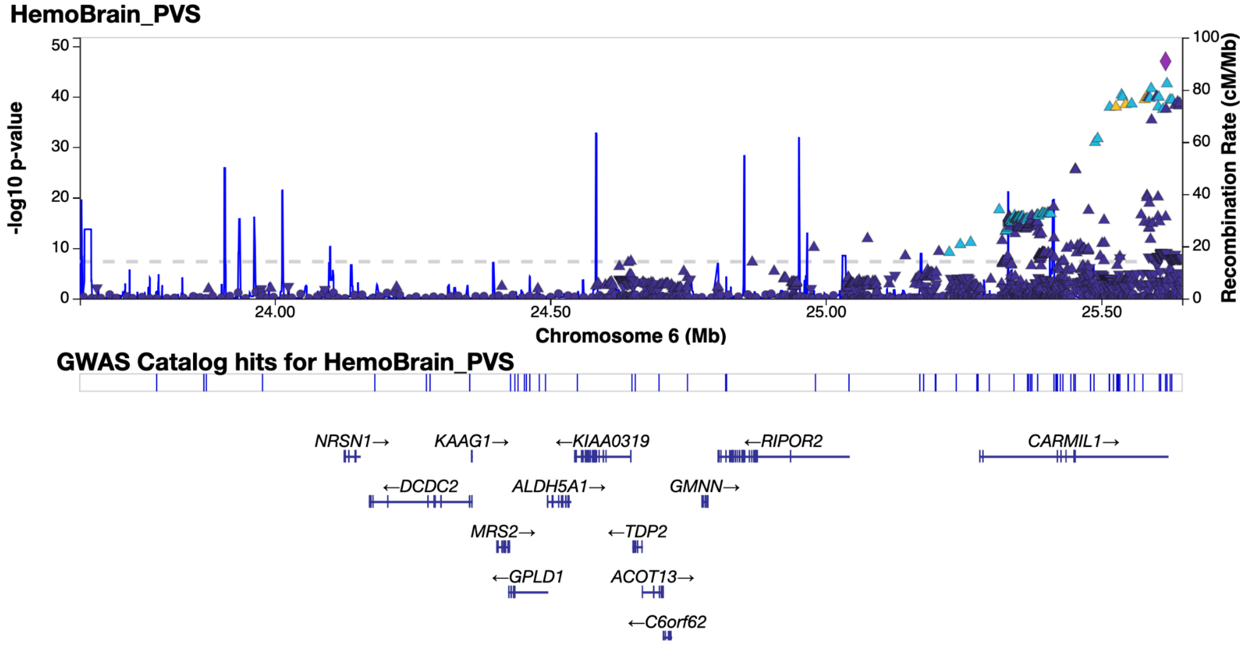

Supplementary Figure 12 ***LocusZoom(98) plot*** *A) for genomic locus 17. B) for genomic locus 18. See extended data tables for genomic locus numbering.*

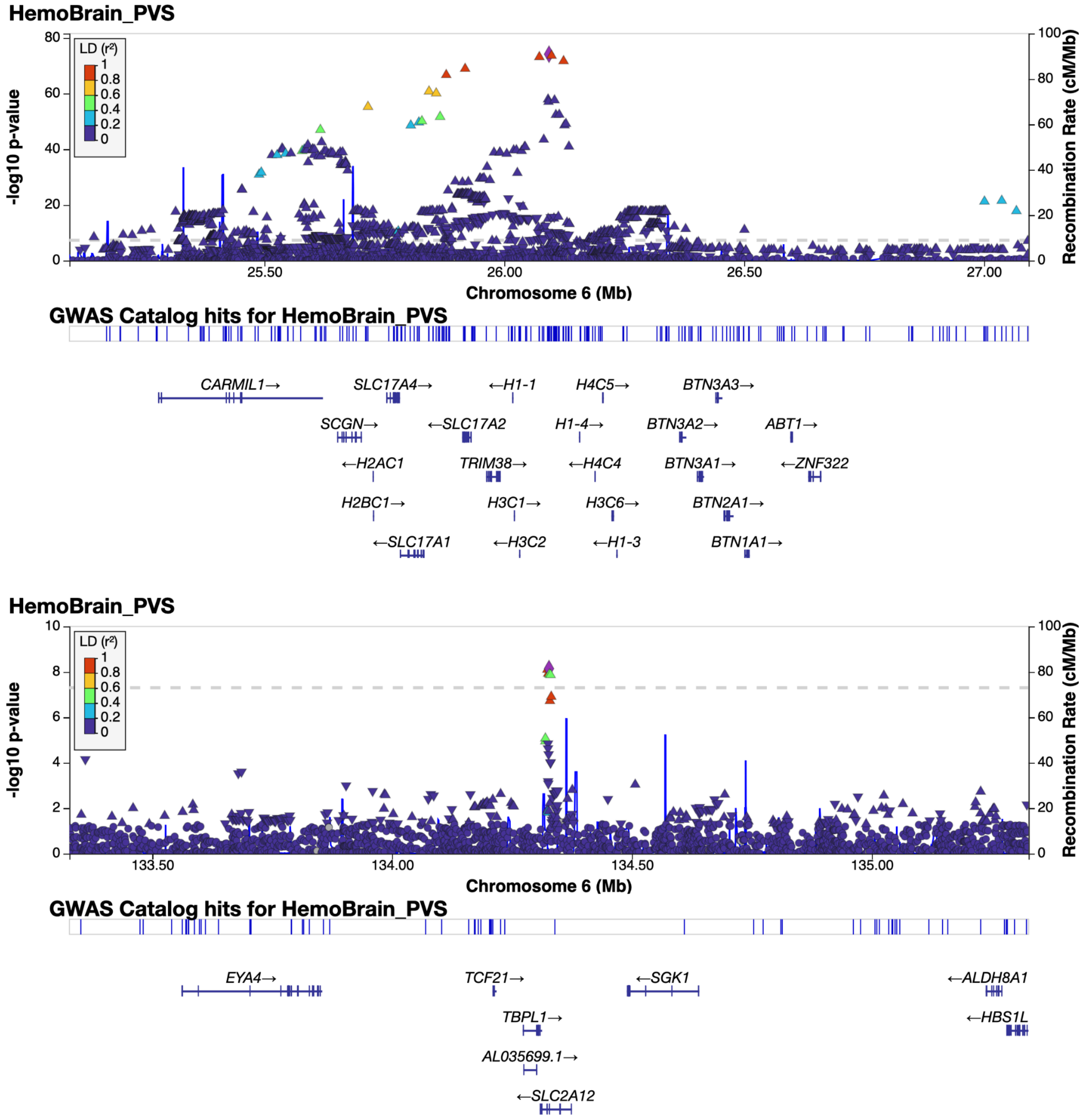

Supplementary Figure 13 ***LocusZoom(98) plot*** *A) for genomic locus 19. B) for genomic locus 20. See extended data tables for genomic locus numbering.*

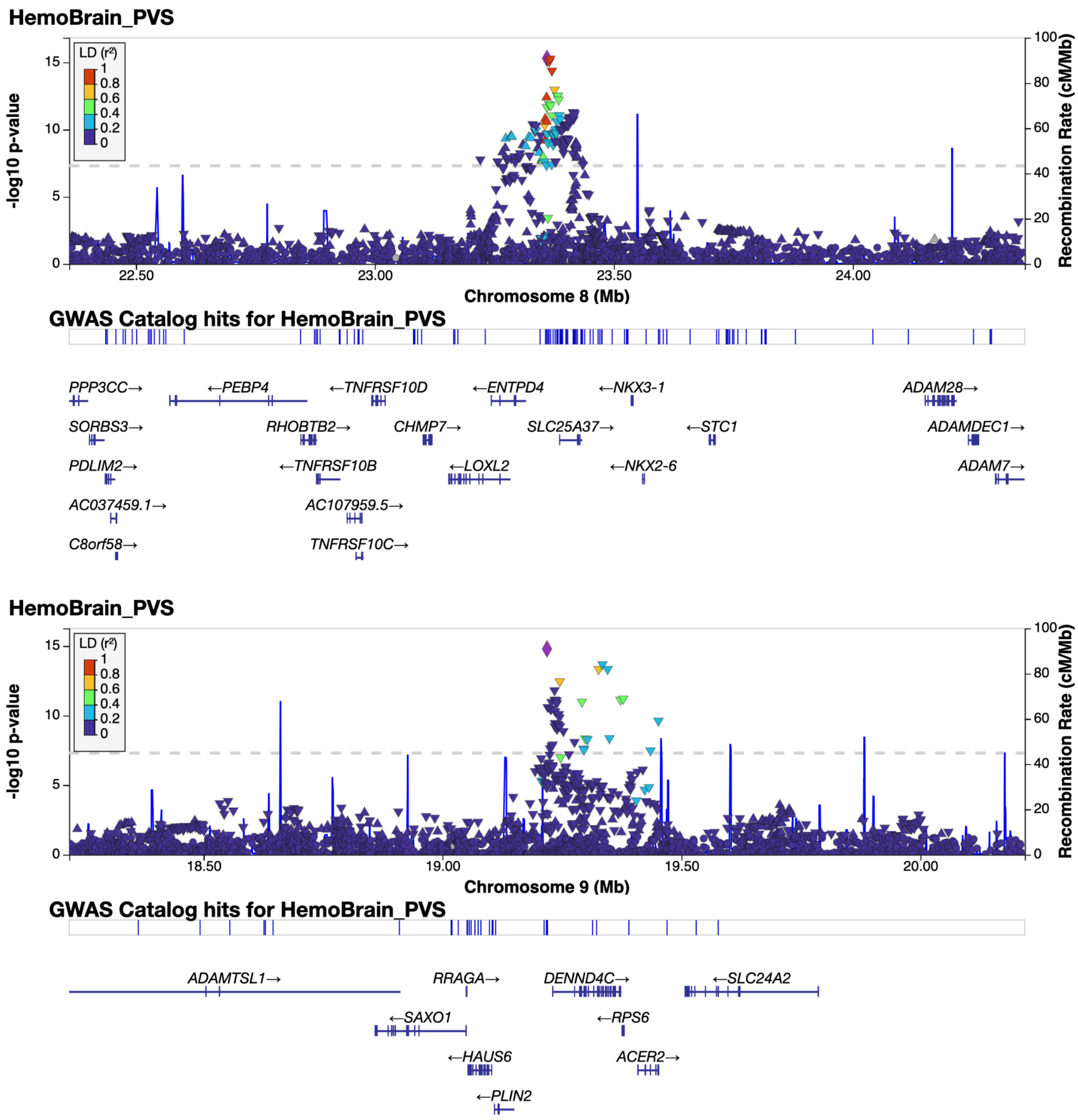

Supplementary Figure 14 ***LocusZoom(98) plot*** *A) for genomic locus 21. B) for genomic locus 22. See extended data tables for genomic locus numbering.*

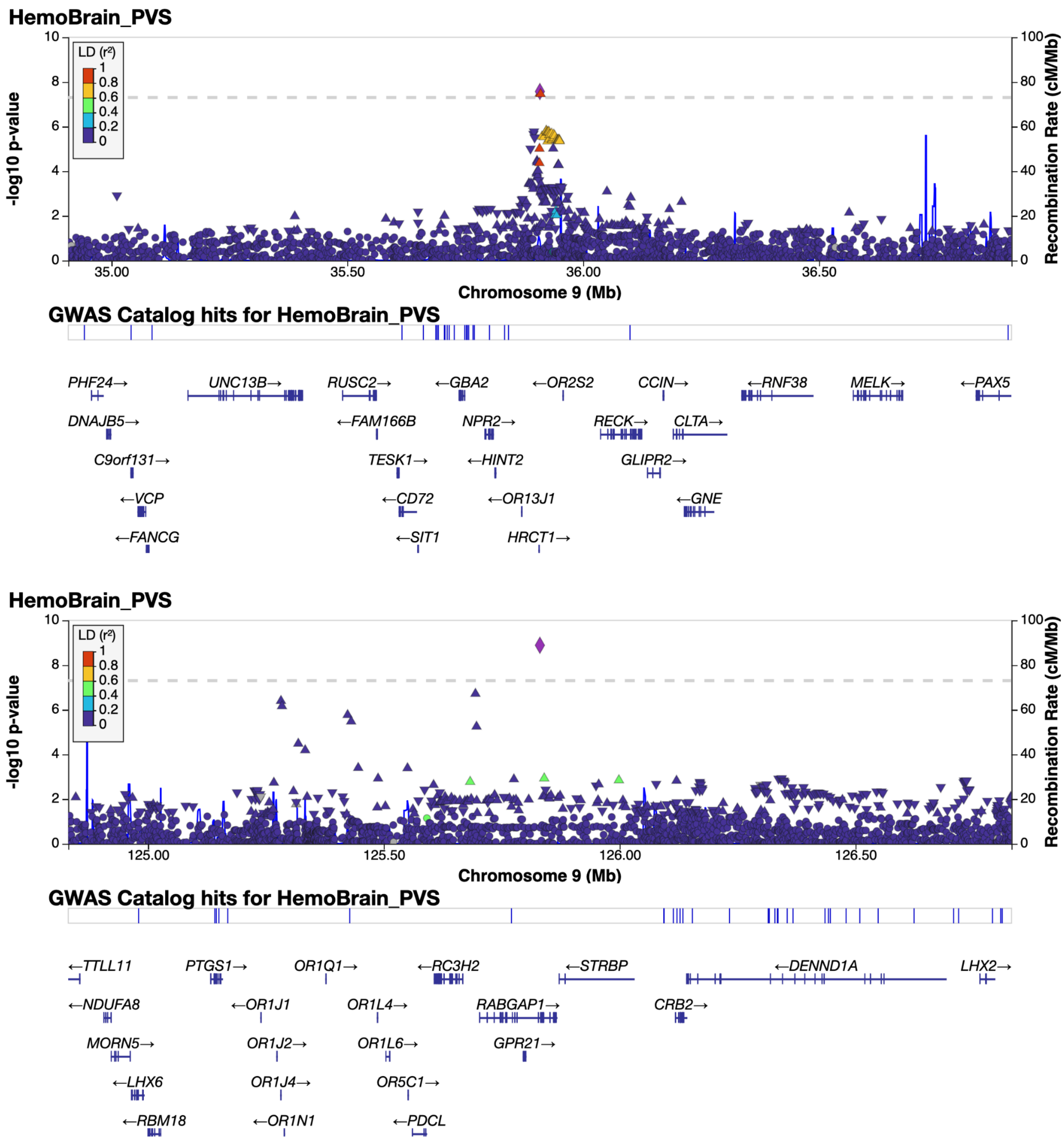

Supplementary Figure 15 ***LocusZoom(98) plot*** *A) for genomic locus 23. B) for genomic locus 24. See extended data tables for genomic locus numbering. Locus 24 displayed a suspicious signal with nearby variants in LD, leading us to believe it was a spurious association and so was removed from the final locus count.*

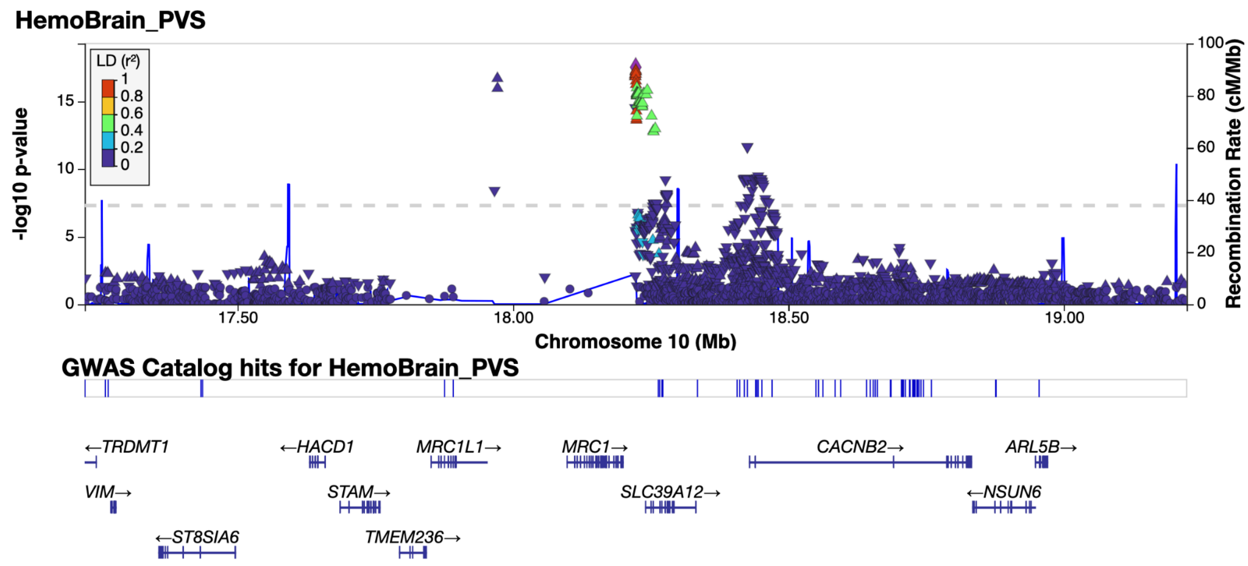

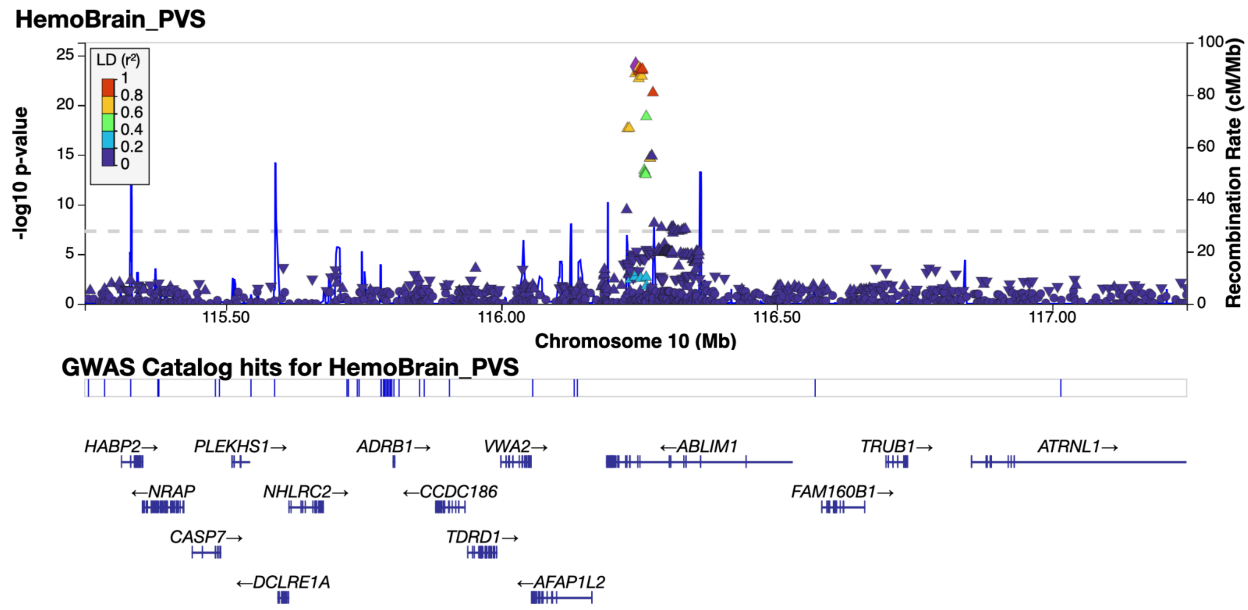

Supplementary Figure 16 ***LocusZoom(98) plot*** *A) for genomic locus 25. B) for genomic locus 26. See extended data tables for genomic locus numbering.*

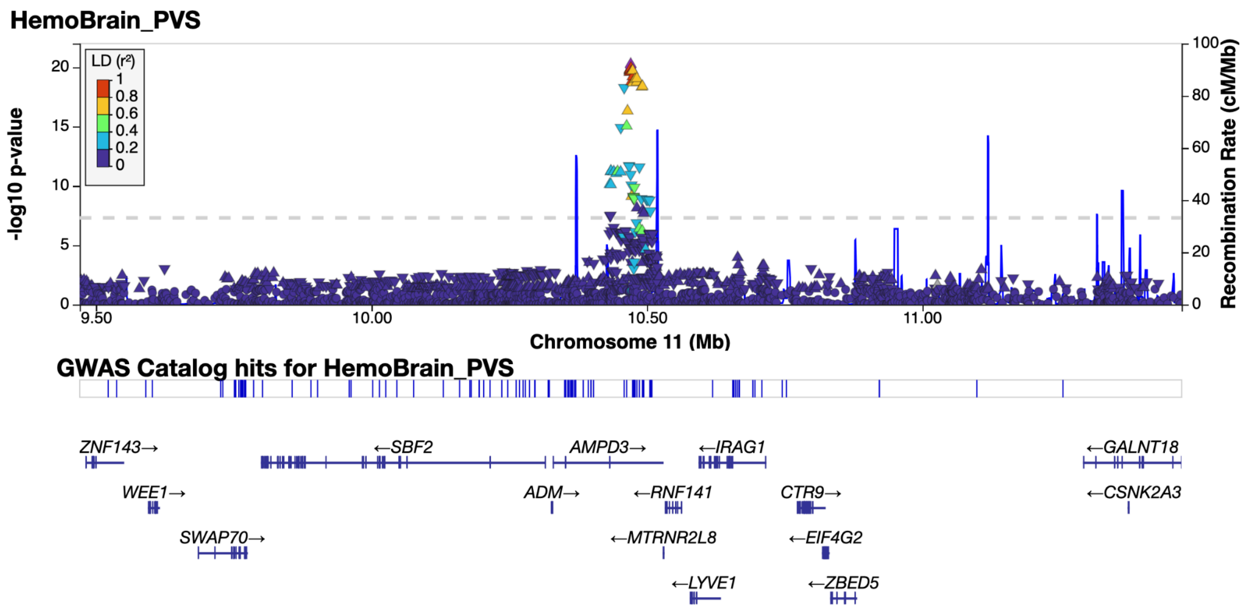

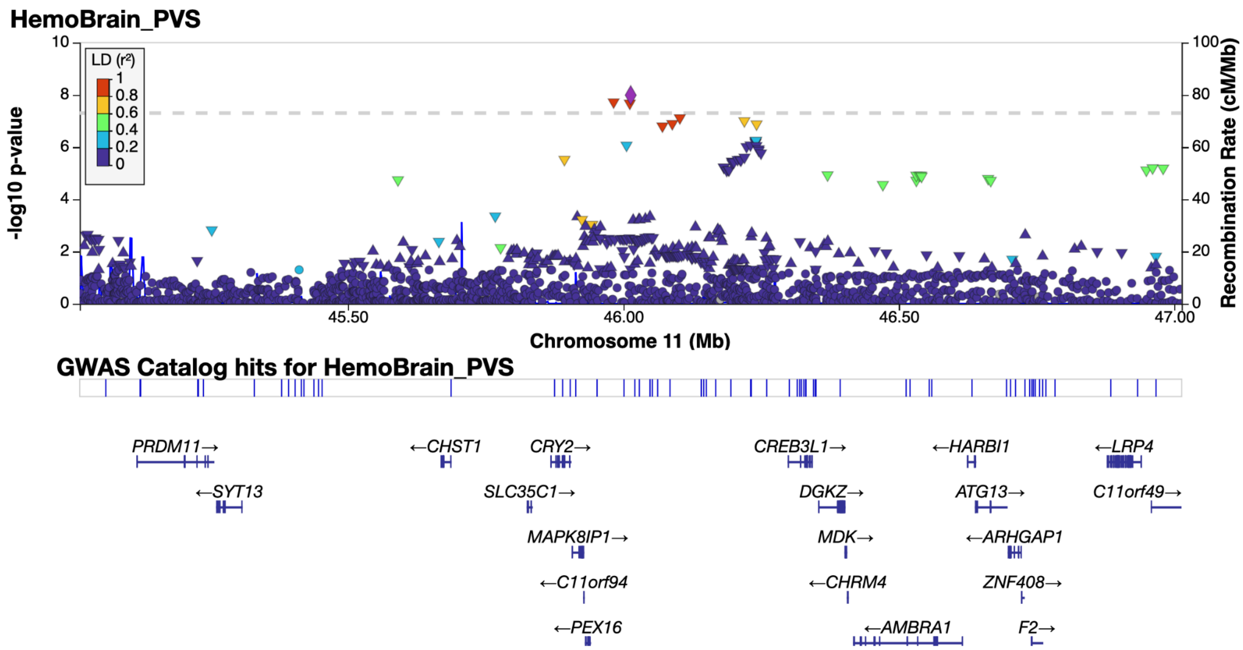

Supplementary Figure 17 ***LocusZoom(98) plot*** *A) for genomic locus 27. B) for genomic locus 28. See extended data tables for genomic locus numbering.*

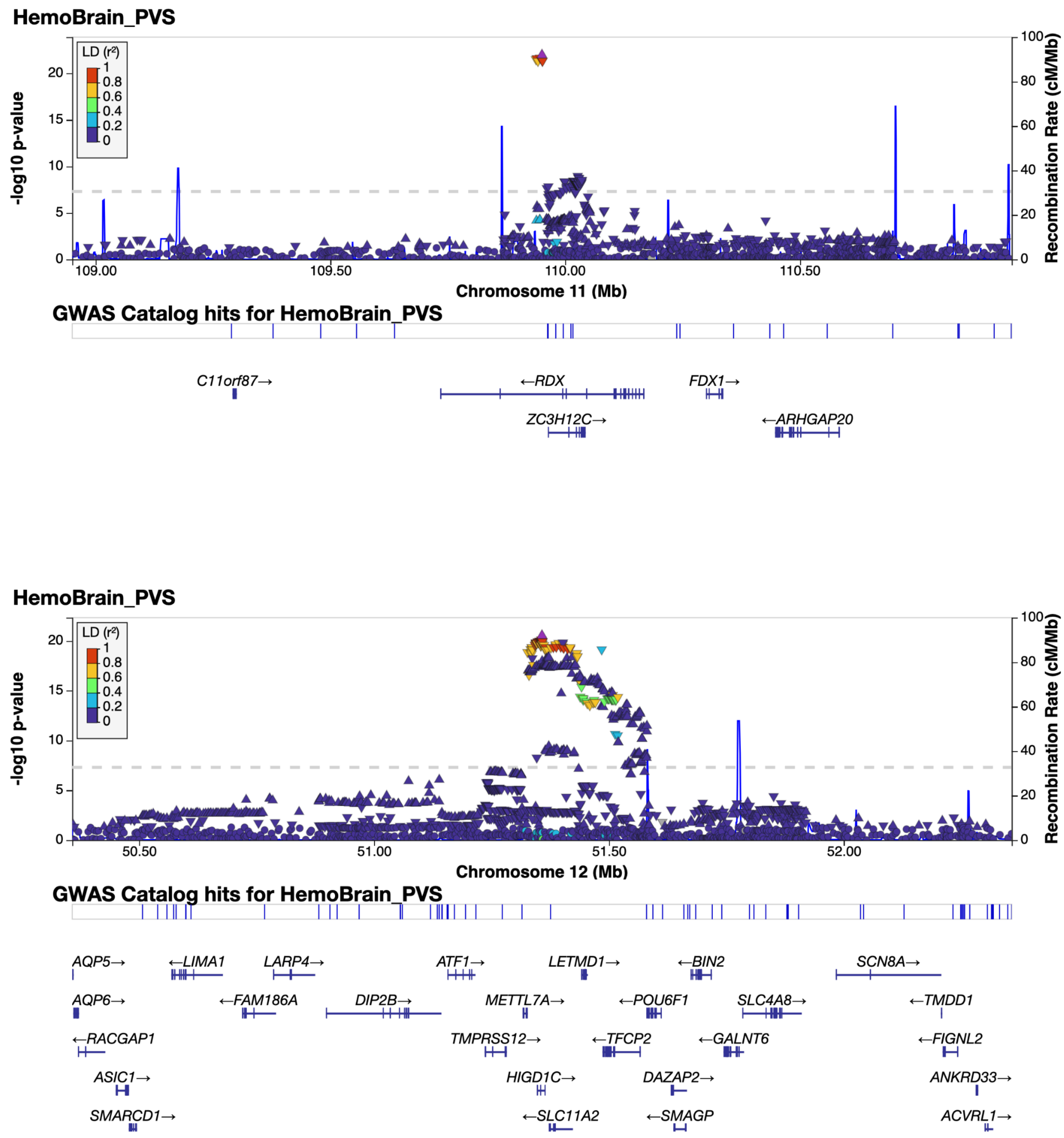

*Supplementary Figure 18 **LocusZoom(98) plot** A) for genomic locus 29. B) for genomic locus 30. See extended data tables for genomic locus numbering.*

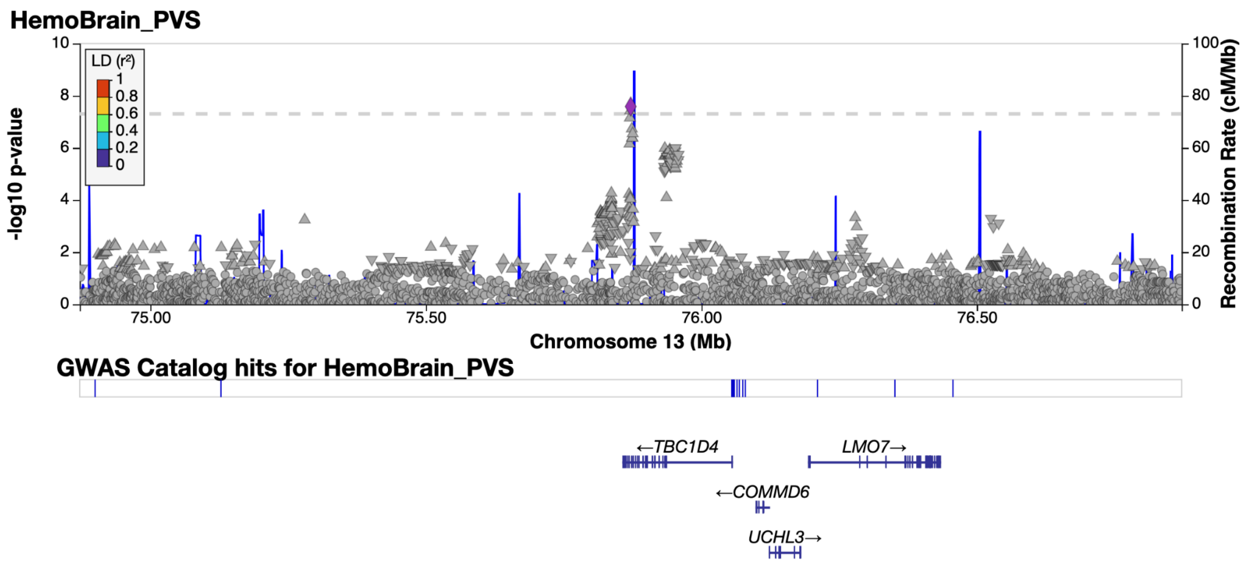

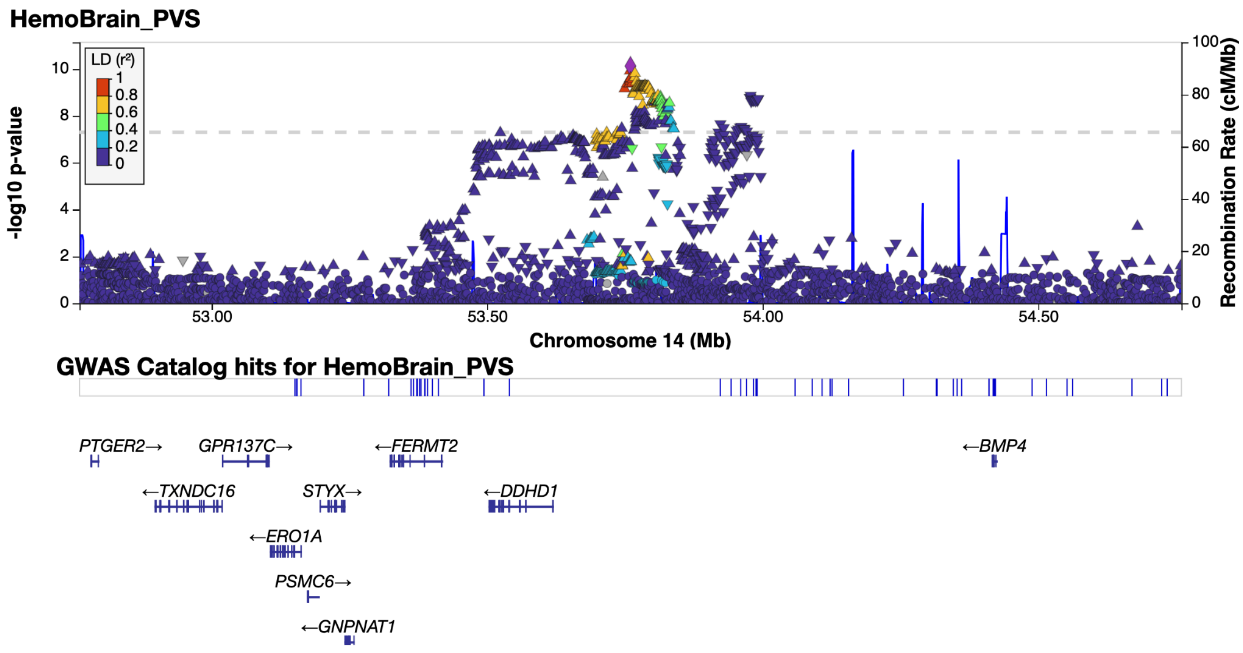

Supplementary Figure 19 ***LocusZoom(98) plot*** *A) for genomic locus 31. B) for genomic locus 32. See extended data tables for genomic locus numbering. Non-lead SNP points are in gray as lead SNP is not in LD reference panel (1k genomes).*

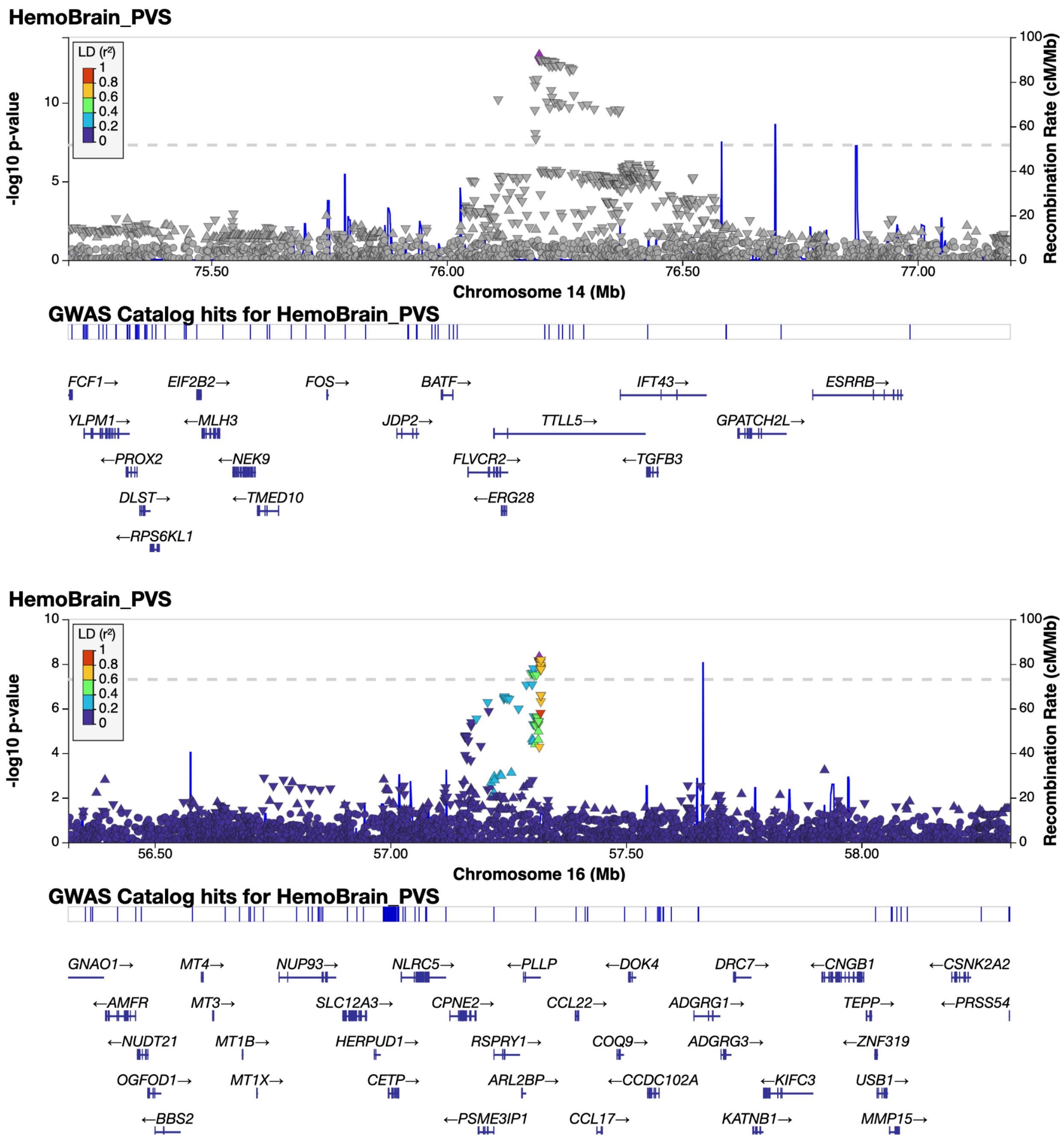

Supplementary Figure 20 ***LocusZoom(98) plot*** *A) for genomic locus 33. B) for genomic locus 34. See extended data tables for genomic locus numbering. Non-lead SNP points are in gray as lead SNP is not in LD reference panel (1k genomes).*

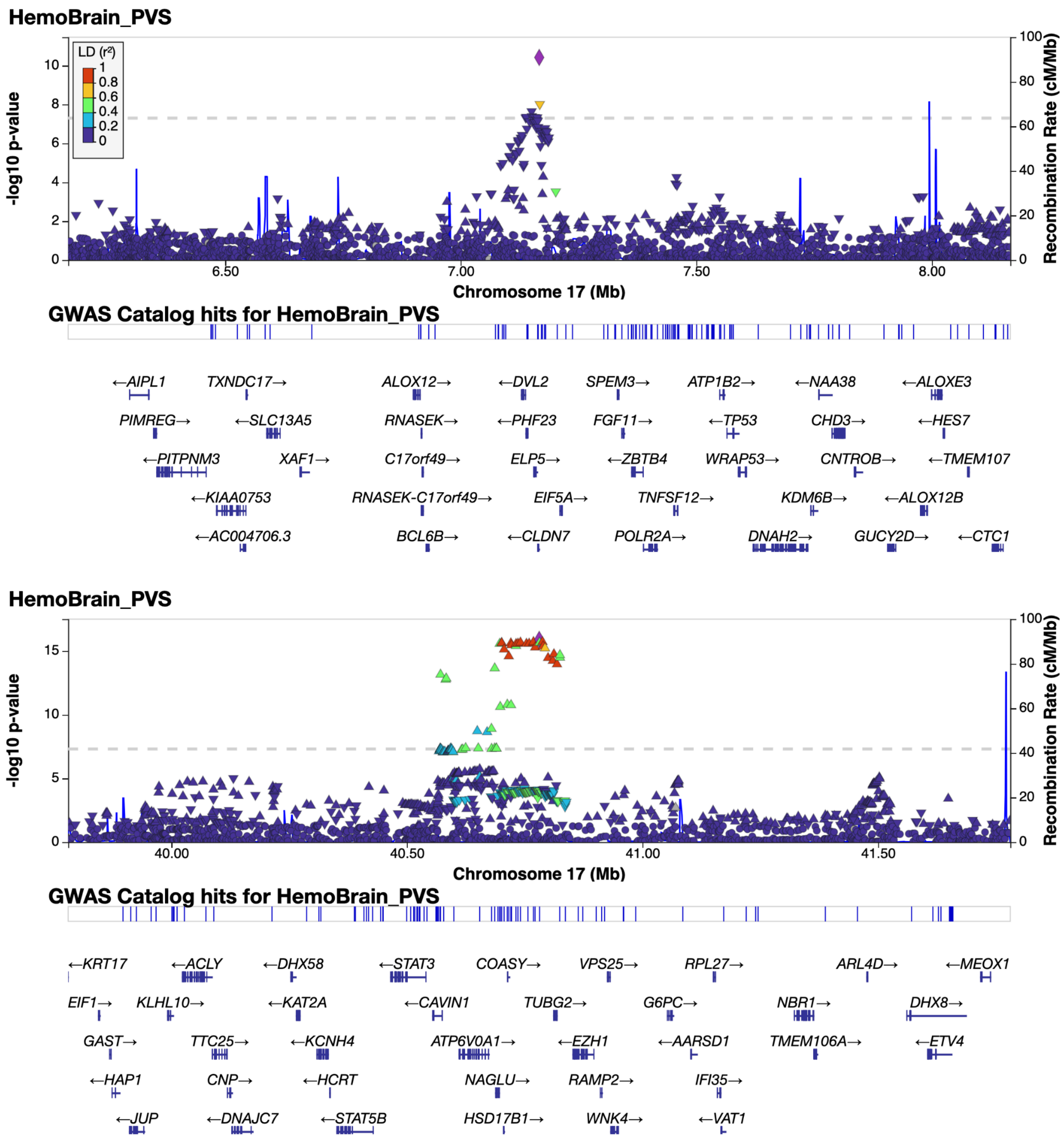

Supplementary Figure 21 ***LocusZoom(98) plot*** *A) for genomic locus 35. B) for genomic locus 36. See extended data tables for genomic locus numbering.*

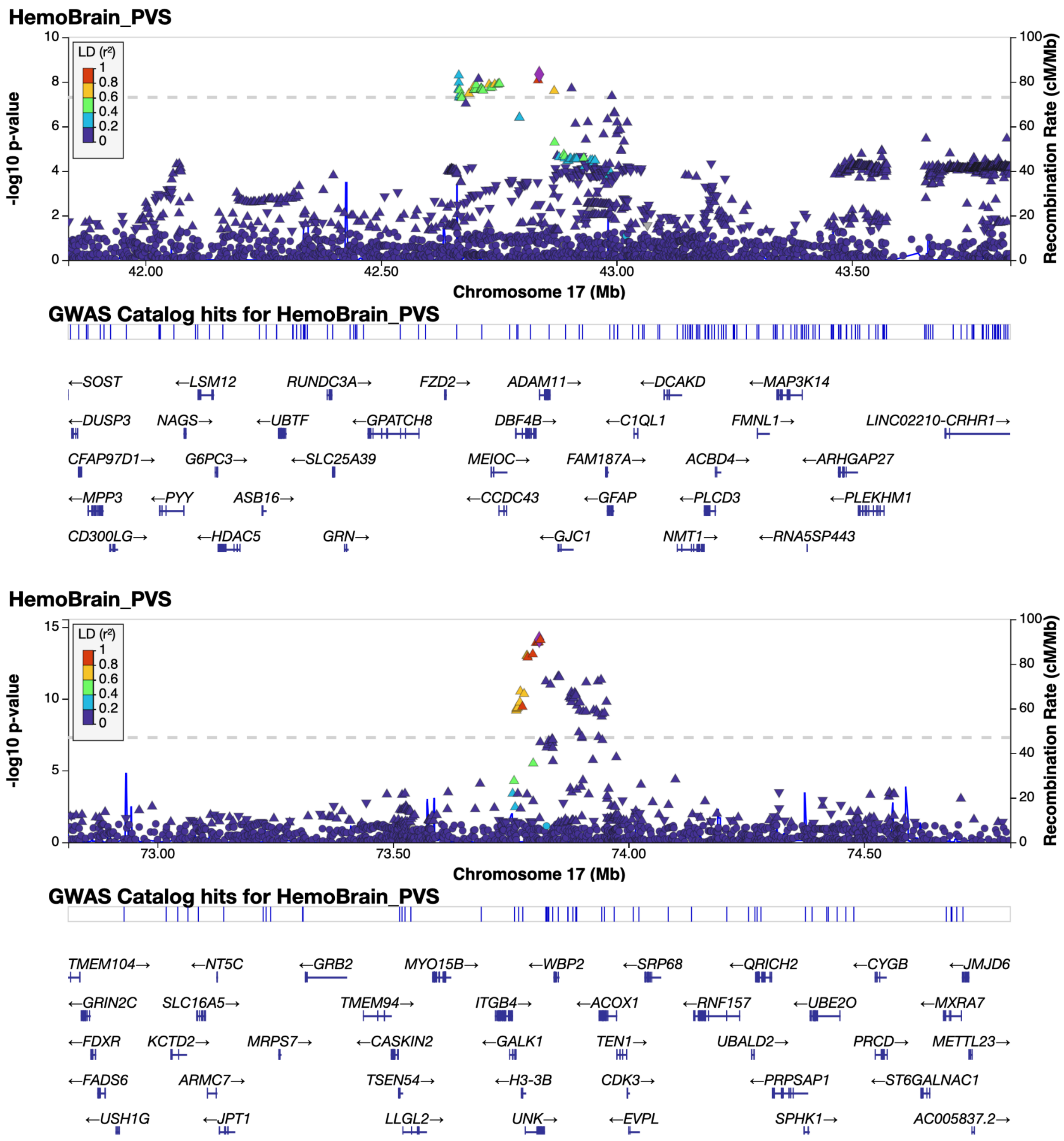

Supplementary Figure 22 ***LocusZoom(98) plot*** *A) for genomic locus 37. B) for genomic locus 38. See extended data tables for genomic locus numbering.*

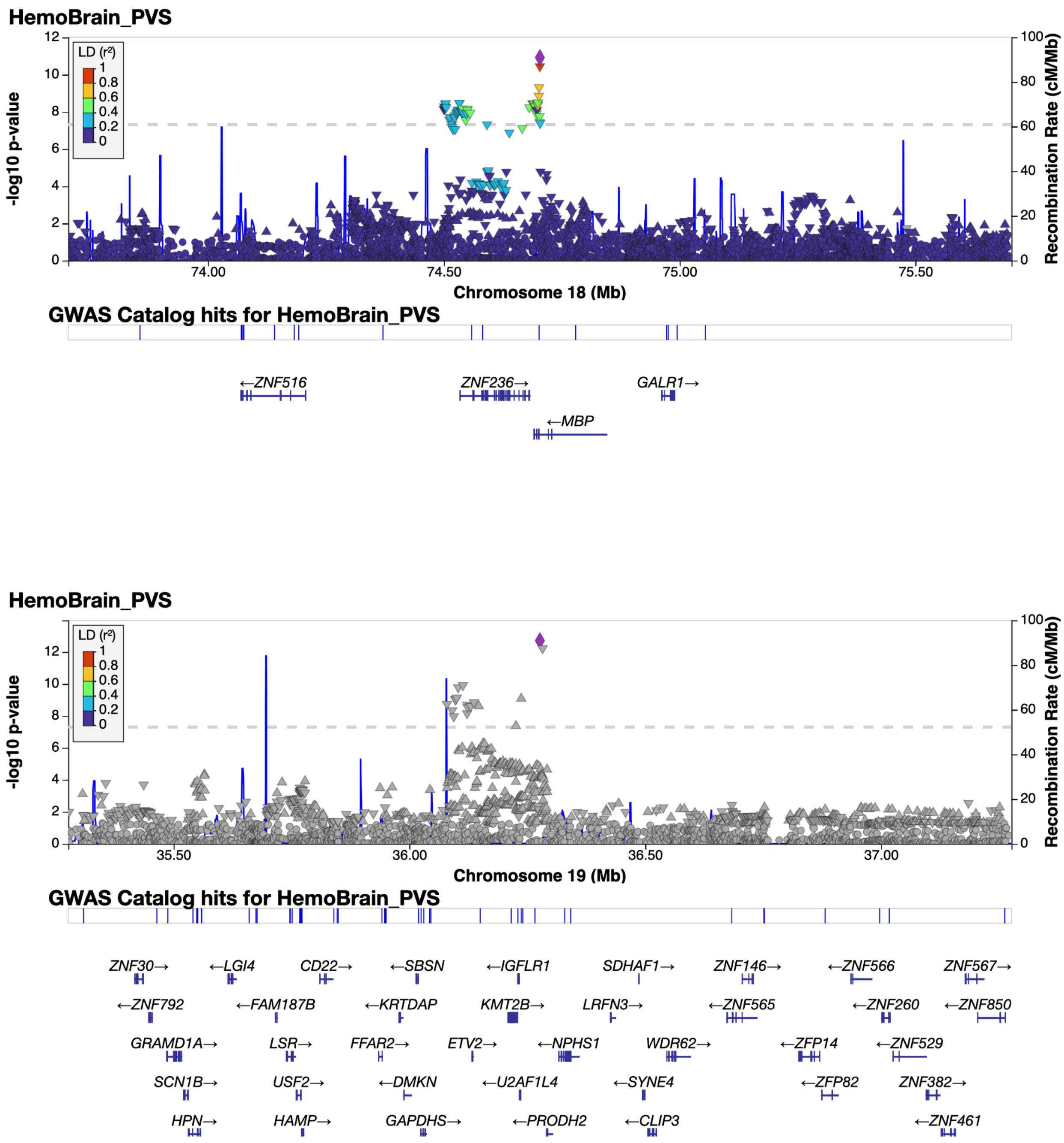

Supplementary Figure 23 ***LocusZoom(98) plot*** *A) for genomic locus 39. B) for genomic locus 40. See extended data tables for genomic locus numbering. Non-lead SNP points are in gray as lead SNP is not in LD reference panel (1k genomes).*

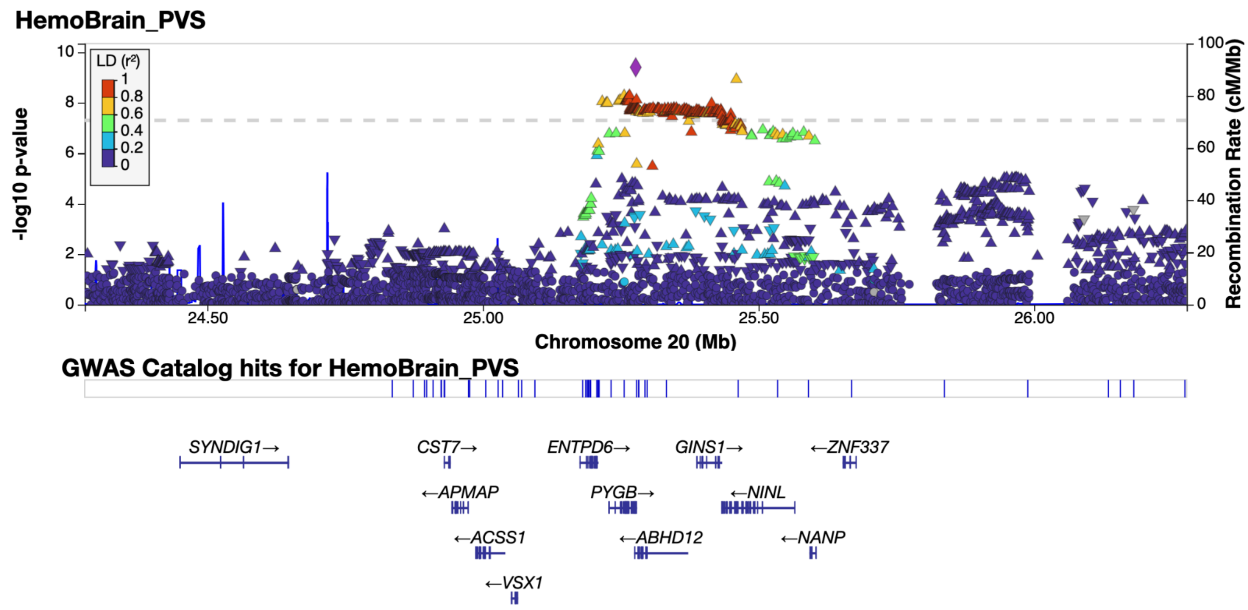

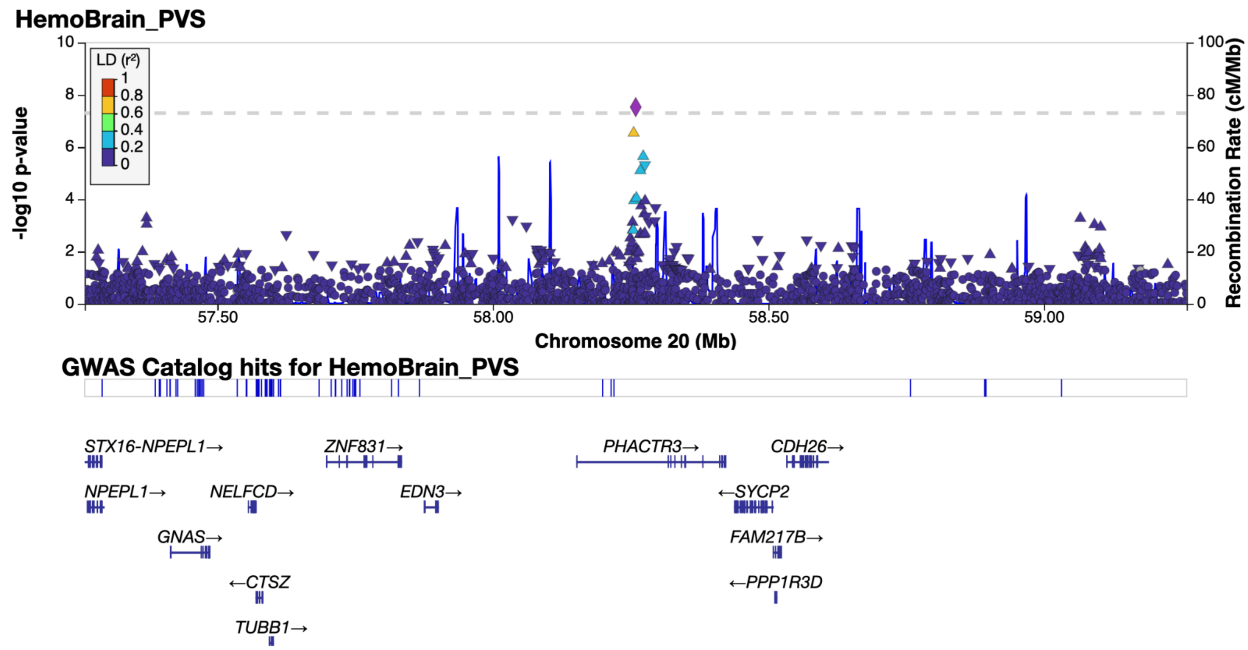

Supplementary Figure 24 ***LocusZoom(98) plot*** *A) for genomic locus 41. B) for genomic locus 42. See extended data tables for genomic locus numbering.*

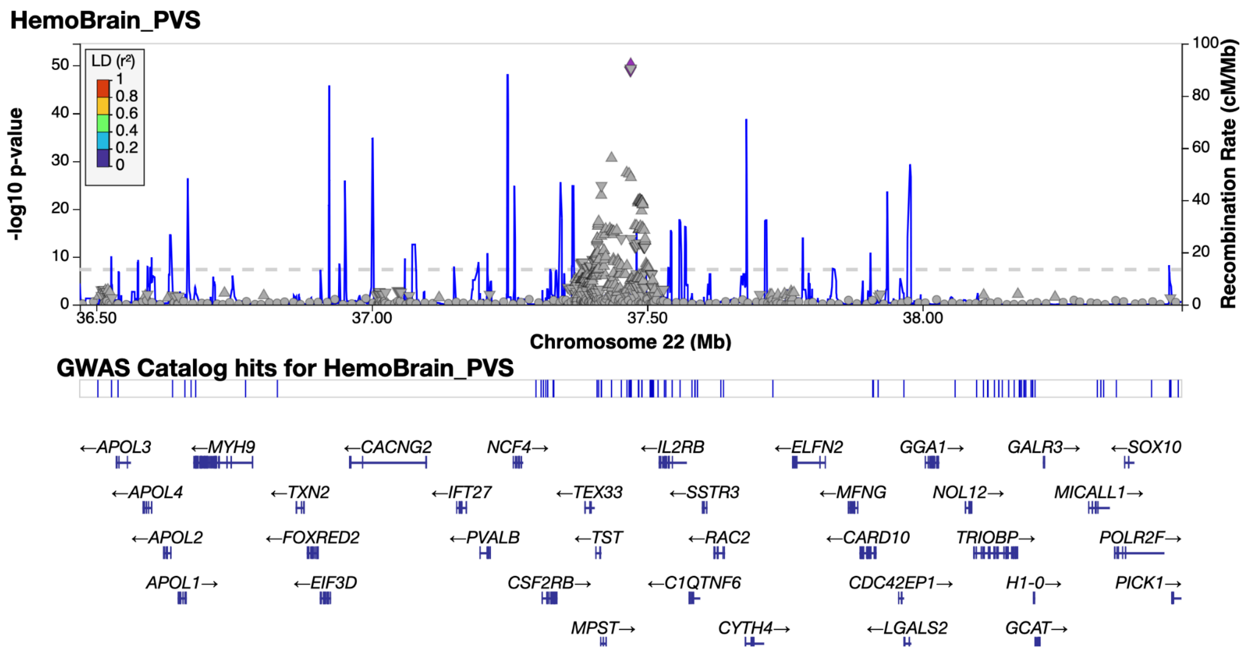

Supplementary Figure 25 ***LocusZoom(98) plot*** *for genomic locus 43. See extended data tables for genomic locus numbering. Non-lead SNP points are in gray as lead SNP is not in LD reference panel (1k genomes).*

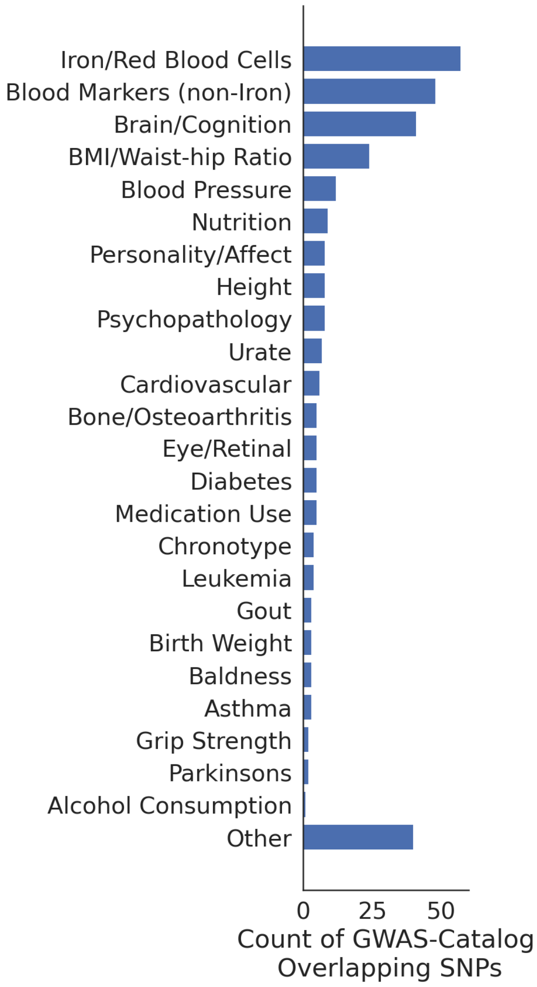

*Supplementary Figure 26: **Overlap of independent significant SNPs** (r_{LD}<0.6) for GWAS of PVS with previously reported SNPs in GWAS-Catalog (e104_r2021-09-15), using categories defined in Supplementary Table 4.*

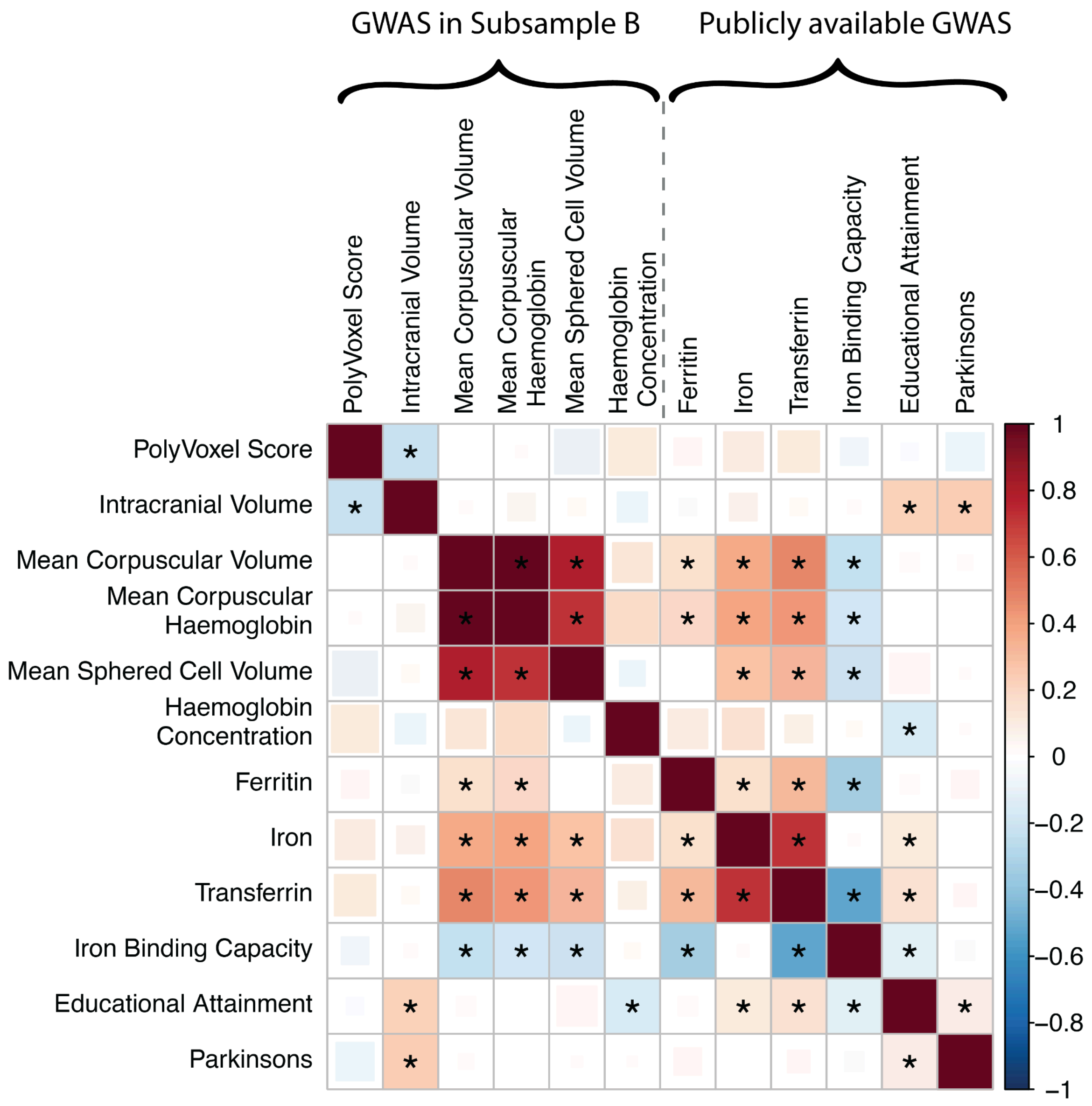

Supplementary Figure 27: ***Genetic correlation using LDSC between PolyVoxel Score (PVS) and traits of interest****. Asterisk indicates FDR significant correlations.*

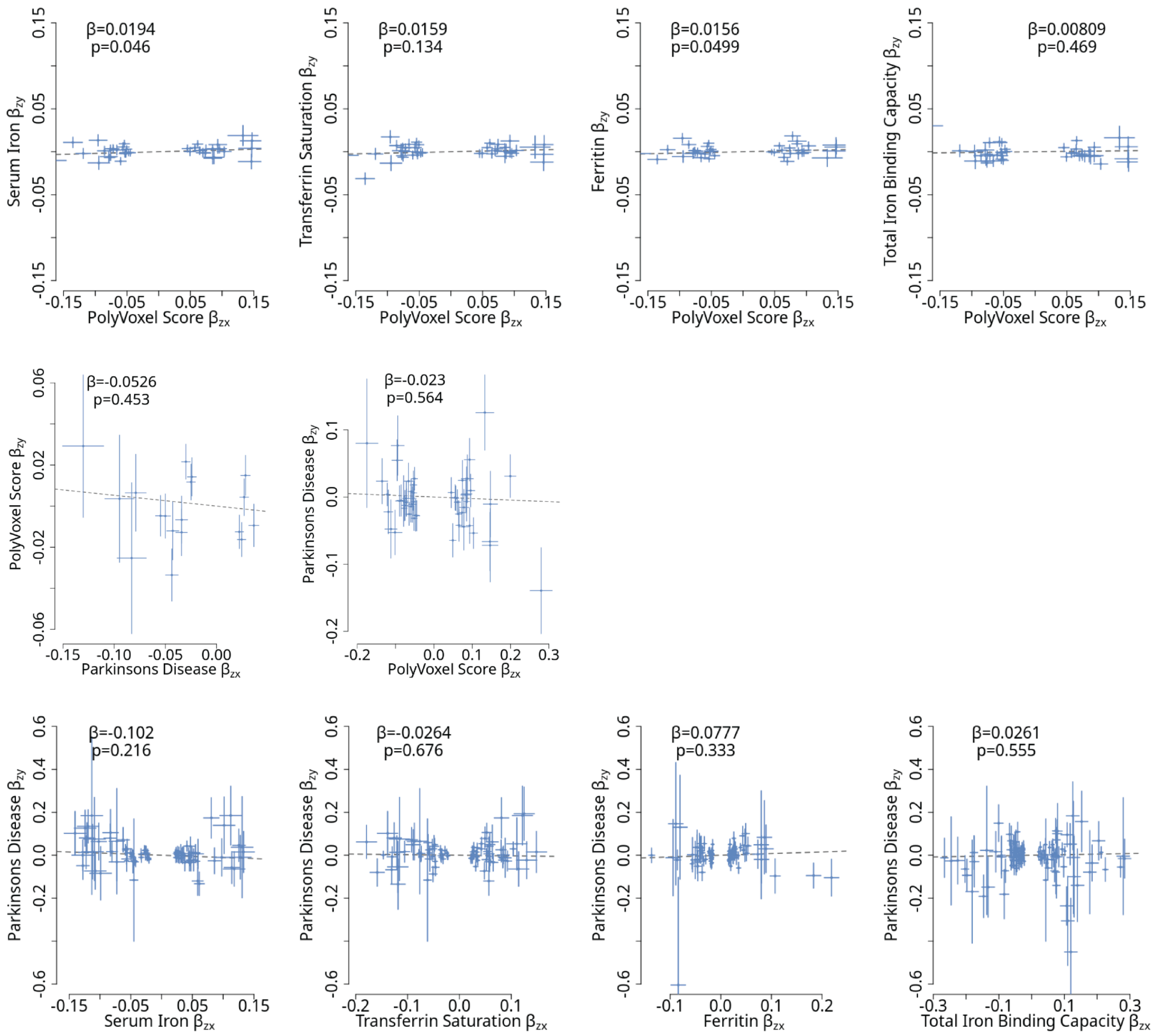

Supplementary Figure 28: ***Supplementary mendelian randomization (GSMR) results****. For each plot x axis indicates phenotype that is used as exposure (i.e. using variants of that phenotype as instrumental variables) and the y axis indicates the outcome. Top row: reverse direction of results in Figure 2D. Middle row: bidirectional GSMR association between PD and PVS. Lower row: GSMR results treating peripheral blood markers as exposures and PD as outcome. Error bars indicate standard errors in estimates.*

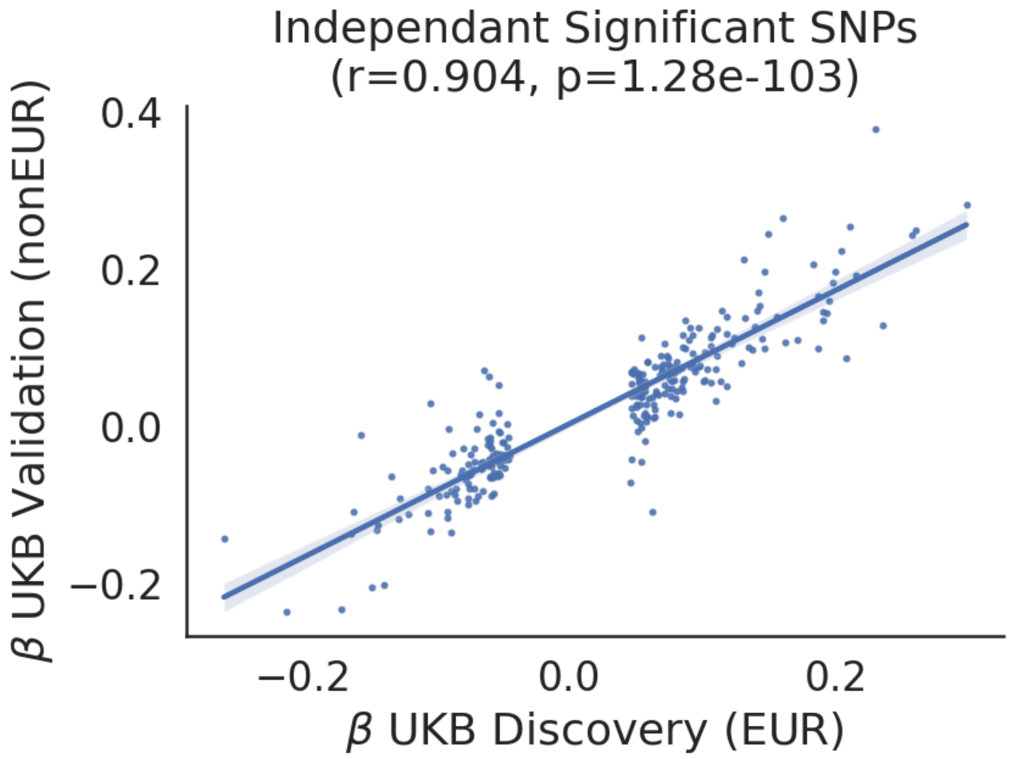

Supplementary Figure 29: ***Replication of PVS GWAS Discoveries.*** *Discoveries within European ancestry individuals of subsample B of UKB (30,709 European ancestry individuals) and validation in remaining non-European ancestry individuals of subsample B (4,608 individuals)*

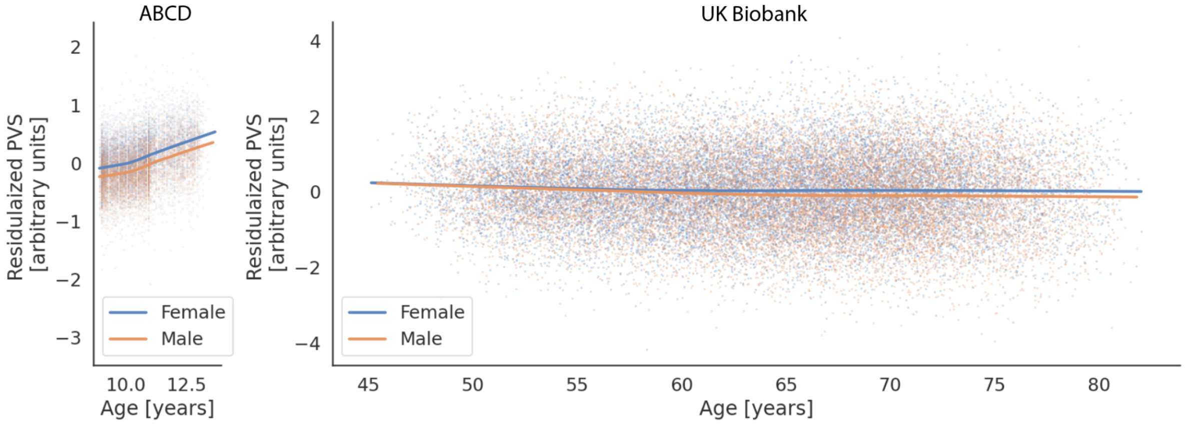

*Supplementary Figure 30: **Effect of age on PVS for ABCD (left) and UK Biobank (right)**. PVS are residualized (see methods for covariates), within each sample so y axes are on different scales - however x axes within each plot have been scaled to match. Higher values on the y axis are in the direction of higher brain iron values.*

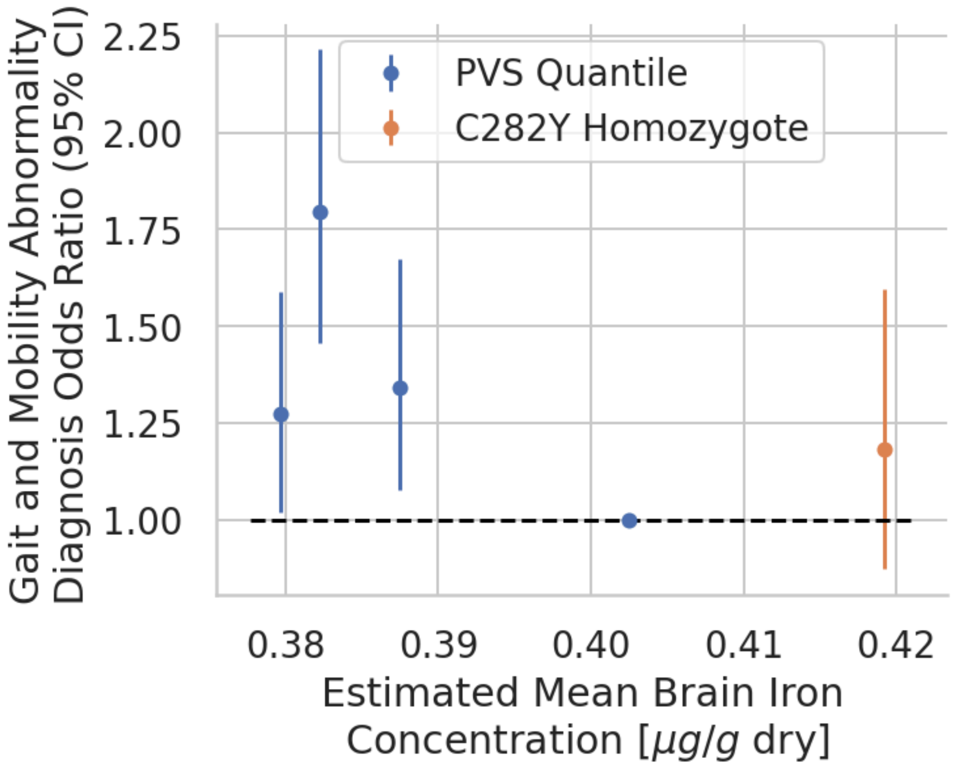

*Supplementary Figure 31: **PVS quantile weighted regression to predict Abnormalities of Gait and Mobility [ICD10:R26] in Subsample C**, each point represents a categorical factor indicating one of four PVS quantile (blue) or C282Y homozygosity (orange). IPW of 3.63 was used for PD cases in imaging sample (blue) - see methods. Regression (y-axis) was performed using PVS from T2-W, x-axis is an estimate of mean brain iron concentration using T2* imaging for each group.*

Supplementary Tables

	UK Biobank Subsample			UK Biobank Full Sample
	A	B	C	
n	960	35,283	38,170	502,413
Age, mean (SD)	64.3 (7.5)	64.1 (7.5)	64.3 (7.6)	66.2 (8.13)
EUR, n (%)	864 (90)	30761 (87.2)	33372 (87.4)	409551 (81.5)
Female, n (%)	575 (60.0)	18,378 (52.1)	19,972 (52.3)	273,328 (54.4)
C282Y Homozygote (%)	193 (20.0)	0 (0.0)	2,888 (7.56*)	2,888 (0.57)
C282Y Heterozygote (%)	0 (0.0)	4,935 (14.0)	4,935 (14.0)	65,299 (13.0)
Parkinson's Disease Cases (%)	0 (0.0)	64 (0.18)	93 (0.24)	3,447 (0.68)
Analysis Used in	C282Y Classifier Training	a) GWAS of Hemochromatosis Brain b) Neurological Disease Enrichment	Quantile Weighted Regression	Estimation of IPWs for i) Neurological Disease Enrichment and ii) Quantile Weighted Regression

Supplementary Table 1 ***Demographic breakdown of each subsample of UK Biobank****. Subsample A was used for PVS training (and cross validation). Subsample B was used for generation of PVS scores and GWAS discovery. Subsample C was used for weighted quantile regression (main figure 3b) - it only differs from subsample B with the inclusion of 2,888 C282Y homozygotes from the entire sample. * Note this percentage represents the homozygote rate for subsample C, the homozygote rate across the whole UKB sample is 0.57%. EUR indicates those individuals that self-declared as "white-british" and who share similar genetic ancestry determined by principal components - data field 22006. IPW: Inverse Probability Weighting - see supplementary table 6.*

	ABCD Subsample		
	**EUR**	**AFR**	**MIX**
n	5,977	687	3,135
Baseline Age, mean (SD)	9.93 (0.63)	9.91 (0.60)	9.91 (0.60)
Year 2 Age, mean (SD)	11.94 (0.64)	11.94 (0.63)	11.90 (0.65)
Female, n (%)	2,856 47.78)	351 (51.09)	1,535 (48.96)
C282Y Homozygote (%)	16 (0.27)	0 (0.0)	0 (0.00)
C282Y Heterozygote (%)	684 (11.44)	6 (0.87)	133 (4.24)

Supplementary Table 2: ***Demographic breakdown of each ancestry strata for ABCD sample***.

FreeSurfer 5.3 segmentation T1	***Pauli, 2018 HCP T1 & T2***
Cerebellum White Matter<br>Cerebellum Gray Matter<br>Pallidum<br>Thalamus	*Putamen<br>Caudate<br>Substantia nigra pars compacta<br>Substantia nigra pars reticulata<br>Red nucleus<br>Subthalamic nucleus*

Supplementary Table 3 ***Regions of interest (ROIs) labeled using 2 different methods.*** *Column 1) automatic segmentation using FreeSurfer 5.3 applied to each subject's T1 image in atlas space(99); Column 2) registration of the Pauli atlas of subcortical nuclei to the multispectral atlas(100)*

CATEGORY	SEARCH TERMS	
**IRON/RED BLOOD CELLS**	*iron*<br>*hematocrit*<br>*ferritin*<br>*red cell*<br>*red blood*<br>*bilirubin*	*hemoglobin*<br>*hemotcrit*<br>*hepcidin*<br>*corpuscular*<br>*reticulo*<br>*hereditary hemochromatosis*
**BRAIN/COGNITION**	*brain*<br>*white matter*<br>*intelligence*<br>*lentiform nucleus*<br>*nucleus accumbens*<br>*highest math class taken*	*cognitive*<br>*cortical*<br>*educational attainment*<br>*math ability*<br>*grey matter*
**BLOOD MARKERS (NON-IRON)**	*lipoprotein*<br>*metabolite*<br>*cholesterol*<br>*triglyceride*<br>*alkaline phosphatase levels*<br>*eosinophil*<br>*serum 25-hydroxyvitamin d levels*<br>*serum albumin level*<br>*lipid traits*<br>*monocyte*<br>*liver enzyme levels*	*protein*<br>*platelet*<br>*white blood cell*<br>*aminotransferase levels*<br>*globulin levels*<br>*testosterone levels*<br>*basophil*<br>*calcium levels*<br>*insulin-like growth*<br>*adiponectin levels*<br>*thyroxine concentration*
**URATE**	*urate*<br>*uric acid*	
**BMI/WAIST-HIP RATIO**	*body mass index*<br>*waist-hip*<br>*body shape index*<br>*hip circumference*	*bmi*<br>*body fat*<br>*waist circumference*<br>*hip index*
**BLOOD PRESSURE**	*blood pressure*<br>*hypertension*<br>*arterial pressure*<br>*pulse pressure*	
**PSYCHOPATHOLOGY**	*neuroticism*<br>*schizophrenia*<br>*depressive symptoms*<br>*bipolar disorder*<br>*anorexia nervosa*	
**HEIGHT**	*height*	

LEUKEMIA	*leukemia*
ALCOHOL CONSUMPTION	*alcohol consumption*<br>*alcohol use*
CHRONOTYPE	*morning person*<br>*daytime sleep*<br>*morningness*<br>*sleep*<br>*getting up in the morning*
DIABETES	*diabetes*
CARDIOVASCULAR	*cardiovascular*
BALDNESS	*baldness*<br>*balding*
GRIP STRENGTH	*grip strength*
BONE/OSTEOARTHRITIS	*bone*<br>*osteoarthritis*
PERSONALITY/AFFECT	*adventurousness*<br>*positive affect*<br>*worry*<br>*empathy quotient*<br>*risk tolerance*<br>*life satisfaction*<br>*leisure sedentary behaviour*
EYE/RETINAL	*optic cup area*<br>*iris*<br>*vertical cup-disc ratio*<br>*eye*<br>*refractive error*
MEDICATION USE	*medication use*
PARKINSONS	*parkinson*
GOUT	*gout*
BIRTH WEIGHT	*birth weight*
NUTRITION	*consumption*<br>*meat-related diet*
ASTHMA	*asthma*

Supplementary Table 4: Definition of search terms to define each category when comparing GWAS discoveries with overlapping traits from GWAS catalog - used to generate Supplementary Figure 5.

Exposure	Outcome	Number of SNPs	Beta	Beta_se	pval
Ferritin	PVS	50	0.34227912	0.04133268	1.22E-16
PVS	Ferritin	79	0.0155955	0.00795368	4.99E-02
Serum Iron	PVS	46	0.73274578	0.03727421	4.91E-86
PVS	Serum Iron	81	0.01935118	0.0096979	4.60E-02
TIBC	PVS	47	-0.3617474	0.0249315	1.05E-47
PVS	TIBC	103	0.00809068	0.01116882	4.69E-01
TSAT	PVS	44	0.62012578	0.03027319	2.97E-93
PVS	TSAT	91	0.01594714	0.01064346	1.34E-01
PD	Ferritin	28	0.0777404	0.08023248	3.33E-01
Ferritin	PD	111	-0.0068181	0.02421652	7.78E-01
PD	Serum Iron	28	-0.1020984	0.08250686	2.16E-01
Serum Iron	PD	103	0.01340188	0.02810826	6.34E-01
PD	TIBC	28	0.02610677	0.04424114	5.55E-01
TIBC	PD	148	0.08108744	0.03332949	1.50E-02
PD	TSAT	28	-0.0263594	0.0630436	6.76E-01
TSAT	PD	120	-0.0191989	0.03161092	5.44E-01
PD	PVS	59	-0.0525885	0.07009492	4.53E-01
PVS	PD	22	-0.0230366	0.03994543	5.64E-01

Supplementary Table 5 Mendelian Randomization results using GSMR. GWAS summary statistics of serum iron, transferrin saturation (TSAT), total iron binding capacity (TIBC), ferritin(30), *and parkinson's disease*(52) were taken from previously published studies.

Diagnosis	Controls	Cases	IPW for Cases	OR	CI_lower	CI_upper	Z	P
**Abnormalities of Gait and Mobility [R26]**	35077	206	3.63	0.8881	0.8274	0.9533	-3.2846	0.001
**Other degenerative diseases of Nervous System (including Alzheimer's) [G30-32]**	35223	60	5.70	1.0823	0.9754	1.201	1.4901	0.1362
**Parkinson's Disease [G20]**	35219	64	3.89	0.7356	0.6519	0.8302	-4.978	6.42E-07
**Essential Tremor [G25]**	35201	82	1.95	1.0334	0.8865	1.2048	0.4201	0.6744
**Movement Disorders [G20-26]**	35116	167	2.65	0.9253	0.8437	1.0148	-1.6487	0.0992

Supplementary Table 6 Association results from regression in Subsample B of PolyVoxel Score (PVS) with each neurology disorder or collection of disorders.

	OR	*z*	*P>|z|*
1st Quantile	*3.276*	*5.724*	*$1.04x10^{-8}$*
2nd Quantile	*2.606*	*4.417*	*$1.00x10^{-5}$*
3rd Quantile	*1.801*	*2.567*	*$1.03x10^{-2}$*
C282Y Homozygosity	*2.470*	*3.445*	*$5.72x10^{-4}$*

Supplementary Table 7 Weighted Quantile Regression to predict PD status. Results from regression in subsample C with single categorical variable indicating PVS quantile or C282Y homozygosity status. The reference group was the 4th PVS quartile group. PD cases in the imaging cohort were upweighted with a frequency of 3.89 to correct for PD case depletion within neuroimaging (see Supplementary Table 1), all other observations were given a weighting of 1.

Data S1 ***Extended Data Tables****: Results from GWAS analysis and associated enrichment analysis.*

REFERENCES AND NOTES

1. T. A. Rouault, Iron metabolism in the CNS: Implications for neurodegenerative diseases. *Nat. Rev. Neurosci.* **14**, 551–564 (2013).

2. S.-R. Pasricha, J. Tye-Din, M. U. Muckenthaler, D. W. Swinkels, Iron deficiency. *Lancet* **397**, 233–248 (2021).

3. M. K. Georgieff, Long-term brain and behavioral consequences of early iron deficiency: Nutrition reviews. *Nutr. Rev.* **69**, S43–S48 (2011).

4. B. Do Van, F. Gouel, A. Jonneaux, K. Timmerman, P. Gelé, M. Pétrault, M. Bastide, C. Laloux, C. Moreau, R. Bordet, D. Devos, J.-C. Devedjian, Ferroptosis, a newly characterized form of cell death in Parkinson's disease that is regulated by PKC. *Neurobiol. Dis.* **94**, 169–178 (2016).

5. J. Li, F. Cao, H. Yin, Z. Huang, Z. Lin, N. Mao, B. Sun, G. Wang, Ferroptosis: Past, present and future. *Cell Death Dis.* **11**, 88 (2020).

6. L. W. Powell, R. C. Seckington, Y. Deugnier, Haemochromatosis. *Lancet* **388**, 706–716 (2016).

7. E. Nemeth, T. Ganz, Hepcidin-ferroportin interaction controls systemic iron homeostasis. *Int. J. Mol. Sci.* **22**, 6493 (2021).

8. M. W. Hentze, M. U. Muckenthaler, B. Galy, C. Camaschella, Two to tango: Regulation of mammalian iron metabolism. *Cell* **142**, 24–38 (2010).

9. S. Levi, M. Ripamonti, A. S. Moro, A. Cozzi, Iron imbalance in neurodegeneration. *Mol. Psychiatry* **29**, 1139–1152 (2024).

10. J. C. Wood, C. Enriquez, N. Ghugre, J. M. Tyzka, S. Carson, M. D. Nelson, T. D. Coates, MRI R2 and R2* mapping accurately estimates hepatic iron concentration in transfusion-dependent thalassemia and sickle cell disease patients. *Blood* **106**, 1460–1465 (2005).

11. D. Aquino, A. Bizzi, M. Grisoli, B. Garavaglia, M. G. Bruzzone, N. Nardocci, M. Savoiardo, L. Chiapparini, Age-related iron deposition in the basal ganglia: Quantitative analysis in healthy subjects. *Radiology* **252**, 165–172 (2009).

12. S. Ayton, P. Lei, Nigral iron elevation is an invariable feature of Parkinson's disease and is a sufficient cause of neurodegeneration. *Biomed. Res. Int.* **2014**, 1–9 (2014).

13. S. J. Hayflick, M. A. Kurian, P. Hogarth, "Neurodegeneration with brain iron accumulation" in *Neurogenetics, Part I*, D. H. Geschwind, H. L. Paulson, C. Klein, Eds. (Elsevier, 2018), vol. 147, pp. 293–305.

14. D. Kulshreshtha, J. Ganguly, M. Jog, Iron chelation in movement disorders: Logical or ironical. *Can. J. Neurol. Sci.* **48**, 752–759 (2021).

15. T. Klopstock, F. Tricta, L. Neumayr, I. Karin, G. Zorzi, C. Fradette, T. Kmieć, B. Büchner, H. E. Steele, R. Horvath, P. F. Chinnery, A. Basu, C. Küpper, C. Neuhofer, B. Kálmán, P. Dušek, Z. Yapici, I. Wilson, F. Zhao, F. Zibordi, N. Nardocci, C. Aguilar, S. J. Hayflick, M. Spino, A. M. Blamire, P. Hogarth, E. Vichinsky, Safety and efficacy of deferiprone for pantothenate kinase-associated neurodegeneration: A randomised, double-blind, controlled trial and an open-label extension study. *Lancet Neurol.* **18**, 631–642 (2019).

16. D. Devos, J. Labreuche, O. Rascol, J.-C. Corvol, A. Duhamel, P. Guyon Delannoy, W. Poewe, Y. Compta, N. Pavese, E. Růžička, P. Dušek, B. Post, B. R. Bloem, D. Berg, W. Maetzler, M. Otto, M.-O. Habert, S. Lehericy, J. Ferreira, R. Dodel, C. Tranchant, A. Eusebio, S. Thobois, A.-R. Marques, W. G. Meissner, F. Ory-Magne, U. Walter, R. M. A. de Bie, M. Gago, D. Vilas, J. Kulisevsky, C. Januario, M. V. S. Coelho, S. Behnke, P. Worth, K. Seppi, T. Ouk, C. Potey, C. Leclercq, R. Viard, G. Kuchcinski, R. Lopes, J.-P. Pruvo, P. Pigny, G. Garçon, O. Simonin, J. Carpentier, A.-S. Rolland, D. Nyholm, C. Scherfler, J.-F. Mangin, M. Chupin, R. Bordet, D. T. Dexter, C. Fradette, M. Spino, F. Tricta, S. Ayton, A. I. Bush, J.-C. Devedjian, J. A. Duce, I. Cabantchik, L. Defebvre, D. Deplanque, C. Moreau, Trial of deferiprone in Parkinson's disease. *N. Engl. J. Med.* **387**, 2045–2055 (2022).

17. F. Marchand, C. Moreau, G. Kuchcinski, V. Huin, L. Defebvre, D. Devos, Conservative iron chelation for neuroferritinopathy. *Mov. Disord.* **37**, 1948–1952 (2022).

18. C. Wang, A. B. Martins-Bach, F. Alfaro-Almagro, G. Douaud, J. C. Klein, A. Llera, C. Fiscone, R. Bowtell, L. T. Elliott, S. M. Smith, B. C. Tendler, K. L. Miller, Phenotypic and genetic associations of quantitative magnetic susceptibility in UK Biobank brain imaging. *Nat. Neurosci.* **25**, 818–831 (2022).

19. J. P. Lerch, A. J. W. Van Der Kouwe, A. Raznahan, T. Paus, H. Johansen-Berg, K. L. Miller, S. M. Smith, B. Fischl, S. N. Sotiropoulos, Studying neuroanatomy using MRI. *Nat. Neurosci.* **20**, 314–326 (2017).

20. T. Ganz, E. Nemeth, Hepcidin and iron homeostasis. *Biochim. Biophys. Acta* **1823**, 1434–1443 (2012).

21. L. M. Fletcher, J. W. Halliday, Haemochromatosis: Understanding the mechanism of disease and implications for diagnosis and patient management following the recent cloning of novel genes involved in iron metabolism. *J. Intern. Med.* **251**, 181–192 (2002).

22. A. Bomford, Genetics of haemochromatosis. *Lancet* **360**, 1673–1681 (2002).

23. L. C. Pilling, J. Tamosauskaite, G. Jones, A. R. Wood, L. Jones, C. L. Kuo, G. A. Kuchel, L. Ferrucci, D. Melzer, Common conditions associated with hereditary haemochromatosis genetic variants: Cohort study in UK Biobank. *The BMJ* **364**, k5222 (2019).

24. J. N. Feder, A. Gnirke, W. Thomas, Z. Tsuchihashi, D. A. Ruddy, A. Basava, F. Dormishian, R. Domingo Jr., M. C. Ellis, A. Fullan, L. M. Hinton, N. L. Jones, B. E. Kimmel, G. S. Kronmal, P. Lauer, V. K. Lee, D. B. Loeb, F. A. Mapa, E. McClelland, N. C. Meyer, G. A. Mintier, N. Moeller, T. Moore, E. Morikang, C. E. Prass, L. Quintana, S. M. Starnes, R. C. Schatzman, K. J. Brunke, D. T. Drayna, N. J. Risch, B. R. Bacon, R. K. Wolff, A novel MHC class I-like gene is mutated in patients with hereditary haemochromatosis. *Nat. Genet.* **13**, 399–408 (1996).

25. R. Loughnan, J. Ahern, C. Tompkins, C. E. Palmer, J. Iversen, W. K. Thompson, O. Andreassen, T. Jernigan, L. Sugrue, A. Dale, M. E. T. Boyle, C. C. Fan, Association of genetic variant linked to hemochromatosis with brain magnetic resonance imaging measures of iron and movement disorders. *JAMA Neurol.* **79**, 919–928 (2022).

26. R. J. Loughnan, A. A. Shadrin, O. Frei, D. van der Meer, W. Zhao, C. E. Palmer, W. K. Thompson, C. Makowski, T. L. Jernigan, O. A. Andreassen, C. C. Fan, A. M. Dale, Generalization of cortical MOSTest genome-wide associations within and across samples. *Neuroimage* **263**, 119632 (2022).

27. C. Beaumont, J. Delaunay, G. Hetet, B. Grandchamp, M. de Montalembert, G. Tchernia, Two new human DMT1 gene mutations in a patient with microcytic anemia, low ferritinemia, and liver iron overload. *Blood* **107**, 4168–4170 (2006).

28. E. Bardou-Jacquet, M.-L. Island, A.-M. Jouanolle, L. Détivaud, N. Fatih, M. Ropert, E. Brissot, A. Mosser, H. Maisonneuve, P. Brissot, O. Loréal, A novel N491S mutation in the human SLC11A2 gene impairs protein trafficking and in association with the G212V mutation leads to microcytic anemia and liver iron overload. *Blood Cells Mol. Dis.* **47**, 243–248 (2011).

29. J. R. Connor, S. L. Menzies, S. M. S. Martin, E. J. Mufson, A histochemical study of iron, transferrin, and ferritin in Alzheimer's diseased brains. *J. Neurosci. Res.* **31**, 75–83 (1992).

30. S. Bell, A. S. Rigas, M. K. Magnusson, E. Ferkingstad, E. Allara, G. Bjornsdottir, A. Ramond, E. Sørensen, G. H. Halldorsson, D. S. Paul, K. S. Burgdorf, H. P. Eggertsson, J. M. M. Howson, L. W. Thørner, S. Kristmundsdottir, W. J. Astle, C. Erikstrup, J. K. Sigurdsson, D. Vuckovic, K. M. Dinh, V. Tragante, P. Surendran, O. B. Pedersen, B. Vidarsson, T. Jiang, H. M. Paarup, P. T. Onundarson, P. Akbari, K. R. Nielsen, S. H. Lund, K. Juliusson, M. I. Magnusson, M. L. Frigge, A. Oddsson, I. Olafsson, S. Kaptoge, H. Hjalgrim, G. Runarsson, A. M. Wood, I. Jonsdottir, T. F. Hansen, O. Sigurdardottir, H. Stefansson, D. Rye, S. Andersen, K. Burgdorf, G. Jemec, P. Jennum, P. Johansson, K. R. Nielsen, M. Nyegaard, M. Petersen, T. Werge, H. Stefánsson, U. Thorsteinsdóttir, J. E. Peters, D. Westergaard, H. Holm, N. Soranzo, K. Banasik, G. Thorleifsson, W. H. Ouwehand, U. Thorsteinsdottir, D. J. Roberts, P. Sulem, A. S. Butterworth, D. F. Gudbjartsson, J. Danesh, S. Brunak, E. Di Angelantonio, H. Ullum, K. Stefansson, A genome-wide meta-analysis yields 46 new loci associating with biomarkers of iron homeostasis. *Commun. Biol.* **4**, 156 (2021).

31. K. J. McMillan, M. Gallon, A. P. Jellett, T. Clairfeuille, F. C. Tilley, I. McGough, C. M. Danson, K. J. Heesom, K. A. Wilkinson, B. M. Collins, P. J. Cullen, Atypical parkinsonism–

associated retromer mutant alters endosomal sorting of specific cargo proteins. *J. Cell Biol.* **214**, 389–399 (2016).

32. J. Petrak, D. Vyoral, Hephaestin—A ferroxidase of cellular iron export. *Int. J. Biochem. Cell Biol.* **37**, 1173–1178 (2005).

33. O. B. Smeland, A. Shadrin, S. Bahrami, I. Broce, M. Tesli, O. Frei, K. V. Wirgenes, K. S. O'Connell, F. Krull, F. Bettella, N. E. Steen, L. Sugrue, Y. Wang, P. Svenningsson, M. Sharma, L. Pihlstrøm, M. Toft, M. O'Donovan, S. Djurovic, R. Desikan, A. M. Dale, O. A. Andreassen, Genome-wide association analysis of Parkinson's disease and schizophrenia reveals shared genetic architecture and identifies novel risk loci. *Biol. Psychiatry* **89**, 227–235 (2021).

34. B. Bulik-Sullivan, P. R. Loh, H. K. Finucane, S. Ripke, J. Yang, N. Patterson, M. J. Daly, A. L. Price, B. M. Neale, A. Corvin, J. T. R. Walters, K. H. Farh, P. A. Holmans, P. Lee, D. A. Collier, H. Huang, T. H. Pers, I. Agartz, E. Agerbo, M. Albus, M. Alexander, F. Amin, S. A. Bacanu, M. Begemann, R. A. Belliveau, J. Bene, S. E. Bergen, E. Bevilacqua, T. B. Bigdeli, D. W. Black, R. Bruggeman, N. G. Buccola, R. L. Buckner, W. Byerley, W. Cahn, G. Cai, M. J. Cairns, D. Campion, R. M. Cantor, V. J. Carr, N. Carrera, S. V. Catts, K. D. Chambert, R. C. K. Chan, R. Y. L. Chen, E. Y. H. Chen, W. Cheng, E. F. C. Cheung, S. A. Chong, C. R. Cloninger, D. Cohen, N. Cohen, P. Cormican, N. Craddock, B. Crespo-Facorro, J. J. Crowley, D. Curtis, M. Davidson, K. L. Davis, F. Degenhardt, J. Del Favero, L. E. DeLisi, D. Demontis, D. Dikeos, T. Dinan, S. Djurovic, G. Donohoe, E. Drapeau, J. Duan, F. Dudbridge, N. Durmishi, P. Eichhammer, J. Eriksson, V. Escott-Price, L. Essioux, A. H. Fanous, M. S. Farrell, J. Frank, L. Franke, R. Freedman, N. B. Freimer, M. Friedl, J. I. Friedman, M. Fromer, G. Genovese, L. Georgieva, E. S. Gershon, I. Giegling, P. Giusti-Rodríguez, S. Godard, J. I. Goldstein, V. Golimbet, S. Gopal, J. Gratten, L. De Haan, C. Hammer, M. L. Hamshere, M. Hansen, T. Hansen, V. Haroutunian, A. M. Hartmann, F. A. Henskens, S. Herms, J. N. Hirschhorn, P. Hoffmann, A. Hofman, M. V. Hollegaard, D. M. Hougaard, M. Ikeda, I. Joa, A. Juliá, R. S. Kahn, L. Kalaydjieva, S. Karachanak-Yankova, J. Karjalainen, D. Kavanagh, M. C. Keller, B. J. Kelly, J. L. Kennedy, A. Khrunin, Y. Kim, J. Klovins, J. A. Knowles, B. Konte, V. Kucinskas, Z. A. Kucinskiene, H. Kuzelova-Ptackova, A. K. Kähler, C. Laurent, J. L. C. Keong, S. H. Lee, S. E. Legge, B. Lerer, M. Li, T. Li, K. Y. Liang, J.

Lieberman, S. Limborska, C. M. Loughland, J. Lubinski, J. Lönnqvist, M. Macek, P. K. E. Magnusson, B. S. Maher, W. Maier, J. Mallet, S. Marsal, M. Mattheisen, M. Mattingsdal, R. W. McCarley, C. McDonald, A. M. McIntosh, S. Meier, C. J. Meijer, B. Melegh, I. Melle, R. I. Mesholam-Gately, A. Metspalu, P. T. Michie, L. Milani, V. Milanova, Y. Mokrab, D. W. Morris, O. Mors, K. C. Murphy, R. M. Murray, I. Myin-Germeys, B. Müller-Myhsok, M. Nelis, I. Nenadic, D. A. Nertney, G. Nestadt, K. K. Nicodemus, L. Nikitina-Zake, L. Nisenbaum, A. Nordin, E. O'Callaghan, C. O'Dushlaine, F. A. O'Neill, S. Y. Oh, A. Olincy, L. Olsen, J. Van Os, C. Pantelis, G. N. Papadimitriou, S. Papiol, E. Parkhomenko, M. T. Pato, T. Paunio, M. Pejovic-Milovancevic, D. O. Perkins, O. Pietiläinen, J. Pimm, A. J. Pocklington, J. Powell, A. E. Pulver, S. M. Purcell, D. Quested, H. B. Rasmussen, A. Reichenberg, M. A. Reimers, A. L. Richards, J. L. Roffman, P. Roussos, D. M. Ruderfer, V. Salomaa, A. R. Sanders, U. Schall, C. R. Schubert, T. G. Schulze, S. G. Schwab, E. M. Scolnick, R. J. Scott, L. J. Seidman, J. Shi, E. Sigurdsson, T. Silagadze, J. M. Silverman, K. Sim, P. Slominsky, J. W. Smoller, H. C. So, C. C. A. Spencer, E. A. Stahl, H. Stefansson, S. Steinberg, E. Stogmann, R. E. Straub, E. Strengman, J. Strohmaier, T. S. Stroup, M. Subramaniam, J. Suvisaari, D. M. Svrakic, J. P. Szatkiewicz, E. Söderman, S. Thirumalai, D. Toncheva, P. A. Tooney, S. Tosato, J. Veijola, J. Waddington, D. Walsh, D. Wang, Q. Wang, B. T. Webb, M. Weiser, D. B. Wildenauer, N. M. Williams, S. Williams, S. H. Witt, A. R. Wolen, E. H. M. Wong, B. K. Wormley, J. Q. Wu, H. S. Xi, C. C. Zai, X. Zheng, F. Zimprich, N. R. Wray, K. Stefansson, P. M. Visscher, R. Adolfsson, O. A. Andreassen, D. H. R. Blackwood, E. Bramon, J. D. Buxbaum, A. D. Børglum, S. Cichon, A. Darvasi, E. Domenici, H. Ehrenreich, T. Esko, P. V. Gejman, M. Gill, H. Gurling, C. M. Hultman, N. Iwata, A. V. Jablensky, E. G. Jönsson, K. S. Kendler, G. Kirov, J. Knight, T. Lencz, D. F. Levinson, Q. S. Li, J. Liu, A. K. Malhotra, S. A. McCarroll, A. McQuillin, J. L. Moran, P. B. Mortensen, B. J. Mowry, M. M. Nöthen, R. A. Ophoff, M. J. Owen, A. Palotie, C. N. Pato, T. L. Petryshen, D. Posthuma, M. Rietschel, B. P. Riley, D. Rujescu, P. C. Sham, P. Sklar, D. S. Clair, D. R. Weinberger, J. R. Wendland, T. Werge, P. F. Sullivan, M. C. O'Donovan, LD score regression distinguishes confounding from polygenicity in genome-wide association studies. *Nat. Genet.* **47**, 291–295 (2015).

35. H. K. Finucane, Y. A. Reshef, V. Anttila, K. Slowikowski, A. Gusev, A. Byrnes, S. Gazal, P. Loh, C. Lareau, G. Genovese, A. Saunders, E. Macosko, S. Pollack, B. Consortium, J. R. B.

Perry, J. D. Buenrostro, E. Bradley, Heritability enrichment of specifically expressed genes identifies disease-relevant tissues and cell types. *Nat. Genet.* **50**, 621–629 (2018).

36. K. Watanabe, E. Taskesen, A. van Bochoven, D. Posthuma, Functional mapping and annotation of genetic associations with FUMA. *Nat. Commun.* **8**, 1826 (2017).

37. D. Wang, S. Liu, J. Warrell, H. Won, X. Shi, F. C. P. Navarro, D. Clarke, M. Gu, P. Emani, Y. T. Yang, M. Xu, M. J. Gandal, S. Lou, J. Zhang, J. J. Park, C. Yan, S. K. Rhie, K. Manakongtreecheep, H. Zhou, A. Nathan, M. Peters, E. Mattei, D. Fitzgerald, T. Brunetti, J. Moore, Y. Jiang, K. Girdhar, G. E. Hoffman, S. Kalayci, Z. H. Gümü, G. E. Crawford, P. Consortium, P. Roussos, S. Akbarian, A. E. Jaffe, K. P. White, Z. Weng, N. Sestan, D. H. Geschwind, J. A. Knowles, M. B. Gerstein, Comprehensive functional genomic resource and integrative model for the human brain. *Science* **362**, eaat8464 (2018).

38. A. Reinert, M. Morawski, J. Seeger, T. Arendt, T. Reinert, Iron concentrations in neurons and glial cells with estimates on ferritin concentrations. *BMC Neurosci.* **20**, 25 (2019).

39. Z. Zhu, Z. Zheng, F. Zhang, Y. Wu, M. Trzaskowski, R. Maier, M. R. Robinson, J. J. McGrath, P. M. Visscher, N. R. Wray, J. Yang, Causal associations between risk factors and common diseases inferred from GWAS summary data. *Nat. Commun.* **9**, 224 (2018).

40. A. J. Ramsey, P. J. Hillas, P. F. Fitzpatrick, Characterization of the active site iron in tyrosine hydroxylase: Redox states of the iron. *J. Biol. Chem.* **271**, 24395–24400 (1996).

41. P. R. Angelova, M. L. Choi, A. V. Berezhnov, M. H. Horrocks, C. D. Hughes, S. De, M. Rodrigues, R. Yapom, D. Little, K. S. Dolt, T. Kunath, M. J. Devine, P. Gissen, M. S. Shchepinov, S. Sylantyev, E. V. Pavlov, D. Klenerman, A. Y. Abramov, S. Gandhi, Alpha synuclein aggregation drives ferroptosis: An interplay of iron, calcium and lipid peroxidation. *Cell Death Differ.* **27**, 2781–2796 (2020).

42. J.-Y. Wang, Q.-Q. Zhuang, L.-B. Zhu, H. Zhu, T. Li, R. Li, S.-F. Chen, C.-P. Huang, X. Zhang, J.-H. Zhu, Meta-analysis of brain iron levels of Parkinson's disease patients determined by postmortem and MRI measurements. *Sci. Rep.* **6**, 36669 (2016).

43. R. J. Uitti, A. H. Rajput, B. Rozdilsky, M. Bickis, T. Wollin, W. K. Yuen, Regional metal concentrations in Parkinson's disease, other chronic neurological diseases, and control brains. *Can. J. Neurol. Sci.* **16**, 310–314 (1989).

44. D. T. Dexter, F. R. Wells, A. J. Lee, F. Agid, Y. Agid, P. Jenner, C. D. Marsden, Increased nigral iron content and alterations in other metal ions occurring in brain in Parkinson's disease. *J. Neurochem.* **52**, 1830–1836 (1989).

45. R. Savica, B. R. Grossardt, J. M. Carlin, M. Icen, J. H. Bower, J. E. Ahlskog, D. M. Maraganore, D. P. Steensma, W. A. Rocca, Anemia or low hemoglobin levels preceding Parkinson disease. *Neurology* **73**, 1381 (2009).

46. G. Logroscino, H. Chen, A. Wing, A. Ascherio, Blood donations, iron stores, and risk of Parkinson's disease. *Mov. Disord.* **21**, 835–838 (2006).

47. W. Thomas Thach, A. J. Bastian, "Role of the cerebellum in the control and adaptation of gait in health and disease" in *Progress in Brain Research* (Elsevier, 2004), vol. 143, pp. 353–366; https://linkinghub.elsevier.com/retrieve/pii/S0079612303430343.

48. J. M. Hausdorff, M. E. Cudkowicz, R. Firtion, J. Y. Wei, A. L. Goldberger, Gait variability and basal ganglia disorders: Stride-to-stride variations of gait cycle timing in Parkinson's disease and Huntington's disease. *Mov. Disord.* **13**, 428–437 (1998).

49. M. Kafri, E. Sasson, Y. Assaf, Y. Balash, O. Aiznstein, J. M. Hausdorff, N. Giladi, High-level gait disorder: Associations with specific white matter changes observed on advanced diffusion imaging. *J. Neuroimaging* **23**, 39–46 (2013).

50. A. Demain, G. W. M. Westby, S. Fernandez-Vidal, C. Karachi, F. Bonneville, M. C. Do, C. Delmaire, D. Dormont, E. Bardinet, Y. Agid, N. Chastan, M.-L. Welter, High-level gait and balance disorders in the elderly: A midbrain disease? *J. Neurol.* **261**, 196–206 (2014).

51. W. K. Al-Delaimy, E. H. J. M. Jansen, P. H. M. Peeters, J. D. Van Der Laan, P. A. H. Van Noord, H. C. Boshuizen, Y. T. Van Der Schouw, M. Jenab, P. Ferrari, H. B. Bueno-de-

Mesquita, Reliability of biomarkers of iron status, blood lipids, oxidative stress, vitamin D, C-reactive protein and fructosamine in two Dutch cohorts. *Biomarkers* **11**, 370–382 (2006).

52. M. A. Nalls, C. Blauwendraat, C. L. Vallerga, K. Heilbron, S. Bandres-Ciga, D. Chang, M. Tan, D. A. Kia, A. J. Noyce, A. Xue, J. Bras, E. Young, R. von Coelln, J. Simón-Sánchez, C. Schulte, M. Sharma, L. Krohn, L. Pihlstrøm, A. Siitonen, H. Iwaki, H. Leonard, F. Faghri, J. R. Gibbs, D. G. Hernandez, S. W. Scholz, J. A. Botia, M. Martinez, J. C. Corvol, S. Lesage, J. Jankovic, L. M. Shulman, M. Sutherland, P. Tienari, K. Majamaa, M. Toft, O. A. Andreassen, T. Bangale, A. Brice, J. Yang, Z. Gan-Or, T. Gasser, P. Heutink, J. M. Shulman, N. W. Wood, D. A. Hinds, J. A. Hardy, H. R. Morris, J. Gratten, P. M. Visscher, R. R. Graham, A. B. Singleton; 23andMe Research Team; System Genomics of Parkinson's Disease Consortium; International Parkinson's Disease Genomics Consortium, Identification of novel risk loci, causal insights, and heritable risk for Parkinson's disease: A meta-analysis of genome-wide association studies. *Lancet Neurol.* **18**, 1091–1102 (2019).

53. J. Flint, K. S. Kendler, The genetics of major depression. *Neuron* **81**, 484–503 (2014).

54. A. Navarro-Romero, M. Montpeyó, M. Martinez-Vicente, The emerging role of the lysosome in Parkinson's disease. *Cells* **9**, 2399 (2020).

55. J. Ahern, M. E. Boyle, W. K. Thompson, C. C. Fan, R. Loughnan, Dietary and lifestyle factors of brain iron accumulation and Parkinson's disease risk. medRxiv 24304253 [Preprint] (2024); https://doi.org/10.1101/2024.03.13.24304253.

56. J. R. Connor, S. L. Menzies, Relationship of iron to oligodendrocytes and myelination. *Glia* **17**, 83–93 (1996).

57. S. Jäkel, L. Dimou, Glial cells and their function in the adult brain: A journey through the history of their ablation. *Front. Cell. Neurosci.* **11**, 24 (2017).

58. I. Friedrich, K. Reimann, S. Jankuhn, E. Kirilina, J. Stieler, M. Sonntag, J. Meijer, N. Weiskopf, T. Reinert, T. Arendt, M. Morawski, Cell specific quantitative iron mapping on brain slices by immuno-µPIXE in healthy elderly and Parkinson's disease. *Acta Neuropathol. Commun.* **9**, 47 (2021).

59. S. K. Ryan, M. Zelic, Y. Han, E. Teeple, L. Chen, M. Sadeghi, S. Shankara, L. Guo, C. Li, F. Pontarelli, E. H. Jensen, A. L. Comer, D. Kumar, M. Zhang, J. Gans, B. Zhang, J. D. Proto, J. Saleh, J. C. Dodge, V. Savova, D. Rajpal, D. Ofengeim, T. R. Hammond, Microglia ferroptosis is regulated by SEC24B and contributes to neurodegeneration. *Nat. Neurosci.* **26**, 12–26 (2023).

60. Y. Muñoz, C. M. Carrasco, J. D. Campos, P. Aguirre, M. T. Núñez, Parkinson's disease: The mitochondria-iron link. *Parkinsons Dis.* **2016**, 7049108 (2016).

61. A. E. Oakley, J. F. Collingwood, J. Dobson, G. Love, H. R. Perrott, J. A. Edwardson, M. Elstner, C. M. Morris, Individual dopaminergic neurons show raised iron levels in Parkinson disease. *Neurology* **68**, 1820–1825 (2007).

62. W. Poewe, K. Seppi, C. M. Tanner, G. M. Halliday, P. Brundin, J. Volkmann, A.-E. Schrag, A. E. Lang, Parkinson disease. *Nat. Rev. Dis. Primer* **3**, 17013 (2017).

63. R. Camicioli, M. M. Moore, A. Kinney, E. Corbridge, K. Glassberg, J. A. Kaye, Parkinson's disease is associated with hippocampal atrophy. *Mov. Disord.* **18**, 784–790 (2003).

64. B. Larsen, V. Olafsson, F. Calabro, C. Laymon, B. Tervo-Clemmens, E. Campbell, D. Minhas, D. Montez, J. Price, B. Luna, Maturation of the human striatal dopamine system revealed by PET and quantitative MRI. *Nat. Commun.* **11**, 846 (2020).

65. S. Lim, C. E. Han, P. J. Uhlhaas, M. Kaiser, Preferential detachment during human brain development: Age- and sex-specific structural connectivity in diffusion tensor imaging (DTI) data. *Cereb. Cortex* **25**, 1477–1489 (2015).

66. R. M. Wolf, D. Long, Pubertal development. *Pediatr. Rev.* **37**, 292–300 (2016).

67. R. J. Ordidge, J. M. Gorell, J. C. Deniau, R. A. Knight, J. A. Helpern, Assessment of relative brain iron concentrations using T2-weighted and T2*-weighted MRI at 3 Tesla. *Magn. Reson. Med.* **32**, 335–341 (1994).

68. R. Prayson, *Neuropathology* (2011).

69. C. Grochowski, E. Blicharska, J. Baj, A. Mierzwińska, K. Brzozowska, A. Forma, R. Maciejewski, Serum iron, magnesium, copper, and manganese levels in alcoholism: A systematic review. *Molecules* **24**, 1361 (Elsevier Health Sciences; 2019).

70. B. Henninger, J. Alustiza, M. Garbowski, Y. Gandon, Practical guide to quantification of hepatic iron with MRI. *Eur. Radiol.* **30**, 383–393 (2020).

71. E. Von Elm, D. G. Altman, M. Egger, S. J. Pocock, P. C. Gøtzsche, J. P. Vandenbroucke, The strengthening the reporting of observational studies in epidemiology (STROBE) statement: Guidelines for reporting observational studies. *Epidemiology* **18**, 800–804 (2007).

72. F. Alfaro-Almagro, M. Jenkinson, N. K. Bangerter, J. L. R. Andersson, L. Griffanti, G. Douaud, S. N. Sotiropoulos, S. Jbabdi, M. Hernandez-Fernandez, E. Vallee, D. Vidaurre, M. Webster, P. McCarthy, C. Rorden, A. Daducci, D. C. Alexander, H. Zhang, I. Dragonu, P. M. Matthews, K. L. Miller, S. M. Smith, Image processing and Quality Control for the first 10,000 brain imaging datasets from UK Biobank. *Neuroimage* **166**, 400–424 (2018).

73. S. Das, L. Forer, S. Schönherr, C. Sidore, A. E. Locke, A. Kwong, S. I. Vrieze, E. Y. Chew, S. Levy, M. McGue, D. Schlessinger, D. Stambolian, P.-R. Loh, W. G. Iacono, A. Swaroop, L. J. Scott, F. Cucca, F. Kronenberg, M. Boehnke, G. R. Abecasis, C. Fuchsberger, Next-generation genotype imputation service and methods. *Nat. Genet.* **48**, 1284–1287 (2016).

74. D. Taliun, D. N. Harris, M. D. Kessler, J. Carlson, Z. A. Szpiech, R. Torres, S. A. G. Taliun, A. Corvelo, S. M. Gogarten, H. M. Kang, A. N. Pitsillides, J. LeFaive, S. Lee, X. Tian, B. L. Browning, S. Das, A. K. Emde, W. E. Clarke, D. P. Loesch, A. C. Shetty, T. W. Blackwell, A. V. Smith, Q. Wong, X. Liu, M. P. Conomos, D. M. Bobo, F. Aguet, C. Albert, A. Alonso, K. G. Ardlie, D. E. Arking, S. Aslibekyan, P. L. Auer, J. Barnard, R. G. Barr, L. Barwick, L. C. Becker, R. L. Beer, E. J. Benjamin, L. F. Bielak, J. Blangero, M. Boehnke, D. W. Bowden, J. A. Brody, E. G. Burchard, B. E. Cade, J. F. Casella, B. Chalazan, D. I. Chasman, Y. D. I. Chen, M. H. Cho, S. H. Choi, M. K. Chung, C. B. Clish, A. Correa, J. E. Curran, B. Custer, D. Darbar, M. Daya, M. de Andrade, D. L. DeMeo, S. K. Dutcher, P. T. Ellinor, L. S. Emery, C. Eng, D. Fatkin, T. Fingerlin, L. Forer, M. Fornage, N. Franceschini, C. Fuchsberger, S. M. Fullerton, S. Germer, M. T. Gladwin, D. J. Gottlieb, X. Guo, M. E. Hall, J. He, N. L. Heard-Costa, S. R. Heckbert, M. R. Irvin, J. M. Johnsen, A. D. Johnson, R. Kaplan, S. L. R. Kardia,

T. Kelly, S. Kelly, E. E. Kenny, D. P. Kiel, R. Klemmer, B. A. Konkle, C. Kooperberg, A. Köttgen, L. A. Lange, J. Lasky-Su, D. Levy, X. Lin, K. H. Lin, C. Liu, R. J. F. Loos, L. Garman, R. Gerszten, S. A. Lubitz, K. L. Lunetta, A. C. Y. Mak, A. Manichaikul, A. K. Manning, R. A. Mathias, D. D. McManus, S. T. McGarvey, J. B. Meigs, D. A. Meyers, J. L. Mikulla, M. A. Minear, B. D. Mitchell, S. Mohanty, M. E. Montasser, C. Montgomery, A. C. Morrison, J. M. Murabito, A. Natale, P. Natarajan, S. C. Nelson, K. E. North, J. R. O'Connell, N. D. Palmer, N. Pankratz, G. M. Peloso, P. A. Peyser, J. Pleiness, W. S. Post, B. M. Psaty, D. C. Rao, S. Redline, A. P. Reiner, D. Roden, J. I. Rotter, I. Ruczinski, C. Sarnowski, S. Schoenherr, D. A. Schwartz, J. S. Seo, S. Seshadri, V. A. Sheehan, W. H. Sheu, M. B. Shoemaker, N. L. Smith, J. A. Smith, N. Sotoodehnia, A. M. Stilp, W. Tang, K. D. Taylor, M. Telen, T. A. Thornton, R. P. Tracy, D. J. Van Den Berg, R. S. Vasan, K. A. Viaud-Martinez, S. Vrieze, D. E. Weeks, B. S. Weir, S. T. Weiss, L. C. Weng, C. J. Willer, Y. Zhang, X. Zhao, D. K. Arnett, A. E. Ashley-Koch, K. C. Barnes, E. Boerwinkle, S. Gabriel, R. Gibbs, K. M. Rice, S. S. Rich, E. K. Silverman, P. Qasba, W. Gan; NHLBI Trans-Omics for Precision Medicine (TOPMed) Consortium, G. J. Papanicolaou, D. A. Nickerson, S. R. Browning, M. C. Zody, S. Zöllner, J. G. Wilson, L. A. Cupples, C. C. Laurie, C. E. Jaquish, R. D. Hernandez, T. D. O'Connor, G. R. Abecasis, Sequencing of 53,831 diverse genomes from the NHLBI TOPMed Program. *Nature* **590**, 290–299 (2021).

75. C. Fuchsberger, G. R. Abecasis, D. A. Hinds, minimac2: Faster genotype imputation. *Bioinformatics* **31**, 782–784 (2015).

76. M. P. Conomos, M. B. Miller, T. A. Thornton, Robust inference of population structure for ancestry prediction and correction of stratification in the presence of relatedness. *Genet. Epidemiol.* **39**, 276–293 (2015).

77. C. C. Fan, R. Loughnan, S. Wilson, J. K. Hewitt; ABCD Genetic Working Group, Genotype data and derived genetic instruments of adolescent brain cognitive development study® for better understanding of human brain development. *Behav. Genet.* **53**, 159–168 (2023).

78. C.-Y. Chen, S. Pollack, D. J. Hunter, J. N. Hirschhorn, P. Kraft, A. L. Price, Improved ancestry inference using weights from external reference panels. *Bioinformatics* **29**, 1399–1406 (2013).

79. The 1000 Genomes Project Consortium, A global reference for human genetic variation. *Nature* **526**, 68–74 (2015).

80. D. Reich, N. Patterson, D. Campbell, A. Tandon, S. Mazieres, N. Ray, M. V. Parra, W. Rojas, C. Duque, N. Mesa, L. F. García, O. Triana, S. Blair, A. Maestre, J. C. Dib, C. M. Bravi, G. Bailliet, D. Corach, T. Hünemeier, M.-C. Bortolini, F. M. Salzano, M. L. Petzl-Erler, V. Acuña-Alonzo, C. Aguilar-Salinas, S. Canizales-Quinteros, T. Tusié-Luna, L. Riba, M. Rodríguez-Cruz, M. Lopez-Alarcón, R. Coral-Vazquez, T. Canto-Cetina, I. Silva-Zolezzi, J. C. Fernandez-Lopez, A. V. Contreras, G. Jimenez-Sanchez, M. J. Gómez-Vázquez, J. Molina, Á. Carracedo, A. Salas, C. Gallo, G. Poletti, D. B. Witonsky, G. Alkorta-Aranburu, R. I. Sukernik, L. Osipova, S. Fedorova, R. Vasquez, M. Villena, C. Moreau, R. Barrantes, D. Pauls, L. Excoffier, G. Bedoya, F. Rothhammer, J. M. Dugoujon, G. Larrouy, W. Klitz, D. Labuda, J. Kidd, K. Kidd, A. D. Rienzo, N. B. Freimer, A. L. Price, A. Ruiz-Linares, Reconstructing native american population history. *Nature* **488**, 370–374 (2012).

81. R. Watts, M. P. Harms, A. M. Dale, M. Daniela Cornejo, J. M. Kuperman, M. E. Soules, D. V. Dellarco, M. H. Mejia, T. D. Wager, M. T. Banich, J. R. Polimeni, K. M. Thomas, D. M. Barch, T. Teslovich, B. J. Casey, H. Bartsch, A. O. Cohen, M. C. Riedel, M. T. Sutherland, E. Earl, D. J. Hagler, C. A. Orr, A. S. Dick, H. Garavan, J. M. Bjork, C. S. Sicat, N. K. Speer, M. M. Heitzeg, B. Chaarani, D. A. Fair, T. Cannonier, M. I. Conley, M. Rosenberg, N. U. F. Dosenbach, The Adolescent Brain Cognitive Development (ABCD) study: Imaging acquisition across 21 sites. *Dev. Cogn. Neurosci.* **32**, 43–54 (2018).

82. D. J. Hagler Jr., S. N. Hatton, M. D. Cornejo, C. Makowski, D. A. Fair, A. S. Dick, M. T. Sutherland, B. J. Casey, D. M. Barch, M. P. Harms, R. Watts, J. M. Bjork, H. P. Garavan, L. Hilmer, C. J. Pung, C. S. Sicat, J. Kuperman, H. Bartsch, F. Xue, M. M. Heitzeg, A. R. Laird, T. T. Trinh, R. Gonzalez, S. F. Tapert, M. C. Riedel, L. M. Squeglia, L. W. Hyde, M. D. Rosenberg, E. A. Earl, K. D. Howlett, F. C. Baker, M. Soules, J. Diaz, O. R. de Leon, W. K. Thompson, M. C. Neale, M. Herting, E. R. Sowell, R. P. Alvarez, S. W. Hawes, M. Sanchez, J. Bodurka, F. J. Breslin, A. S. Morris, M. P. Paulus, W. K. Simmons, J. R. Polimeni, A. van der Kouwe, A. S. Nencka, K. M. Gray, C. Pierpaoli, J. A. Matochik, A. Noronha, W. M. Aklin, K. Conway, M. Glantz, E. Hoffman, R. Little, M. Lopez, V. Pariyadath, S. R. Weiss, D. L. Wolff-Hughes, R. DelCarmen-Wiggins, S. W. F. Ewing, O. Miranda-Dominguez, B. J.

Nagel, A. J. Perrone, D. T. Sturgeon, A. Goldstone, A. Pfefferbaum, K. M. Pohl, D. Prouty, K. Uban, S. Y. Bookheimer, M. Dapretto, A. Galvan, K. Bagot, J. Giedd, M. A. Infante, J. Jacobus, K. Patrick, P. D. Shilling, R. Desikan, Y. Li, L. Sugrue, M. T. Banich, N. Friedman, J. K. Hewitt, C. Hopfer, J. Sakai, J. Tanabe, L. B. Cottler, S. J. Nixon, L. Chang, C. Cloak, T. Ernst, G. Reeves, D. N. Kennedy, S. Heeringa, S. Peltier, J. Schulenberg, C. Sripada, R. A. Zucker, W. G. Iacono, M. Luciana, F. J. Calabro, D. B. Clark, D. A. Lewis, B. Luna, C. Schirda, T. Brima, J. J. Foxe, E. G. Freedman, D. W. Mruzek, M. J. Mason, R. Huber, E. McGlade, A. Prescot, P. F. Renshaw, D. A. Yurgelun-Todd, N. A. Allgaier, J. A. Dumas, M. Ivanova, A. Potter, P. Florsheim, C. Larson, K. Lisdahl, M. E. Charness, B. Fuemmeler, J. M. Hettema, H. H. Maes, J. Steinberg, A. P. Anokhin, P. Glaser, A. C. Heath, P. A. Madden, A. Baskin-Sommers, R. T. Constable, S. J. Grant, G. J. Dowling, S. A. Brown, T. L. Jernigan, A. M. Dale, Image processing and analysis methods for the Adolescent Brain Cognitive Development Study. *Neuroimage* **202**, 116091 (2019).

83. J. Jovicich, S. Czanner, D. Greve, E. Haley, A. Van Der Kouwe, R. Gollub, D. Kennedy, F. Schmitt, G. Brown, J. MacFall, B. Fischl, A. Dale, Reliability in multi-site structural MRI studies: Effects of gradient non-linearity correction on phantom and human data. *Neuroimage* **30**, 436–443 (2006).

84. L. Wald, F. Schmitt, A. Dale, Systematic spatial distortion in MRI due to gradient non-linearities. *Neuroimage* **13**, 50 (2001).

85. W. Wells, Multi-modal volume registration by maximization of mutual information. *Med. Image Anal.* **1**, 35–51 (1996).

86. C. C. Chang, C. C. Chow, L. C. A. M. Tellier, S. Vattikuti, S. M. Purcell, J. J. Lee, Second-generation PLINK: Rising to the challenge of larger and richer datasets. *Gigascience* **4**, 7 (2015).

87. J. Mbatchou, L. Barnard, J. Backman, A. Marcketta, J. A. Kosmicki, A. Ziyatdinov, C. Benner, C. O'Dushlaine, M. Barber, B. Boutkov, L. Habegger, M. Ferreira, A. Baras, J. Reid, G. Abecasis, E. Maxwell, J. Marchini, Computationally efficient whole-genome regression for quantitative and binary traits. *Nat. Genet.* **53**, 1097–1103 (2021).

88. P. Cingolani, A. Platts, L. L. Wang, M. Coon, T. Nguyen, L. Wang, S. J. Land, X. Lu, D. M. Ruden, A program for annotating and predicting the effects of single nucleotide polymorphisms, SnpEff: SNPs in the genome of Drosophila melanogaster strain w^{1118}; iso-2; iso-3. *Fly* **6**, 80–92 (2012).

89. E. Sanderson, M. M. Glymour, M. V. Holmes, H. Kang, J. Morrison, M. R. Munafò, T. Palmer, C. M. Schooling, C. Wallace, Q. Zhao, G. Davey Smith, Mendelian randomization. *Nat. Rev. Methods Primer* **2**, 6 (2022).

90. A. Okbay, Y. Wu, N. Wang, H. Jayashankar, M. Bennett, S. M. Nehzati, J. Sidorenko, H. Kweon, G. Goldman, T. Gjorgjieva, Y. Jiang, B. Hicks, C. Tian, D. A. Hinds, R. Ahlskog, P. K. E. Magnusson, S. Oskarsson, C. Hayward, A. Campbell, D. J. Porteous, J. Freese, P. Herd; 23andMe Research Team; Social Science Genetic Association Consortium, C. Watson, J. Jala, D. Conley, P. D. Koellinger, M. Johannesson, D. Laibson, M. N. Meyer, J. J. Lee, A. Kong, L. Yengo, D. Cesarini, P. Turley, P. M. Visscher, J. P. Beauchamp, D. J. Benjamin, A. I. Young, Polygenic prediction of educational attainment within and between families from genome-wide association analyses in 3 million individuals. *Nat. Genet.* **54**, 437–449 (2022).

91. C. A. de Leeuw, J. M. Mooij, T. Heskes, D. Posthuma, MAGMA: Generalized gene-set analysis of GWAS data. *PLOS Comput. Biol.* **11**, e1004219 (2015).

92. K. Watanabe, M. Umićević Mirkov, C. A. de Leeuw, M. P. van den Heuvel, D. Posthuma, Genetic mapping of cell type specificity for complex traits. *Nat. Commun.* **10**, 3222 (2019).

93. H. K. Finucane, B. Bulik-Sullivan, A. Gusev, G. Trynka, Y. Reshef, P. R. Loh, V. Anttila, H. Xu, C. Zang, K. Farh, S. Ripke, F. R. Day, S. Purcell, E. Stahl, S. Lindstrom, J. R. B. Perry, Y. Okada, S. Raychaudhuri, M. J. Daly, N. Patterson, B. M. Neale, A. L. Price, Partitioning heritability by functional annotation using genome-wide association summary statistics. *Nat. Genet.* **47**, 1228–1235 (2015).

94. Roadmap Epigenomics Consortium, A. Kundaje, W. Meuleman, J. Ernst, M. Bilenky, A. Yen, A. Heravi-Moussavi, P. Kheradpour, Z. Zhang, J. Wang, M. J. Ziller, V. Amin, J. W. Whitaker, M. D. Schultz, L. D. Ward, A. Sarkar, G. Quon, R. S. Sandstrom, M. L. Eaton, Y.-C. Wu, A. R. Pfenning, X. Wang, M. Claussnitzer, Y. Liu, C. Coarfa, R. A. Harris, N.

Shoresh, C. B. Epstein, E. Gjoneska, D. Leung, W. Xie, R. D. Hawkins, R. Lister, C. Hong, P. Gascard, A. J. Mungall, R. Moore, E. Chuah, A. Tam, T. K. Canfield, R. S. Hansen, R. Kaul, P. J. Sabo, M. S. Bansal, A. Carles, J. R. Dixon, K.-H. Farh, S. Feizi, R. Karlic, A.-R. Kim, A. Kulkarni, D. Li, R. Lowdon, G. Elliott, T. R. Mercer, S. J. Neph, V. Onuchic, P. Polak, N. Rajagopal, P. Ray, R. C. Sallari, K. T. Siebenthall, N. A. Sinnott-Armstrong, M. Stevens, R. E. Thurman, J. Wu, B. Zhang, X. Zhou, A. E. Beaudet, L. A. Boyer, P. L. De Jager, P. J. Farnham, S. J. Fisher, D. Haussler, S. J. M. Jones, W. Li, M. A. Marra, M. T. McManus, S. Sunyaev, J. A. Thomson, T. D. Tlsty, L.-H. Tsai, W. Wang, R. A. Waterland, M. Q. Zhang, L. H. Chadwick, B. E. Bernstein, J. F. Costello, J. R. Ecker, M. Hirst, A. Meissner, A. Milosavljevic, B. Ren, J. A. Stamatoyannopoulos, T. Wang, M. Kellis, Integrative analysis of 111 reference human epigenomes. *Nature* **518**, 317–329 (2015).

95. The ENCODE Project Consortium, An integrated encyclopedia of DNA elements in the human genome. *Nature* **489**, 57–74 (2012).

96. T. Ge, C. Y. Chen, Y. Ni, Y. C. A. Feng, J. W. Smoller, Polygenic prediction via Bayesian regression and continuous shrinkage priors. *Nat. Commun.* **10**, 1776 (2019).

97. M. A. Mansournia, D. G. Altman, Inverse probability weighting. *BMJ* **352**, i189 (2016).

98. A. P. Boughton, R. P. Welch, M. Flickinger, P. VandeHaar, D. Taliun, G. R. Abecasis, M. Boehnke, LocusZoom.js: Interactive and embeddable visualization of genetic association study results. *Bioinformatics* **37**, 3017–3018 (2021).

99. B. Fischl, D. H. Salat, E. Busa, M. Albert, M. Dieterich, C. Haselgrove, A. Van Der Kouwe, R. Killiany, D. Kennedy, S. Klaveness, A. Montillo, N. Makris, B. Rosen, A. M. Dale, Whole brain segmentation: Automated labeling of neuroanatomical structures in the human brain. *Neuron* **33**, 341–355 (2002).

100. W. M. Pauli, A. N. Nili, J. Michael Tyszka, A high-resolution probabilistic in vivo atlas of human subcortical brain nuclei. *Sci. Data* **5**, 180063 (2018).
